# Arbuscular mycorrhizal symbiosis affects the grain proteome of *Zea mays*: a field study

**DOI:** 10.1038/srep26439

**Published:** 2016-05-24

**Authors:** Elisa Bona, Alessio Scarafoni, Francesco Marsano, Lara Boatti, Andrea Copetta, Nadia Massa, Elisa Gamalero, Giovanni D’Agostino, Patrizia Cesaro, Maria Cavaletto, Graziella Berta

**Affiliations:** 1 Dipartimento di Scienze ed Innovazione Tecnologica, Università del Piemonte Orientale Amedeo Avogadro, Viale Teresa Michel, 11, 15121 Alessandria, Italy; 2 Dipartimento di Scienze per gli Alimenti, la Nutrizione e l’Ambiente, Università di Milano, Via Celoria, 2, 20133 Milano, Italy; 3 Mybasol srl, Via Gentilini 3, 15121 Alessandria, Italy

## Abstract

Maize is one of the most important crops worldwide and is strongly dependent on arbuscular mycorrhiza (AM) fungi, organisms that form a mutualistic association with land plants. In maize, AM symbiosis enhances spike dry weight, spike length, spike circumference, and the dry weight and dimensions of the grain. Notwithstanding its ubiquitous nature, the detailed relationship between AM fungal colonization and plant development is not completely understood. To facilitate a better understanding of the effects of AM fungi on plants, the work reported here assessed the effects of a consortium of AM fungi on the kernel proteome of maize, cultivated in open-field conditions. To our knowledge, this is the first report of the modulation of a plant seed proteome following AM fungal inoculation in the field. Here, it was found that AM fungi modify the maize seed proteome by up-regulating enzymes involved in energetic metabolism, embryo development, nucleotide metabolism, seed storage and stress responses.

Mycorrhiza represent a widespread mutualistic association between most land plants, including agriculturally relevant species^1^, and arbuscular mycorrhizal (AM) fungi, a monophyletic group of soil microorganims belonging to the Glomeromycota phylum^2^. When the symbiosis is established, the fungus grows within the cells of the roots forming arbuscules, which is the main site of nutrient exchange between the fungus and the plant. Moreover, the fungus develops an extensive extraradical mycelium that enhances the absorption ability of the plant root system^3^. The success of AM symbiosis is mostly due to the benefits that both partners gain from this relationship. The fungal partner takes up both water and mineral nutrients, mainly phosphorus and nitrogen, from the soil, through its mycelium, and transfers these compounds via the symbiotic interface to the plant root cells^4^,^5^. In turn, the plant supplies the fungus with about 10–20% of the plant’s photosynthates. This symbiosis directly influences plant responses and plant physiology, both in the target organ (roots) and in shoots, and as recently demonstrated in fruits^6^,^7^,^8^,^9^. As a consequence of this plant-fungal relationship, the AM symbiosis enhanced yield and improved fruit quality (taste and vitamin concentration) in strawberry fruits^6^,^7^,^8^,^9^; modulated sugar and carotenoid concentrations in tomato fruits^10^; increased the accumulation of carotenoids, chlorophylls and tocopherol in green and red leaf lettuces^11^; improved the yield and quality of saffron (*Crocus sativus* L.)^12^; increased growth, flavour and yield in *Allium sativum* L. cultivated in field conditions^13^; impacted the phenolic content and antioxidant properties of artichoke leaves^14^; and modulated essential oil production in *Artemisia annua* L.^15^ and in *Ocimum basilicum* L.^16^,^17^. A large body of evidence has shown that the protein profile of *Pteris vittata* and *Medicago truncatula* root^18^,^19^,^20^, and *P. vittata, Populus alba* and *Zea mays* leaf^21^,^22^,^23^, are affected by AM symbiosis. The above mentioned studies found that the plant traits that were positively affected by AM fungi included photosynthesis, carbon fixation and energy production in leaves, and glycolysis in roots. Notwithstanding these results, there is very little data regarding the impact of AM fungi on the plant seed proteome.

Maize (*Zea mays* L.) is one of the most important crops worldwide. Its economic and nutritional value is mainly due to the high starch content that represent about 75% of mature seed weight^24^. Maize is strongly dependent on mycorrhizae^25^. For example, in maize, the AM symbiosis enhances spike dry weight, spike length, spike circumference, and the dry weight and dimensions of the grain^26^. To better understand the effect of AM fungi on maize, the present study was undertaken with the aim of assessing the effects of a consortium of AM fungi on the maize kernel proteome, cultivated in open-field conditions.

## Results and Discussion

According to FAO, cereals are defined as a group of species generally, but not exclusively, belonging to the gramineous family (i.e. Poaceae) that produce dry seeds rich in starch. Of the cereals, the most commonly cultivated plant is maize; this is because of its multiple uses, as a food and feed, and as a source of raw materials for industrial applications, such as the production of bioplastics and biofuels. While it has been known for some time that maize is a mycorrhiza-dependent plant^25^, the effects of the interaction between AM fungi and seeds has not been exhaustively investigated. To the best of our knowledge, this is the first report describing the effects of AM fungal inoculation, in open field conditions, on seed protein composition using a proteomic approach. As reported previously^26^, maize plant roots are naturally colonized by autochthonous AM fungi. Perhaps not surprisingly, using field soil, the frequency and the intensity of the mycorrhizal colonization, as well as arbuscule abundance, were significantly higher in plants treated with an AM fungal inoculum than in control plants. For example, the mycorrhizal colonization degree (M%) in mycorrhiza inoculated plants (MIC) was 27.7 ± 4.6 while in control plants (CTRL) was 6.9 ± 0.9. Moreover, it has been demonstrated^26^ that AM fungal inoculation increased maize plant growth and grain yield. In particular, spikes produced by MIC plants were greater in both number and size than those produced by the CTRL plants. In addition, the number, the dry weight, the size and the morphology of kernels were also increased by mycorrhizal inocula.

In the present work, seeds from CTRL and MIC plants collected 20 days after flowering (DAF) and 60 DAF were used for biochemical and proteomic analyses. Maize seeds accumulate large amount of proteins beginning with the first phases of seed development (Table 1), however, the differences between the amounts of protein in four protein different classes in CTRL and MIC plants were not statistically significant (p** **> 0.05). This data is consistent with previous results^27^ in a study examining the early accumulation of proteins in developing kernels. We subsequently investigated the possible modulation of the relative amounts of the different seed protein classes as a consequence of the AM inoculation. The results are shown in Table 1. According to Osborne^28^, seed proteins may be classified into groups according to their solubility in a series of solvents as albumins (water), globulins (dilute aqueous salt solutions), prolamins (alcohol solutions) and glutelins (dilute alkali or acid). At 20 DAF, the albumin fraction represented the majority of the kernel proteins in both CTRL and MIC samples. At 60 DAF, when the seeds were fully mature, the albumin content was significantly reduced with more albumin fraction in CTRL than in MIC plants. The albumin fraction consits largely of metabolically active proteins and, thus, a greater amount of this kind of proteins was expected in the first sampling, when intensive seed filling occurs. Mature seeds contain a limited set of enzymes, with the majority of them necessary to sustain the ability of the seed to resume metabolic activities during germination. Globulins, which are deposited in the embryo and in the outer aleurone layer^29^, were accumulated in a larger amount in mature seeds than in the 20 DAF seeds; they were positively affected by AM treatment. The globulin fraction is a heterogeneous group that includes the 7S proteins (also called vicilins), which include the major maize storage protein globulin^30^, the 11S storage proteins (legumins), various kind of defence proteins and the lipid transfer protein (LTP), one of the main maize seed allergens^31^. Zeins are prolamins and are the main storage proteins in the starchy endosperm tissue^32^, accounting for about 45–50% of the total maize seed proteins^33^. Zeins are classified according to structural features as α-, β-, γ- and ω-zeins, the first of which is the most abundant and is encoded by at least four gene families^34^. It has been shown that the accumulation of zeins begins very early (15 DAF) and continues through most of seed development^34^. In these experiments, the presence of zeins reached about 32% in 20 DAF CTRL plant. Moreover, AM treatment boosted their relative amount up to 42%. In mature seeds, zeins accounted for about 45–47% of the total seed proteins, independent of the presence of the AM fungal symbiosis. Glutelins, together with zeins, are major storage proteins of the seed endosperm. They represent the second largest protein fraction in mature seeds and show sequence similarities to other cereal storage proteins, such as gliadins and glutenins^35^. Overall, these results indicate that AM treatment does not greatly influence the accumulation of the analysed protein fractions either at the beginning of the seed filling process or in mature seeds.

**Figure 1:**
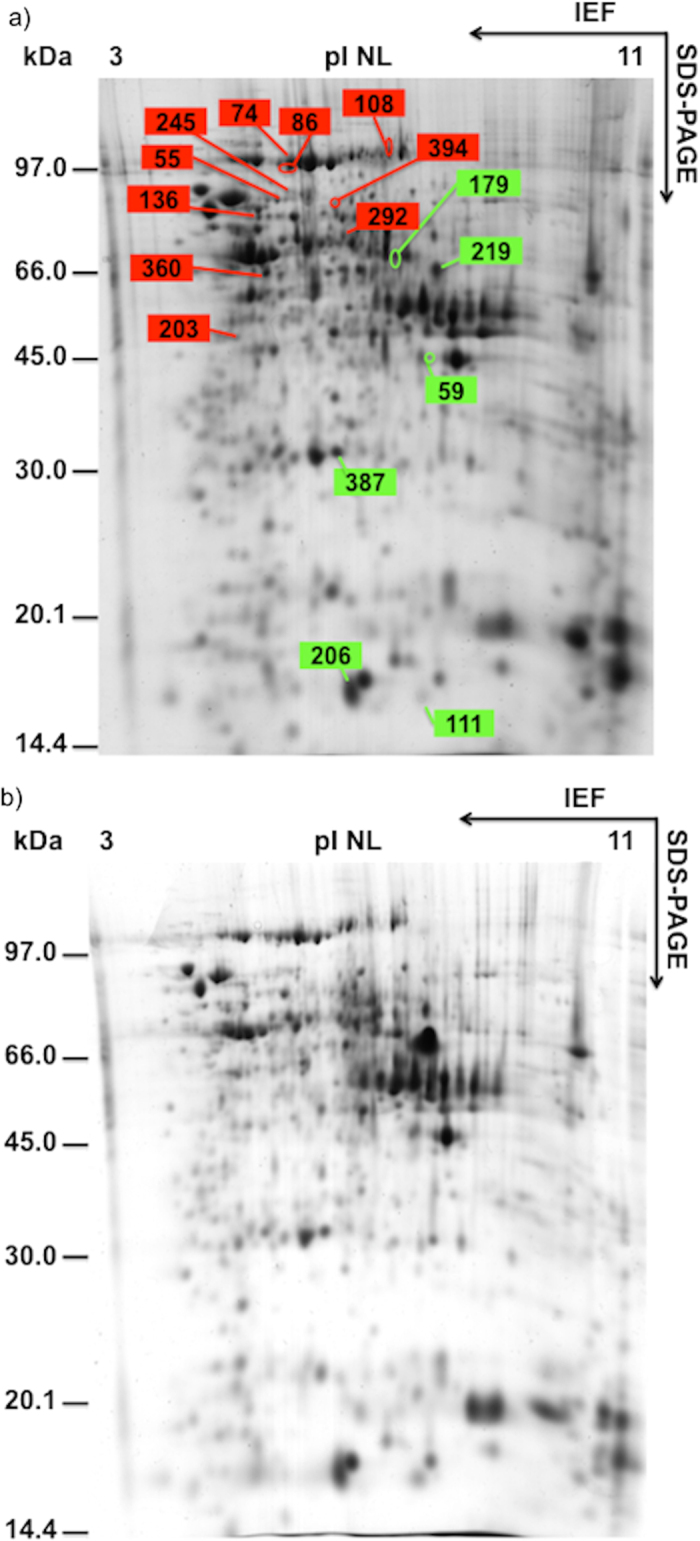
((**a**) (CTRL), (**b**) (MIC)). 2D maps of seed proteins extracted from seeds at 20 days after flowering (DAF), stained with Colloidal Coomassie. The assigned spots in the map were those modulated by AM symbiosis (green, up-regulated spots; red, down-regulated spots).

**Figure 2:**
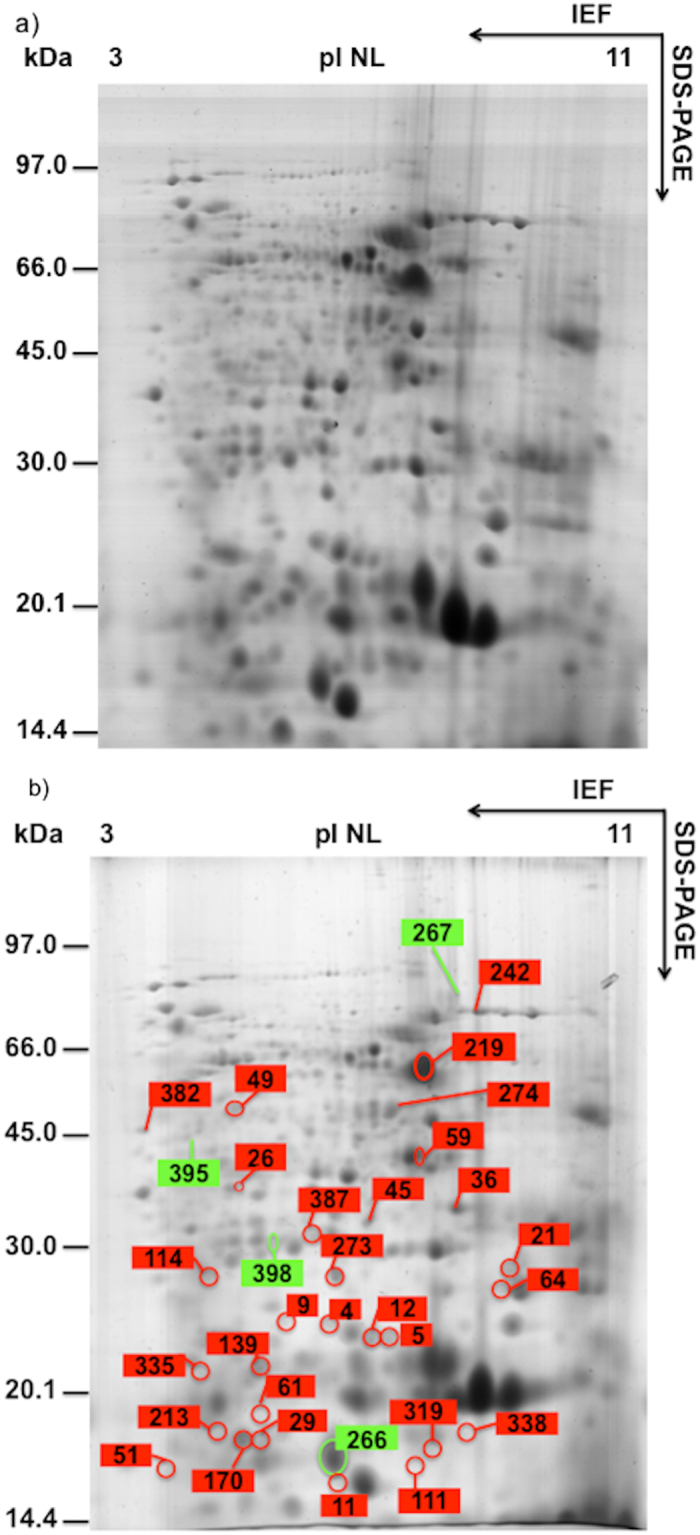
((**a**) (CTRL), (**b**) (MIC)). 2D maps of seed proteins extracted from seeds at 60 days after flowering (DAF), stained with Colloidal Coomassie. The assigned spots in the map were those modulated by AM symbiosis (green, up-regulated spots; red, down-regulated spots).

The 2D maps of seed proteins, stained with Colloidal Coomassie, showed a mean of 750 reproducible spots (Figs 1(a,b) and 2(a,b)). Significant variations were detected for 141 spots, of which 131 were MS/MS identified (93%). Table 2 lists the information regarding modulated proteins: spot number, number of identified peptides, sequence coverage, optical density variation using colour code, ANOVA P-value, protein name and Blast results when present, theorical molecular weight and pI, accession number and reference organism, and the biological process in which the identified protein is believed to be involved. Supplementary Tables S1, S2 and S3 list optical density raw data (as well as the statistical differences and P values), MS/MS results and BLAST results, respectively.

Despite the fact that the root is the organ that is colonised by AM fungi, the physiology of the entire plant is affected by the symbiosis, with interaction with the fungus having been reported to modulate photosynthesis, leaf hydration, reproduction and fruit quality in both maize and other plant species^6^,^26^,^36^,^37^. This modulation of the plant physiology changes according to the different stages of a plant’s development and which plant organs are being analyzed. As shown in Table 2, the maize seed proteome of mycorrhizal plants differed either at the beginning of seed development (20 DAF) or at the end of maturation (60 DAF).

### Effects during seed development

A detailed examination of the results revealed that at 20 DAF the AM symbiosis induced the up-regulation of enzymes involved in energetic metabolism, the latter stages of embryo development, nucleotide metabolism, seed storage and stress responses. AM fungi enhances primary metabolism by up-regulating ATP synthase (spot 219); this protein is a key enzyme whose expression is linked to respiratory and photosynthetic phosphorylation, both of which are major processes in the energetic metabolism of above-ground plant tissues. The up-regulation of the cytosolic ascorbate peroxidase (spot 387), a major enzyme involved in detoxification of hydrogen peroxide, was also induced by AM fungi; its expression may be linked with embryo development. Thus, Méchin *et al.*^24^ reported that this enzyme is modulated in maize seeds 14 days after pollination^24^, whereas Finnie *et al.*^38^ showed that a cytosolic form of this enzyme was only detectable in an early developmental stage of barley seeds.

The overexpression of nucleoside diphosphate kinase 1 (spot 111) may lead to reduced constitutive reactive oxygen species (ROS) levels and enhaced tolerance to multiple environmental stress^39^. The expression of nucleoside diphosphate kinase has been reported to increase in response to drought and salinity, thus it is expected to accumulate in the late phases of embryogenesis. This enzyme also plays significant roles in hormone responses, heat stress and, in general, growth and development^39^. An increase of ROS could induce the observed increase of heat shock 70 kDa protein (spot 55) expression.

The accumulation of Legumin 1 (spot 179), a storage protein found in maize seeds, is a confirmation of the data of globulin quantification and is linked with the seed storage process. The AM symbiosis induced down regulation of three starch granule-associated proteins, namely phosphoglucomutase 2 (spot 245), phosphoglycerate mutase (spot 136), and a pyruvate ortophosphate dikinase (spot 74) as well as seven proteins involved in cellular metabolic processes, an elongation factor 2 (spot 108), a translational initiation factor eIF-4A (spot 360), an ATP-dependent Clp protease ATP-binding subunit (spot 86), a ketol-acid reductoisomerase (spot 292), a stress responsive protein (spot 59) and succinate dehydrogenase flavoprotein subunit (spot 394). In order to use their stored carbon reserves, plants must be able to degrade their starch granules to oligosaccharides and monosaccharides. In particular, as previously reported, phosphoglucomutase 2 converts glucose 1-phosphate to glucose 6-phosphate facilitating the use of this compound in glycolysis^40^. The orthophosphate dikinase partly controls the composition of the storage protein fractions and the starch-protein balance^24^. The classical role of orthophosphate dikinase in both C3 and C4 plants involves catalyzing the reversible reaction of pyruvate, ATP and phosphate to phosphoenol-pyruvate, AMP and diphosphate. In rice, the expression of ortophosphate dikinase was found to be highest at 5–15 days after pollination; after that time this enzyme was likely rapidly degraded or inactivated through phosphorylation^41^. This pool of inactivated orthophosphate dikinase was also present in mature seeds, suggesting a role in developmental processes during seed germination^39^,^42^.

### Effects at seed maturation

At 60 DAF, the presence of AM fungi induced the modulation of 33 maize seed proteins, 4 up-regulated and 29 down-regulated. The degradation of the reserves (starch and storage proteins) and of some functional proteins could provide enough energy and amino acids for seed germination and for embryo development^43^. This could explain the up-regulation of proteasome proteins (spot 398) and the strong down regulation of different enzymes in AM-treated plants, such as adenine phosphoribosyl transferase (spot 114), ATP synthase beta chain (spot 219), sorbitol dehydrogenase (spot 274), prohibitin 3 and 2 (spots 36 and 45, respectively), two actin depolymerizing factor (spots 29 and 70), ankyrin repeat domain-containing protein 2 (spot 382), and late embryogenesis abundant protein Leal 4-A (spot 61).

Maize seeds acquire the ability to germinate during the stage of maturation drying^44^. The decreased water content plays an important role for the seeds to acquire the ability to germinate and for protection against fungal infection. Germination is a potentially stressful process and the reactivation of metabolism may provide an important source of ROS^44^. This can explain the increase in the abundance of proteins linked to the ROS response and AM symbiosis an overexpression of the same proteins such as salt tolerance protein (395) and down-regulation of APx-1 cytosolic ascorbate peroxidase (387), splicing factor (9), two spots belonging to nucleoside diphosphate kinase 1 (spots 111 and 319) that can lead to decreased constitutive reactive oxygen species (ROS) levels and enhanced tolerance to multiple environmental stress^39^, a peroxiredoxin (335), superoxide dismutase3 (273), a 22 kDa heat shock protein (4), the activator of a 90 kDa heat shock protein ATPase (49), and a stress responsive protein (59). The down regulation, in AM-treated seeds, of different isoforms of storage proteins such as Zein-alpha 19D1 precursor (21), vicilin-like embryo storage protein (spots 26, 51 and 242), z1B alpha zein protein (64) and lactoylglutathione lyase (213) could be linked with the seed protein turnover induced by embryo maturation.

### Ripening effect on maize seed proteome

**Figure 3:**
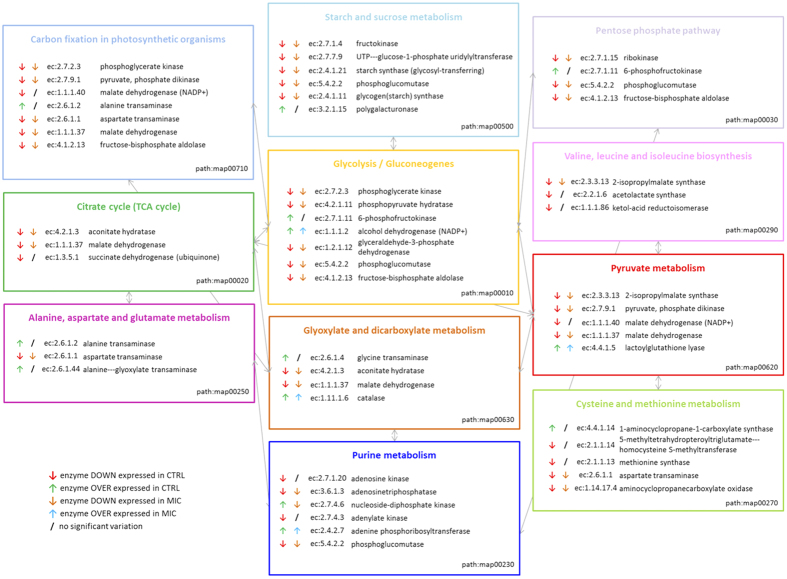
Kegg maps summarizing the main biochemical pathways involved in proteome modification during ripening: carbon fixation, starch and sucrose metabolism, pentose phosphate pathway, citrate cycle, glycolysis/gluconeogenesis, valine, leucine and isoleucine biosynthesis, alanine, aspartate and glutamate metabolism, glyoxylate and dicarboxylate metabolism, pyruvate metabolism, purine metabolism, cysteine and methionine metabolism.

Maize is an excellent model for research on cereal seed development because of the relatively large size of both its embryo and endosperm. Despite the importance of seed maturation information for agricultural purpose, there is scant data available in literature regarding the effects of root AM fungal inoculation on seed maturation. Kegg maps (Fig. 3 and Table S4) summarizes the main biochemical pathways involved in the maize seed proteome modification during ripening, i.e. carbon fixation; starch and sucrose metabolism; the pentose phosphate pathway; the citrate cycle; glycolysis/gluconeogenesis; valine, leucine and isoleucine biosynthesis; alanine, aspartate and glutamate metabolism; glyoxylate and dicarboxylate metabolism; pyruvate metabolism; purine metabolism; and cysteine and methionine metabolism. Both in control and in mycorrhizal plants, seed maturation induced the same proteome evolution with the exception of: malate dehydrogenase, succinate dehydrogenase, adenosine kinase, adenylate kinase, acetolactate synthase, ketol-acid reductoisomerase, homocysteine S-methyltransferase and methionine synthase (down-regulated in CTRL and not modified in MIC); alanine transaminase, alanine-glyoxylate transaminase, polygalacturonase, 6-phosphofructokinase, glycine transaminase, 1-aminocyclopropane-1-carboxylate synthase (up-regulated in CTRL and not modified in MIC); nucleoside diphosphate kinase (up-regulated in CTRL and down-regulated in MIC); and zein-protein precursor (up-regulated in MIC and not modified in CTRL). These changes in protein abundance could be linked with the higher content of starch in the seeds of plants treated with mycorrhizal fungi.

Our results are in agreement with those of Huang *et al.*^45^, who reported, on the basis of the metabolic and functional features of maize embryos, the identification of proteins classified into 7 major categories belonging to 3 functional groups: protein metabolism (26%), stress response (21%) and carbohydrate and energy metabolism (17%). At maturity, the maize seed accumulates large amounts of starch and storage proteins^45^. However, proteins involved in stress response (24%) were often up-regulated during seed maturation^45^.

A large body of literature describes the effects of AM fungi on the physiology of whole plants^6^,^7^,^16^,^17^, with a particular focus on fruit composition. The work presented here is a first step in filling the gap in the knowledge of the effect of AM fungi on seed composition. In the work described here, it was demonstrated that AM fungi strongly modify the seed proteome, particularly up-regulating enzymes involved in energy metabolism, embryo development, nucleotide metabolism, seed storage and stress responses.

Finally, this work underlines the importance of using soil microorganisms as inocula in field production to sustainably improve crop quality.

## Materials and Methods

### Experimental Field, Plant Growth And Seed Sampling

The experiment was conducted as described in Berta *et al.*^26^. In accordance with standard agricultural practices, field soil was fertilized with potassium sulfate (400 Kg/ha) and 18/46 N/P (350 Kg/ha). Corn seeds (*Zea mays* var. Ostiglia) were sown on 14th March 2013 in double rows. Three double lines (200 plants each) were treated with AM inoculum. An uninoculated double row was selected ramdomly as a control. The AM inoculum (Mybasol s.r.l., Alessandria, Italy), consisted of root fragments, spores, and hyphae of *Rhizophagus intraradices*, *Glomus aggregatum*, *Glomus viscosum*, *Claroideoglomus etunicatum*, and *Claroideoglomus claroideum* produced on sorghum, containing about 85,000 infective propagules l^**−**1^, was applied every 6 cm using a drip irrigation tube. During the growth period, diseases and insects were adequately controlled. Caryopsis harvest started from the 26^th^ of July, 20 DAF and ended on the 4^th^ of September, 60 DAF. During each sampling date, tillers of three kernels of control (CTRL) and mycorrhizal (MIC) plants were open, 25 g of grains from half of each ear, were collected, immediately frozen in liquid nitrogen, and stored at −80 °C.

Five, randomly selected, roots per treatment were used to evaluate frequency (F%), mycorrhizal degree (M%) and arbuscule abundance (A%)^46^.

### Selective extraction of different protein classes

Ten grams of seeds were ground in a mortar using liquid nitrogen and extracted twice with milliQ water containing a protease inhibitor cocktail (Sigma-Aldrich), in the ratio 1:10 (p/v), at 4 °C for 2 hours. The slurries were centrifuged at 10,000 × *g* for 15 min. The two supernatants (albumin fraction) were pooled and stored at −20 °C, whereas the pellet was extracted twice with Tris-HCl 50 mM, pH 8.0, containing 0.3 M NaCl. The slurries were centrifuged at 10,000 ×*g* for 15 min. The supernatants (globulin fraction) were pooled and stored at −20 °C. The insoluble pellet was extracted twice with 70% ethanol containing 0.2% 2-mercaptoethanol. After stirring for 3 hours at 4 °C, the suspension was centrifuged at 10,000 × *g* for 30 min at 4 °C. The supernatants (prolamin fraction), were pooled and dried with a Rotavapor device. The insoluble pellet was then resuspended in 0.1 M NaOH to extract the glutelin fraction at 4 °C for 2 hours.

Three biological replicates were analysed in triplicate. Protein concentrations were determined according to Bradford^47^.

Seed water content was determined by placing one gram of ground seeds at 110 °C and then in a jar containing silica gel. Samples were analyzed twice in duplicate.

### Proteomic analysis

Proteins were extracted according to Bona *et al.*^48^. The pellet was resuspended in 1 ml of solubilization buffer containing 7 M urea, 2M thiourea, 4% CHAPS, 100 mM DTT, 1% IPG buffer (3–11 NL) and quantified by the method of Bradford^47^. Aliquots of 700 μg of protein extracts were mixed with a rehydration buffer (8 M urea, 4% w/v CHAPS, 18 mM DTT, 0.5% 3–11 IPG Buffer), focused at 60 kVh at 20 °C on precast 13 cm NL pH 3–11 strips in an IPG-Phor unit (GE Healthcare Bio-Sciences) and separated on 12% gels at 10 °C under constant amperage (30 mA per gel) with a Protean Plus Dodeca gel (BioRad). At least ten replicates were run, two analytical replicates per five biological replicates.

Gels were stained according to Candiano *et al.*^49^, and then digitized in a GS 710 densitometer (Bio-Rad). The gel images were analyzed using SameSpot (Progenesis v. 2006) (build 3419. 12870). Differential expression analysis was performed: i) comparing the quantity of matched spots in the CTRL at 20 DAF versus MIC plants at 20 DAF (to evaluate the effect of AM fungus addition at the beginning of maturation); ii) comparing the quantity of matched spots in the CTRL at 60 DAF versus MIC plants at 60 DAF (to evaluate the effect of AM fungus addition at the end of the maturation period); iii) comparing the quantity of matched spots in the CTRL at 20 DAF versus CTRL plants at 60 DAF (to evaluate protein changes during maturation); iv) comparing the quantity of matched spots in the MIC plants at 20 DAF versus MIC plants at 60 DAF (to evaluate protein changes during maturation in AM plants). The software created a quantitative table with all normalized optical spot densities that allowed us to perform an analysis of variance to detect statistical differences between the quantitation of the same spot in all replicates.

### Protein identification by nano-LC-Q-TOF MS/MS

For MS analysis, spots of interest were cut from the gel and destained overnight with a solution of 25 mM ammonium bicarbonate and 50% acetonitrile. The proteins were digested with trypsin (Roche, Segrate, Milano, Italy) in-gel digested as described by Hellmann *et al.*^50^. All nano-HPLC-MS/MS experiments were performed on a Q-TOF mass spectrometer Q-Star XL (AB Sciex, Concord, Ontario, Canada) controlled by the Analyst QS 1.1 software (AB Sciex) connected to an Ultimate 3000 nano-HPLC system. The peptide pellets were resuspended in 10 μl of solvent A (95% v/v water, 5% v/v acetonitrile, 0.1%v/v formic acid). Five microliters of each sample were loaded onto the precolumn, 300 μm i.d. × 5 mm, C18 PepMap, 5 μm beads, 100 Å, (LC-Packings) and washed for 5 min using a flow rate of 40 μl min^−1^ solvent A. The peptides were subsequently eluted at 300 nl min^−1^ from the precolumn over an analytical column, 15 cm × 75 μm, C18 PepMap100, 3 μm beads, 100 Å (LCPackings) using a 35 min gradient from 5 to 60% solvent B (5% v/v water, 95% v/v acetonitrile, 0.1% v/v formic acid) delivered at 300 μl min^−1^. The analytical column was connected with a 15 μm inner diameter Silica Tip (Pico Tip) nanospray emitter (New Objective, Woburn, MA). The spray voltage (set between 1800 and 2000 V) was applied to the emitter through a stainless steel union and tuned to get the best signal intensity using a standard BSA tryptic digest before every sample’s batch submission. The QStar-XL was operated in information-dependent acquisition (IDA) mode. Mass spectra were acquired from 400 to 1800 m/z. The two most intense ions with charge states between 1 and 4 in each survey scan were selected for the MS/MS experiment. MS/MS data were acquired from 60 to 1800 m/z. Each acquisition cycle was comprised of a 1 s MS and a 3 s MS/MS. The MS to MS/MS switch threshold was set to 15 counts per second (c.p.s.). All precursor ions subjected to MS/MS in the previous cycle were automatically excluded for 60 s using a 3 amu.

### Homology-driven proteomics

Mascot Distiller (Matrix Science, London, UK) was used to create peak lists from MS and MS/MS raw data. Mascot Server (Matrix Science) was used for database searching versus NCBInr. The last check for proteins homology assignments was made versus NCBInr 20151214 (78002046 sequences; 28422168805 residues). Carbamidomethylation of cysteine residues, oxidation of methionine, deamidation of asparagine and glutamine were set as possible variable modifications and trypsin was selected as the protease. One missed cleavage site was allowed, and the peptide MS and MS/MS tolerance was set respectively to 100 ppm and 0.2 Da. Positive identifications were assigned with a minimum of two unique peptides with at least one peptide having a significant ion score (underlined in red in Table S2 in the supporting information). Considering the scarce number of corn sequences in the databases, if we obtained an automatic hit without a significant score, sequence tags were manually interpreted from the ESI-MS/MS spectra to confirm the hypothetical assignment. We also accepted hits identified by at least one peptide with a significant ion score according to the MASCOT MS/MS ion search algorithm as being confident assignments. When a protein has only one spectrum with a significant Mascot score, but in the results there are more spectra with lower scores, they were manually inspected and if they had a pattern compatible with the theoretical peptide, they were considered for homology searching. The sequence obtained from the manually reconstructed peptide was submitted to MS homology and if the first positive hit was the same protein or a homologue sequence of the one automatically recognized, the peptide was inserted in the table as assigned to the protein. This approach allow the use of partial “*de novo*” sequences that can be more fitting to the sequences in the database^51^.

### Blast2GO data analysis

To perform the Blast2GO analysis (http://www.blast2go.com/b2ghome) we downloaded the protein FASTA sequences from http://www.ncbi.nlm.nih.gov using the GI code ID.

Data analysis was performed with Blast2GO standard parameters.

The EC annotations, obtained by mapping from equivalent GO annotations, were visualized reconstructing the structure of the Gene Ontology relationships and ECs on KEGG maps (http://www.genome.jp/kegg). In KEGG maps were displayed the enzymatic functions of sequences in the context of the metabolic pathways in which they participate.

### Statistical analysis

Data were analyzed by a one-way ANOVA followed by Fisher’s test with cut-off significance at p = 0.05 using Stat View 4.5 (Abacus Concepts) software.

## Additional Information

**How to cite this article**: Bona, E. *et al.* Arbuscular mycorrhizal symbiosis affects the grain proteome of *Zea mays*: a field study. *Sci. Rep.* **6**, 26439; doi: 10.1038/srep26439 (2016).

## Supplementary Material

Supplementary Information

## Figures and Tables

**Table 1:** Relative amounts (%) of maize seed proteins.

	20 DAF		60 DAF	
	CTRL	MIC	CTRL	MIC
Albumin	34.7 ± 5.3 aA	31.1 ± 6.2 bA	15.1 ± 1.7 aB	10.3 ± 2.4 bB
Globulin	8.1 ± 4.4 aA	5.3 ± 2.6 aA	9.9 ± 2.2 aA	16.8 ± 2.2 bB
Prolamins	32.1 ± 6.1 aA	41.9 ± 5.7 aA	44.6 ± 3.1 aB	47.4 ± 2.6 aA
Glutelins	20.0 ± 5.4 aA	21.6 ± 5.2 aA	27.9 ± 3.4 aA	25.2 ± 3.9 aA

Seed proteins were classified in groups according to their solubility in a series of solvents including albumin (soluble in water), globulins (soluble in dilute aqueous salt solution), prolamins (soluble in alcohol solution) and glutelins (soluble in dilute alkali). Data are expressed as means ± standard error (Three biological samples were analysed twice in duplicate). ANOVA followed by Fisher’s probable least-squares difference test used a cut-off significance at p = 0.05. Different letters indicate significantly different values based on one-way ANOVA (P < 0.05). Small letters indicate comparison between treatments (CTRL vs MIC) at the same time (20 or 60 DAF); capital letters indicate comparison between different times (20 DAF vs 60 DAF) in the same treatment (CTRL or MIC).

**Table 2:** Information regarding modulated proteins spot number, number of identified peptides, sequence coverage, optical density variation using colour code, ANOVA P-value, protein name and Blast results when present, theoretical molecular weight and pI (experimental pI data were not reported because the isoelectrofocusing was performed on non-linear IPG strips and image analysis software was not able to precisely calculate the pI), accession number and reference organism, biological process in which the identified protein was involved.

Spot	N. Peptides	Seq. Coverage	Fungus effect at 20 DAF	Fungus effect at 60 DAF	Ripening effect on CTRL	Ripening effect on MIC	P-Value	Protein name/Blast result	Mr (kDa)/pI Theor	AC number/reference organism	Biological process
**114**	8	47%		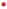	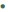	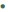	<0.0001	Unknown/Adenine phosphoribosyl transferase	19336/5.14	gi|194701624/*Zea mays*	Adenine salvage
**381**	11	18%			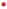		0.0357	Acetolactate synthase 1	68887/6.69	gi|75102649/*Zea mays*	Amino-acid biosynthesis
**219**	7	22%	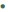	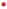	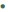	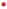	<0.0001	ATP synthase beta chain	45679/4.92	gi|149798689/*Eriosorus cheilanthoides*	ATP synthesis coupled proton transport
**388**	3	8%			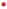		0.0344	IAA-glu synthetase	49679/5.75	gi|162460991/*Zea mays*	Auxin conjugation
**24**	5	7%			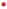	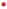	<0.0001	Putative aconitate hydratase	98021/5.67	gi|75225211/*Oryza sativa*	Carbohydrate metabolism
**164**	9	22%			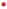	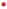	<0.0001	Phosphoglucomutase 2	63002/5.47	gi|162459678/*Zea mays*	Carbohydrate metabolism
**274**	12	34%		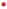		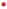	0.0135	Sorbitol dehydrogenase	39063/6.27	gi|77378040/*Zea mays*	Carbohydrate metabolism
**380**	13	37%			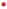	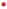	0.0018	Fructokinase 2	35459/5.34	gi|162460525/*Zea mays*	Carbohydrate metabolism
**54**	11	22%			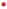	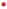	<0.0001	Phosphoglucomutase 1	63058/5.46	gi|162463106/*Zea mays*	Carbohydrate metabolism/glucose metabolism
**67**	17	36%			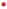	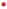	<0.0001	Phosphoglucomutase 2	63002/5.47	gi|162459678/*Zea mays*	Carbohydrate metabolism/glucose metabolism
**245**	3	8%	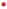		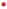		0.0272	Phosphoglucomutase 2	63002/5.47	gi|162459678/*Zea mays*	Carbohydrate metabolism/glucose metabolism
**112**	12	53%			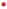	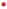	0.0002	Unknown/Aldolase 1	38566/7.52	gi|194690156/*Zea mays*	Carbohydrate metabolism/Glycolysis
**136**	16	41%	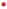		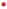		0.0002	Phosphoglycerate mutase	60592/5.29	gi|551288/*Zea mays*	Carbohydrate metabolism/Glycolysis
**145**	20	62%			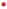	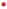	<0.0001	3-phosphoglycerate kinase	42413/5.65	gi|194707626/*Zea mays*	Carbohydrate metabolism/Glycolysis
**163**	14	63%			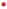	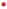	0.0002	Glyceraldehyde-3-phosphate dehydrogenase	24930/8.44	gi|293887/*Zea mays*	Carbohydrate metabolism/Glycolysis
**196**	18	61%			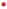	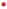	0.0004	Glyceroldehyde-3-phosphate dehydrogenase	36428/6.61	gi|162458671/*Zea mays*	Carbohydrate metabolism/Glycolysis
**205**	17	64%				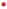	0.0045	Glyceroldehyde-3-phosphate dehydrogenase	36519/6.41	gi|162461501/*Zea mays*	Carbohydrate metabolism/Glycolysis
**249**	4	7%			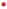	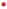	<0.0001	Phosphoglycerate mutase	60592/5.29	gi|551288/*Zea mays*	Carbohydrate metabolism/Glycolysis
**251**	10	49%			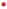	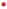	0.0003	Glyceroldehyde-3-phosphate dehydrogenase	36428/6.61	gi|162458671/*Zea mays*	Carbohydrate metabolism/Glycolysis
**278**	10	35%			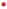	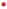	0.0070	Enolase1	48033/5.20	gi|162458207/*Zea mays*	Carbohydrate metabolism/Glycolysis
**305**	15	67%			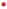	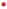	0.0002	Glyceraldehyde-3-phosphate dehydrogenase	24930/8.44	gi|293887/*Zea mays*	Carbohydrate metabolism/Glycolysis
**309**	4	9%			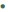		0.0112	Unknown/Phosphofructokinase	60980/5.96	gi|194700662/*Zea mays*	Carbohydrate metabolism/Glycolysis
**311**	17	51%			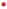	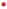	0.0013	Unknown/Phosphoglycerate kinase	42413/5.65	gi|194707626/*Zea mays*	Carbohydrate metabolism/Glycolysis
**321**	17	48%			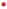	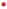	0.0079	Enolase1	48033/5.20	gi|162458207/*Zea mays*	Carbohydrate metabolism/Glycolysis
**326**	2	8%			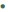	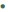	<0.0001	Enolase1	48033/5.20	gi|162458207/*Zea mays*	Carbohydrate metabolism/Glycolysis
**368**	10	31%			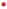	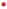	0.0100	Enolase2	48132/5.70	gi|162460735/*Zea mays*	Carbohydrate metabolism/Glycolysis
**36**	7	42%		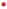	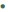	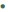	<0.0001	Prohibitin3	30580/7.00	gi|162462359/*Zea mays*	Cell growth
**45**	4	22%		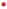	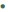	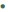	<0.0001	Prohibitin 2	30702/6.55	gi|162462211/*Zea mays*	Cell growth
**358**	26	55%			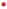	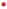	0.0018	Protein disulfide isomerase	56838/5.01	gi|162461063/*Zea mays*	Cell redox homeostasis
**29**	4	40%		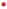	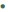	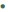	<0.0001	Actin depolymerizing factor	15890/5.46	gi|162459533/*Zea mays*	Cytoskeleton
**170**	5	40%		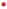	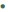	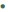	<0.0001	Actin depolymerizing factor	15890/5.46	gi|162459533/*Zea mays*	Cytoskeleton
**252**	11	51%			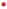		0.0003	Actin	41699/5.24	gi|53759189/*Saccharum officinarum*	Cytoskeleton
**340**	7	21%			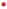	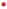	0.0033	Hypothetical protein LOC100191561/Actin	41699/5.24	gi|212274479/*Zea mays*	Cytoskeleton
**398**	7	40%		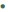			0.0409	Hypothetical protein LOC100193683/Proteasome subunit alpha type 2	25848/5.53	gi|212720956/*Zea mays*	Defense response to bacterium
**382**	2	8%		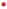		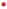	0.0145	Unknown/Ankyrin repeat domain-containing protein 2	36227/4.50	gi|194707992/*Zea mays*	Defense response to bacterium, incompatible interaction
**61**	15	91%		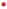	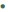	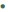	<0.0001	Late embryogenesis abundant protein Lea14-A	16078/8.05	gi|195658529/*Zea mays*	Defense response to dessiccation
**104**	3	14%			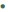	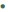	0.0007	Unknown/Dessication-related protein	34010/4.82	gi|194708240/*Zea mays*	Defense response to dessiccation
**1**	3	16%			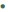	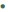	<0.0001	Late embryogenesis abundant protein, group 3	18588/7.85	gi|195605580/*Zea mays*	Embryo development ending in seed dormancy
**363**	9	44%				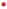	0.009	Unknown/APx1 - Cytosolic Ascorbate Peroxidase	27368/5.65	gi|195654277/*Zea mays*	Embryo development ending in seed dormancy
**385**	10	22%			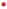		0.0314	Unknown/Vacuolar ATP synthase catalytic subunit A	68376/5.30	gi|195658441/*Zea mays*	Embryo development ending in seed dormancy
**387**	10	48%	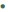	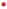	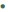	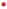	0.0012	Unknown/APx1 - Cytosolic Ascorbate Peroxidase	27368/5.65	gi|195654277/*Zea mays*	Embryo development ending in seed dormancy
**403**	7	36%			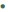		0.0189	Unknown/APx2 - Cytosolic Ascorbate Peroxidase	27211/5.28	gi|194707280/*Zea mays*	Embryo development ending in seed dormancy
**328**	4	8%			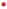		0.0273	Unknown/2-isopropylmalate synthase B	67138/7.02	gi|195604800/*Zea mays*	Glucosinolate biosynthesis process
**339**	17	30%				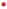	0.0463	Unknown/2-isopropylmalate synthase B	67138/7.02	gi|195604800/*Zea mays*	Glucosinolate biosynthesis process
**185**	2	3%			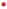	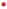	0.0003	Putative aconitate hydratase	98021/5.67	gi|75225211/*Oryza sativa*	Glyoxylate and dicarboxylate metabolism
**186**	3	6%			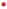	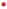	0.0009	Putative aconitate hydratase	98021/5.67	gi|75225211/*Oryza sativa*	Glyoxylate and dicarboxylate metabolism
**147**	5	16%			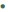	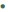	<0.0001	Catalase isozyme 1	56841/7.40	gi|115679/*Zea mays*	Hydrogen peroxide
**399**	3	7%			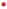		0.0451	Non-photosynthetic NADP-malic enzyme	70622/6.46	gi|37147841/*Zea mays*	Malate metabolic process
**9**	2	17%		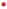	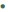	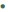	<0.0001	Unknown/Splicing factor	19898/11.53	gi|194695412/*Zea mays*	Nucleic acid binding
**266**	2	10%		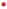	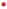		0.0093	Glycine-rich RNA binding protein	15908/5.22	gi|20257707/*Zea mays*	Nucleic acid binding
**282**	2	9%			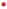	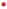	0.0006	Unknown/Plasminogen activator inhibitor 1 RNA-binding protein	40439/5.72	gi|194701098/*Zea mays*	Nucleic acid binding
**111**	3	7%	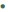	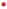	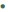	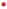	0.0001	Nucleoside diphosphate kinase 1	16835/6.30	gi|50096951/*Oryza sativa*	Nucleotide metabolism
**319**	4	19%		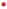		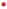	0.0144	Nucleoside diphosphate kinase 1	16835/6.30	gi|50096951/*Oryza sativa*	Nucleotide metabolism
**69**	16	54%			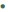	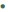	<0.0001	Unknown/Glucose and ribitol dehydrogenase homolog	32924/5.78	gi|194699516/*Zea mays*	Oxidation-reduction process
**334**	6	29%			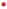		0.0023	Carbonyl reductase 1	32662/6.16	gi|195650645/*Zea mays*	Oxidation-reduction process
**335**	3	20%		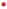	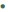	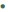	<0.0001	Unknown/Peroxiredoxin	17312/4.85	gi|194698866/*Zea mays*	Oxidation-reduction process
**43**	17	27%			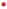	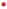	<0.0001	C4-specific pyruvate orthophosphate dikinase	102343/5.50	gi|31322754/*Miscanthus x giganteus*	Photosynthesis
**52**	34	51%			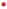	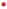	<0.0001	Chain A, Pyruvate Phosphate Dikinase	95132/5.27	gi|62738111/*Zea mays*	Photosynthesis
**38**	28	43%			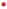	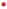	<0.0001	Pyruvate orthophosphate dikinase	102444/5.71	gi|168586/*Zea mays*	Photosynthesis
**53**	12	22%			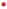	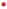	<0.0001	Pyruvate orthophosphate dikinase	102444/5.71	gi|168586/*Zea mays*	Photosynthesis
**74**	13	18%	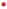		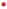	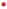	<0.0001	Pyruvate orthophosphate dikinase	102471/5.52	gi|6274486/*Saccharum officinarum*	Photosynthesis
**96**	13	19%			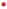	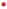	0.0002	Pyruvate orthophosphate dikinase	102471/5.52	gi|6274486/*Saccharum officinarum*	Photosynthesis
**107**	17	21%			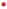	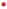	<0.0001	Pyruvate orthophosphate dikinase	102471/5.52	gi|6274486/*Saccharum officinarum*	Photosynthesis
**207**	2	10%			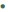		0.0047	QM protein	24903/10.27	gi|162458844/*Zea mays*	Photosynthesis
**276**	5	10%			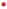	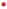	0.0014	Pyruvate, orthophosphate dikinase	102444/5.71	gi|168586/*Zea mays*	Photosynthesis
**108**	14	24%	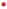		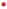		0.0006	Os02g0519900/Elongation factor 2	93961/5.85	gi|115446385/*Oryza sativa*	Protein biosynthesis
**139**	2	10%		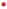	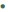	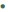	<0.0001	Translation initiation factor 5A	17486/5.61	gi|162458009/*Zea mays*	Protein biosynthesis
**174**	13	35%			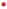	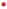	0.0003	Unknown/Eukariotic translation initiation factor 3 subunit 7	64846/5.51	gi|194704818/*Zea mays*	Protein biosynthesis
**178**	12	23%			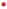	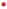	<0.0001	Os02g0519900/Elongation factor 2	93961/5.85	gi|115446385/*Oryza sativa*	Protein biosynthesis
**360**	12	38%	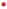		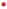		0.0041	Translational initiation factor eIF-4A	46952/5.37	gi|162458395/*Zea mays*	Protein biosynthesis
**286**	5	27%			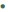		0.0055	Unknown/Proteasome subunit alpha type 5	25961/4.76	gi|195635461/*Zea mays*	Protein catabolic process
**66**	5	39%			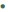	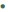	<0.0001	Unknown/Putative chaperonin 21 precursor	25739/8.49	gi|194688414/*Zea mays*	Protein folding
**265**	10	27%			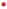	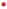	0.0005	Os02g0102900 /RuBisCO large subunit-binding protein	63759/5.77	gi|115443643/*Oryza sativa*	Protein folding
**284**	16	33%			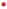		0.0145	Unknown/T-complex protein 1 subunit alpha	59158/5.78	gi|195636596/*Zea mays*	Protein folding
**287**	11	22%			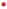	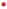	0.0211	Os06g0114000/Chaperonin 60 Beta	64046/5.60	gi|115466004/*Oryza sativa*	Protein folding
**318**	9	48%			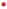	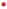	0.0003	Peptidyl-prolyl cis-trans isomerase	18337/8.91	gi|118104/*Zea mays*	Protein folding
**86**	13	22%	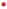		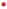		0.0029	OSJNBa0039C07.4/ATP dependent Clp protease ATP-binding subunit	98436/5.79	gi|38347158/*Oryza sativa*	Protein metabolic process
**148**	7	13%			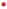	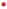	<0.0001	Os12g0230100/ATP dependent Clp protease	101954/6.62	gi|115487910/*Oryza sativa*	Protein metabolic process
**338**	3	34%		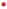		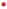	0.0100	Unknown/NADH ubiquinone oxidoreductase B22-like subunit	13346/8.01	gi|195605254/*Zea mays*	Respiratory chain
**214**	4	15%			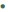	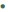	<0.0001	Glyoxalase I	32336/5.59	gi|162461576/*Zea mays*	Response to salt stress
**292**	13	25%	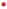		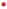		0.0416	Unknown/Ketol-acid reductoisomerase	62963/6.31	gi|194693902/*Zea mays*	Response to salt stress
**337**	11	27%			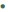		0.0084	Alanine aminotransferase 2	53000/6.23	gi|195625602/*Zea mays*	Response to salt stress
**357**	7	18%			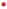		0.0293	Adenosine kinase	36009/5.23	gi|4582787/*Zea mays*	Response to salt stress
**12**	6	41%		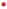	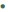	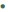	<0.0001	Unknown/Transcription factor homolog	17757/6.62	gi|194695608/*Zea mays*	Response to salt stress
**134**	13	19%			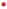	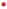	<0.0001	Putative aconitate hydratase 1	106913/6.63	gi|92429669/*Sorghum bicolor*	Response to salt stress
**189**	3	27%			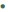	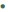	0.0034	Unknown/Mitochondrial F0 ATP synthase D chain	19915/5.19	gi|194701816/*Zea mays*	Response to salt stress
**192**	10	20%			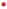		0.0014	Methionine synthase protein	83736/5.93	gi|18483235/*Sorghum bicolor*	Response to salt stress
**273**	7	44%		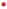	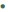		0.0003	Unknown/Superoxide dismutase 3	25571/7.11	gi|194689068/*Zea mays*	Response to salt stress
**395**	8	29%		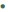	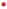		0.019	Hypothetical protein LOC100191638/Salt tolerance protein	35252/4.92	gi|212274681/*Zea mays*	Response to salt stress
**15**	3	6%			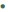	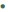	<0.0001	Vicilin-like embryo storage protein	66122/6.23	gi|22284/*Zea mays*	Seed storage
**21**	4	19%		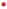	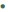		0.0004	Zein-alpha 19D1 precursor	26616/9.21	gi|162458484/*Zea mays*	Seed storage
**26**	3	6%		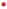	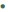	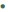	<0.0001	Vicilin-like embryo storage protein	66122/6.23	gi|22284/*Zea mays*	Seed storage
**44**	9	20%			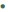	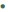	<0.0001	Vicilin-like embryo storage protein	66122/6.23	gi|22284/*Zea mays*	Seed storage
**51**	3	6%		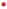	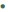		<0.0001	Vicilin-like embryo storage protein	66122/6.23	gi|22284/*Zea mays*	Seed storage
**188**	4	14%			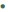	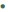	0.0031	Vicilin-like embryo storage protein	66122/6.23	gi|22284/*Zea mays*	Seed storage
**242**	7	19%		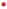	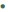		0.0010	Vicilin-like embryo storage protein	66122/6.23	gi|22284/*Zea mays*	Seed storage
**261**	1	10%				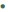	0.0229	Zein protein precursor	19448/8.05	gi|168664/*Zea mays*	Seed storage
**354**	4	6%			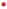	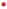	0.0187	Vicilin-like embryo storage protein	66122/6.23	gi|22284/*Zea mays*	Seed storage
**64**	1	23%		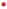	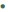		0.0003	z1B alpha zein protein	16047/8.00	gi|157780962/*Zea mays*	Seed storage (nutrient reservoir activity)
**179**	10	34%	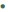		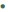		0.0135	Legumin 1	52798/6.20	gi|162460908/*Zea mays*	Seed storage (nutrient reservoir activity)
**50**	11	51%			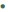	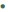	<0.0001	Unknown/Chitinase	29193/8.44	gi|194702870/*Zea mays*	Somatic embryogenesis/fruit development
**210**	11	22%			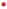		0.0232	Granule-bound starch synthase precursor	66567/6.59	gi|33321047/*Zea mays*	Starch metabolic process
**342**	10	22%			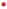	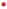	0.0036	Granule-bound starch synthase precursor	66567/6.59	gi|33321047/*Zea mays*	Starch metabolic process
**83**	10	38%			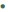	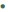	0.0002	Unknown/Stress responsive protein	38371/6.30	gi|194707628/*Zea mays*	Stress response
**4**	6	30%		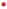	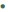	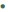	<0.0001	22.0 kDa class IV heat shock protein precursor	22872/6.01	gi|195644560/*Zea mays*	Stress response
**13**	3	26%			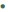	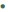	<0.0001	Heat shock protein 17.2	17152/5.54	gi|162459222/*Zea mays*	Stress response
**49**	4	16%		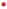	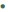		0.0018	Activator of 90 kDa heat shock protein ATPase	38577/5.33	gi|195651993/*Zea mays*	Stress response
**55**	10	21%	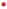		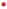	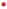	<0.0001	Heat shock 70 kDa protein	72704/5.62	gi|195649437/*Zea mays*	Stress response
**59**	14	54%	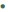	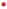	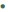	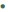	<0.0001	Unknown/Stress responsive protein	38371/6.30	gi|194707628/*Zea mays*	Stress response
**87**	2	3%			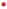		0.0059	Putative heat shock protein	82531/5.43	gi|37718900/*Oryza sativa*	Stress response
**149**	15	26%			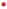	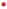	0.0027	Heat-shock protein 101	101069/5.85	gi|162458166/*Zea mays*	Stress response
**154**	10	21%			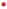		0.0036	Unknown/NADH ubiquinone oxidoreductase 75 kDa subunit	80628/6.10	gi|194688928/*Zea mays*	Stress response
**172**	8	31%			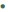	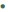	<0.0001	Lactoylglutathione lyase	35140/6.62	gi|195639070/*Zea mays*	Stress response
**197**	8	42%			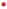	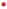	<0.0001	Unknown/Stress responsive protein	37857/6.70	gi|194703432/*Zea mays*	Stress response
**213**	4	42%		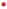			0.0002	Hypothetical protein Z477F24.14/Lactoylglutathione lyase	15597/4.94	gi|48374986/*Zea mays*	Stress response
**272**	6	41%			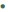		0.0012	Hypothetical protein LOC100191552/Heat shock protein 17.9	17869/6.86	gi|212276212/*Zea mays*	Stress response
**312**	11	41%			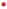	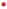	<0.0001	Unknown/Stress responsive protein	37857/6.70	gi|194703432/*Zea mays*	Stress response
**356**	18	29%				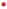	0.0149	Unknown/Stromal 70 kDa heat shock-related protein	74625/5.08	gi|195657157/*Zea mays*	Stress response
**376**	15	21%			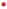	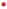	0.0134	Unknown/Heat shock 70 kDa protein	72704/5.62	gi|195649437/*Zea mays*	Stress response
**352**	7	19%			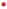	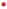	<0.0001	Unknown/Putative aminotransferase	49566/6.55	gi|195634861/*Zea mays*	Systemic acquired resistance
**336**	4	32%			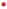	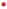	0.0030	Unknown/40S ribosomal protein S7	22198/9.76	gi|195605060/*Zea mays*	Translation
**268**	6	32%			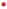	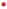	<0.0001	Malate dehydrogenase 5	35567/5.77	gi|162464321/*Zea mays*	Tricarboxylic acid cycle
**394**	5	12%	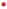		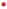		0.0150	Succinate dehydrogenase flavoprotein subunit	67941/6.08	gi|195647178/*Zea mays*	Tricarboxylic acid cycle
**5**	3	8%		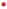	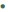	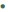	<0.0001	Fasciclin-like arabinogalactan protein 8 precursor	44699/6.56	gi|195607426/*Zea mays*	Unknown
**78**	8	49%			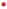	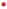	0.0013	Unknown/Carbonyl reductase	20867/6.84	gi|194701990/*Zea mays*	Unknown
**206**	5	53%	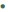			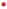	0.0009	Chain A, Bifunctional Hageman Factor AMYLASE INHIBITOR FROM MAIZE	13570/6.51	gi|157830250/*Zea mays*	Unknown
**329**	2	12%			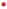	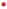	0.0066	Unknown/DREPP4 protein	22630/4.89	gi|194690236/*Zea mays*	Unknown
**343**	5	16%			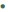		0.0098	Unknown/Seed protein	26645/7.11	gi|195626982/Zea mays	Unknown
**362**	3	13%			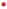	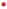	<0.0001	Unknown/Malonyl-CoA:ACP transacylase 1-3	38593/5.64	gi|195638470/*Zea mays*	Unknown
**392**	11	24%			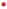	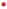	0.0400	Unknown/UDP-glucose pyrophosphorylase	52056/5.30	gi|194688950/*Zea mays*	Unknown
**17**	7	38%			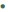		0.0013	Rab28	27693/4.90	gi|22460/*Zea mays*	Unknown
**6**						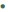	0.0141	ND			
**11**				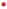	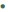	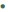	<0.0001	ND			
**40**					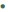	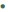	<0.0001	ND			
**175**					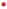	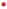	0.0003	ND			
**200**					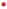	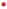	0.0045	ND			
**203**			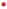		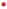		<0.0001	ND			
**257**					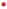	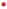	0.0001	ND			
**262**					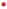	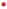	<0.0001	ND			
**267**				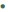		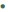	0.0071	ND			
**389**					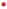	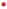	0.0034	ND			
