# Supplementary material for: Arbuscular mycorrhizal symbiosis affects the grain proteome of Zea mays: a field study
Source: Sci Rep. 2016 May 24;6:26439. doi: 10.1038/srep26439 (PMC4877657; doi:10.1038/srep26439)
Supplement: Supplementary Information [file srep26439-s1.pdf]

Supporting Information for the paper

Arbuscular mycorrhizal symbiosis affects the grain proteome of *Zea mays* : a field study

Elisa Bona[1] ‡, *Alessio Scarafoni*[2] ‡, *Francesco Marsano*[1] , *Lara Boatti*[1] , *Andrea Copetta*[1] , *Nadia Massa*[1] , *Elisa Gamalero*[1] , *Giovanni D'Agostino*[3] , *Patrizia Cesaro*[1] , *Maria Cavaletto*[1] , *Graziella Berta*[1]

[1] Dipartimento di Scienze ed Innovazione Tecnologica, Università del Piemonte Orientale Amedeo Avogadro, Viale Teresa Michel, 11, 15121 Alessandria, Italy

[2] Dipartimento di Scienze per gli Alimenti, Università degli Studi di Milano, la Nutrizione e l'Ambiente Via Celoria, 2, 20133 Milano, Italy

[3] Mybasol srl, Via Gentilini 3, 15121 Alessandria, Italy

Table of contents of the supplementary materials:

Table S1: Optical densities
Table S2: Protein identification with MS details
Table S3: BLAST results
Table S4: Kegg table

Table S1: Mean and standard error of OD values

Spot	CTRL 20 DAF	MIC 20 DAF	CTRL 60 DAF	MIC 60 DAF	P value
1	292661 ± 54599 a	562449 ± 118416 a	3627060 ± 767676 b	3299949 ± 454083 b	< 0.0001
4	304800 ± 56828 a	341838 ± 74834 a	2645736 ± 477647 b	1491591 ± 516449 c	< 0.0001
5	336327 ± 71390 a	342940 ± 78906 a	2869554 ± 365472 b	1702820 ± 360972 c	< 0.0001
6	296765 ± 186900 a	145037 ± 30288 a	833004 ± 169933 ab	1123322 ± 298901 b	0.0141
9	194966 ± 37169 a	219487 ± 49866 a	1309516 ± 66785 b	685084 ± 99366 c	< 0.0001
11	2166035 ± 162228 a	3077271 ± 437265 a	13780683 ± 2097689 b	6889487 ± 1368484 c	< 0.0001
12	561312 ± 72155 a	562799 ± 67423 a	3459684 ± 362050 b	2073852 ± 333446 c	< 0.0001
13	272822 ± 41887 a	319991 ± 21966 a	1624400 ± 133679 b	1471164 ± 265424 b	< 0.0001
15	207937 ± 47693 a	275538 ± 46863 a	1214133 ± 139706 b	977392 ± 78378 b	< 0.0001
17	348372 ± 70467 a	857565 ± 388906 ac	1858509 ± 326958 b	1704489 ± 346085 bc	0.0013
21	370952 ± 74025 a	373034 ± 68375 a	1725242 ± 398618 b	857612 ± 211785 a	0.0004
24	3241598 ± 256467 a	3393424 ± 124657 a	746713 ± 89879 b	1026300 ± 169659 b	< 0.0001
26	155209 ± 26435 a	121302 ± 19485 a	544086 ± 65108 b	295134 ± 49212 c	< 0.0001
29	394596 ± 106620 a	634157 ± 107764 a	1764440 ± 141536 b	1238376 ± 176710 c	< 0.0001
36	933831 ± 70180 a	1203535 ± 131782 a	3874600 ± 173299 b	2912117 ± 454085 c	< 0.0001
38	550998 ± 60998 a	607913 ± 28781 a	147673 ± 19489 b	222419 ± 37874 b	< 0.0001
40	308163 ± 32945 a	291210 ± 29969 a	1197465 ± 167812 b	830361 ± 221912 b	< 0.0001
43	4641102 ± 276155 a	4095495 ± 345413 a	1146444 ± 197761 b	1472776 ± 308574 b	< 0.0001
44	152804 ± 15938 a	190996 ± 22481 a	614466 ± 99973 b	444278 ± 78446 b	< 0.0001
45	273779 ± 18534 a	300252 ± 23316 a	1091823 ± 87101 b	688550 ± 169503 c	< 0.0001
49	161404 ± 14404 a	120664 ± 12703 a	468429 ± 103378 b	248099 ± 82715 a	0.0018
50	1108304 ± 61178 a	1142564 ± 58229 a	3899768 ± 360869 b	4230967 ± 716054 b	< 0.0001
51	267689 ± 65554 a	307994 ± 10027 a	1018097 ± 146947 b	524941 ± 103698 a	< 0.0001
52	2302981 ± 165055 a	2698525 ± 178321 a	729953 ± 90651 b	1014847 ± 174524 b	< 0.0001
53	742210 ± 36863 a	841628 ± 51041 a	230263 ± 35980 b	317682 ± 83634 b	< 0.0001
54	163832 ± 9202 a	173295 ± 13556 a	47713 ± 5345 b	64109 ± 11891 b	< 0.0001
55	309979 ± 26174 a	238740 ± 7463 b	89622 ± 10207 c	85594 ± 19489 c	< 0.0001
59	434552 ± 46925 a	807522 ± 92816 b	1532131 ± 123694 c	1184802 ± 118661 d	< 0.0001
61	368509 ± 28756 a	404097 ± 46525 a	1282405 ± 97689 b	900335 ± 229667 c	< 0.0001
64	2019255 ± 214619 a	1328755 ± 183805 a	4550512 ± 808538 b	2849692 ± 396429 a	0.0003
66	232026 ± 16679 a	350514 ± 26788 a	788418 ± 71843 b	644341 ± 123619 b	< 0.0001
67	461888 ± 48637 a	375100 ± 47827 a	149666 ± 28870 b	136868 ± 26853 b	< 0.0001
69	364789 ± 53698 a	441350 ± 90426 a	1227188 ± 97827 b	1029261 ± 198088 b	< 0.0001
74	1795126 ± 105538 a	1447524 ± 127579 b	558699 ± 89228 c	606189 ± 136616 c	< 0.0001
78	397945 ± 57200 a	485856 ± 62658 a	155352 ± 18945 b	258428 ± 36328 b	0.0013
83	1023912 ± 61455 a	1255562 ± 16297 a	3133373 ± 322486 b	2878419 ± 788083 b	0.0002
86	110482 ± 14226 a	71163 ± 15995 b	36559 ± 4933 b	54875 ± 11721 b	0.0029
87	370660 ± 43146 a	372556 ± 86591 a	123490 ± 16918 b	205308 ± 42280 b	0.0059
96	403243 ± 46744 a	446754 ± 66097 a	151044 ± 26110 b	154734 ± 40756 b	0.0002
104	119608 ± 23874 a	124909 ± 10643 a	338916 ± 53546 b	273973 ± 62276 b	0.0007
107	1131998 ± 74893 a	974018 ± 68509 a	400815 ± 79489 b	461242 ± 114265 b	< 0.0001
108	1115443 ± 132844 a	787307 ± 64558 b	395835 ± 59561 c	641377 ± 102310 bc	0.0006
111	663592 ± 49889 a	1593104 ± 197234 b	1337119 ± 216933 b	568848 ± 125138 a	0.0001
112	3750653 ± 341400 a	3663973 ± 444444 a	1339870 ± 60520 b	1887149 ± 545277 b	0.0002
114	1363624 ± 95374 a	1499923 ± 143662 a	3784404 ± 247938 b	2897724 ± 386788 c	< 0.0001
134	429011 ± 39399 a	390688 ± 40474 a	163960 ± 21937 b	199903 ± 35799 b	< 0.0001
136	442390 ± 46373 a	355131 ± 43472 b	170040 ± 18638 b	233173 ± 23354 ab	0.0002
139	1577238 ± 134033 a	1993223 ± 134592 a	4079915 ± 191002 b	3235326 ± 364284 c	< 0.0001
145	1351913 ± 123926 a	1251340 ± 113636 a	614061 ± 112338 b	528891 ± 69204 b	< 0.0001
147	1161984 ± 70255 a	1490471 ± 84959 a	2705129 ± 236376 b	2953863 ± 276457 b	< 0.0001
148	949397 ± 50751 a	974950 ± 105257 a	383768 ± 41872 b	473563 ± 88478 b	< 0.0001
149	389226 ± 41525 a	438030 ± 39949 a	173075 ± 70338 b	201844 ± 50226 b	0.0027
154	198949 ± 14081 a	166518 ± 11508 ab	79313 ± 10942 c	123634 ± 48333 bc	0.0036
163	2886567 ± 284333 a	2476274 ± 301299 a	1339508 ± 150741 b	1186714 ± 175398 b	0.0002

Spot	CTRL 20 DAF	MIC 20 DAF	CTRL 60 DAF	MIC 60 DAF	P value
**164**	704873 ± 48686 a	606021 ± 41720 a	290076 ± 45738 b	341165 ± 57426 b	< 0.0001
**170**	2374364 ± 127040 a	2386333 ± 174902 a	5666220 ± 89521 b	3656980 ± 474697 c	< 0.0001
**172**	2397511 ± 143871 a	2532192 ± 222103 a	5710032 ± 117262 b	4988936 ± 888098 b	< 0.0001
**174**	289042 ± 30516 a	239502 ± 20981 a	126498 ± 18185 b	121873 ± 29078 b	0.0003
**175**	778911 ± 81029 a	792954 ± 85261 a	334749 ± 52287 b	404788 ± 67300 b	0.0003
**178**	2905328 ± 246807 a	2610831 ± 141032 a	1234569 ± 159373 b	1573865 ± 289638 b	< 0.0001
**179**	472107 ± 35704 a	493813 ± 61195 b	772244 ± 117673 b	1110235 ± 313011 b	0.0135
**185**	1470928 ± 155344 a	1333468 ± 87108 a	633794 ± 97693 b	808287 ± 123066 b	0.0003
**186**	436426 ± 42812 a	404823 ± 34436 a	188095 ± 52130 b	230339 ± 41599 b	0.0009
**188**	373371 ± 35773 a	404209 ± 95082 a	862944 ± 47776 b	756008 ± 220321 b	0.0031
**189**	348920 ± 11379 a	444328 ± 32785 a	804588 ± 54023 b	740155 ± 231179 b	0.0034
**192**	140386 ± 13735 a	117793 ± 16468 ac	61253 ± 7424 b	79328 ± 13495 bc	0.0014
**196**	3465054 ± 326986 a	3281626 ± 386558 a	1687751 ± 211879 b	1528587 ± 351676 b	0.0004
**197**	5064455 ± 350655 a	5480648 ± 346487 a	2877036 ± 137544 b	2420645 ± 234263 b	< 0.0001
**200**	120307 ± 15785 a	107529 ± 13003 a	53630 ± 4976 b	60487 ± 15178 b	0.0045
**203**	282036 ± 16324 a	204513 ± 16324 b	126097 ± 15944 c	150837 ± 33472 bc	< 0.0001
**205**	5974211 ± 633755 a	6125100 ± 772759 a	4216185 ± 422345 ab	2753298 ± 501677 b	0.0045
**206**	7196019 ± 511738 a	11461303 ± 796393 b	7195889 ± 1309941 a	5163715 ± 1028383 a	0.0009
**207**	705021 ± 64820 a	930153 ± 96402 ac	1555821 ± 242856 b	1311478 ± 275776 bc	0.0047
**210**	1065028 ± 170473 a	921979 ± 104683 ac	482937 ± 87802 b	613489 ± 96383 bc	0.0232
**213**	435206 ± 28669 ab	478843 ± 30001 b	868903 ± 123621 a	395534 ± 56989 b	0.0002
**214**	1693227 ± 80647 a	2096782 ± 166230 a	3712281 ± 369239 b	3184331 ± 202153 b	< 0.0001
**219**	4590871 ± 454818 a	9236453 ± 704333 b	7031103 ± 522149 c	4245088 ± 1130566 a	< 0.0001
**242**	585372 ± 59856 a	602125 ± 84577 a	1230279 ± 100886 b	860593 ± 211819 a	0.0010
**245**	260015 ± 25381 a	233524 ± 16003 b	124101 ± 15286 b	215074 ± 62295 ab	0.0272
**249**	1468706 ± 97985 a	1440936 ± 86449 a	780307 ± 88250 b	708567 ± 92025 b	< 0.0001
**251**	1477937 ± 108162 a	1485356 ± 155635 a	802011 ± 102260 b	718455 ± 169609 b	0.0003
**252**	1371074 ± 134720 a	1116357 ± 81984 ac	664194 ± 69933 b	779804 ± 54094 bc	0.0003
**257**	954789 ± 69464 a	858214 ± 22149 a	465918 ± 20662 b	570614 ± 132193 b	0.0001
**261**	10764499 ± 920083 a	15461941 ± 1196326	21451082 ± 1471607 a	21879540 ± 6909500 a	0.0229
**262**	291331 ± 24768 a	300829 ± 20971 a	148101 ± 11338 b	199676 ± 15805 b	< 0.0001
**265**	416644 ± 26979 a	326269 ± 51580 a	211593 ± 10190 b	205497 ± 45846 b	0.0005
**266**	4683473 ± 310069 a	4670376 ± 721148 a	2605788 ± 236375 b	5279826 ± 1332608 a	0.0093
**267**	86294 ± 6348 a	71756 ± 12910 a	61082 ± 7621 a	123668 ± 18900 b	0.0071
**268**	3153725 ± 190354 a	2754594 ± 190432 a	1588670 ± 172361 b	1564296 ± 184316 b	< 0.0001
**272**	8292640 ± 792787 a	10112551 ± 1690137	16595350 ± 1224402 b	13728292 ± 2254136 b	0.0012
**273**	1370837 ± 92207 a	1425826 ± 218344 a	2741274 ± 176484 b	1773726 ± 389855 a	0.0003
**274**	5109338 ± 736237 a	6152624 ± 691289 a	3526786 ± 252057 a	3077593 ± 360727 b	0.0135
**276**	731671 ± 44813 ac	810146 ± 79122 a	406488 ± 69737 b	549131 ± 83783 bc	0.0014
**278**	4037641 ± 428080 a	3819762 ± 485115 ac	2569442 ± 190289 bc	2050015 ± 397808 b	0.0070
**282**	1107939 ± 107482 a	1131492 ± 135145 a	577398 ± 36080 b	607413 ± 64928 b	0.0006
**284**	130947 ± 11152 a	149568 ± 19282 ac	76400 ± 11181 b	90854 ± 24220 bc	0.0145
**286**	1207464 ± 84480 ac	1318662 ± 56654 bc	2355674 ± 245819 b	2034080 ± 534436 b	0.0055
**287**	221106 ± 27356 a	198242 ± 41077 ac	137380 ± 22355 bc	113518 ± 13435 b	0.0211
**292**	451492 ± 50616 a	334238 ± 27979 b	273921 ± 65340 b	234505 ± 71681 b	0.0416
**305**	1505774 ± 112564 a	1245208 ± 117532 a	843057 ± 83393 b	809530 ± 77786 b	0.0002
**309**	954216 ± 78617 a	1162560 ± 198257 a	1762168 ± 235464 b	1313952 ± 159596 ab	0.0112
**311**	245622 ± 11016 a	290383 ± 24644 a	185768 ± 29976 b	157835 ± 17909 b	0.0013
**312**	1669030 ± 124136 a	1581648 ± 61180 a	928620 ± 58696 b	907514 ± 172369 b	< 0.0001
**318**	9655026 ± 590144 a	9000501 ± 1050796 a	6062059 ± 531907 b	5299221 ± 368878 b	0.0003
**319**	2421507 ± 173074 a	2577716 ± 234793 a	2758056 ± 267430 a	1515703 ± 324448 b	0.0144
**321**	3018735 ± 355312 a	3268785 ± 246472 a	2049688 ± 96708 b	1807263 ± 321808 b	0.0079
**326**	538398 ± 30628 a	500793 ± 8653 a	899862 ± 54950 b	883310 ± 120946 b	< 0.0001
**328**	140951 ± 8850 a	107224 ± 20245 ab	78662 ± 12072 b	108171 ± 17821 ab	0.0273
**329**	205726 ± 14939 a	215063 ± 29532 a	120100 ± 10023 b	147156 ± 22273 b	0.0066

Spot	CTRL 20 DAF	MIC 20 DAF	CTRL 60 DAF	MIC 60 DAF	P value
**334**	393829 ± 19015 a	347260 ± 38870 ac	220844 ± 45431 b	260857 ± 22890 bc	0.0023
**335**	1470042 ± 67144 a	1640559 ± 87300 a	2619585 ± 85639 b	2121303 ± 195350 c	< 0.0001
**336**	1172199 ± 84796 a	1016019 ± 173256 a	677563 ± 51853 b	657902 ± 46499 b	0.003
**337**	1537409 ± 118235 a	1833000 ± 199232 ac	2738630 ± 189889 b	2672679 ± 611348 bc	0.0084
**338**	1420593 ± 100586 a	1669860 ± 102691 a	1365791 ± 96387 a	941730 ± 215321 b	0.0100
**339**	695598 ± 57602 a	711962 ± 66770 a	581440 ± 104211 ab	402548 ± 74105 b	0.0463
**340**	824465 ± 84108 a	776537 ± 71375 a	484410 ± 57085 b	467964 ± 56216 b	0.0033
**342**	1651679 ± 130314 a	1574427 ± 152802 a	939796 ± 155246 b	961375 ± 204777 b	0.0036
**343**	714897 ± 39251 a	926082 ± 44133 ab	1252860 ± 69988 b	1082730 ± 265231 b	0.0098
**352**	722029 ± 40803 a	817472 ± 18977 a	471526 ± 28648 b	497296 ± 45991 b	< 0.0001
**354**	681010 ± 57284 a	677509 ± 100454 ac	469657 ± 60513 bc	399633 ± 43915 b	0.0187
**356**	2726511 ± 190240 ac	3095502 ± 134509 c	2142487 ± 307941 ab	1835500 ± 410623 b	0.0149
**357**	579610 ± 52972 a	499658 ± 53464 ab	346002 ± 22019 b	430259 ± 79803 ab	0.0293
**358**	4249915 ± 376387 a	4485480 ± 205328 a	2684384 ± 199172 b	2857962 ± 463959 b	0.0018
**360**	898290 ± 78878 a	556026 ± 31802 b	539237 ± 31515 b	668473 ± 118771 b	0.0041
**362**	373630 ± 17127 a	372649 ± 28440 a	252222 ± 18226 b	224598 ± 26994 b	< 0.0001
**363**	4950798 ± 286472 a	6037738 ± 457101 a	4574362 ± 550967 ab	3663646 ± 357236 b	0.0090
**368**	3837348 ± 224071 a	3706022 ± 348268 a	2531349 ± 293991 b	2370699 ± 605765 b	0.0100
**376**	589572 ± 46731 a	562071 ± 35981 ac	4191462 ± 46684 bc	371759 ± 68372 b	0.0134
**380**	1127242 ± 82625 a	973488 ± 29489 ac	758094 ± 45888 bc	720388 ± 104611 b	0.0018
**381**	1360137 ± 143974 a	1126376 ± 72156 ac	874736 ± 136704 bc	907375 ± 91142 bc	0.0357
**382**	605392 ± 24053 a	718542 ± 76192 a	640954 ± 59487 a	464477 ± 53600 b	0.0145
**385**	308583 ± 23528 a	248779 ± 17050 ab	201774 ± 28683 b	240044 ± 30134 ab	0.0314
**387**	3957094 ± 276916 a	4736288 ± 282692 b	5608461 ± 217492 b	3679462 ± 447742 a	0.0012
**388**	1006981 ± 99977 ac	854945 ± 62809 bc	686288 ± 54736 b	661435 ± 111263 b	0.0344
**389**	558495 ± 18321 a	570784 ± 54075 a	375260 ± 43231 b	401249 ± 59467 b	0.0034
**392**	2217315 ± 213197 a	2316854 ± 216865 a	1594200 ± 109907 b	1529266 ± 304915 b	0.0400
**394**	294541 ± 21139 a	197802 ± 21953 b	196041 ± 28830 b	208397 ± 29467 b	0.0150
**395**	796178 ± 47269 a	790918 ± 46894 a	564425 ± 31033 b	801274 ± 153309 a	0.019
**398**	1209052 ± 92794 ab	1445103 ± 63368 ab	1496737 ± 40782 a	1724222 ± 248651 b	0.0409
**399**	6473077 ± 547508 a	6497660 ± 593340 ac	4620053 ± 423625 b	4769956 ± 711839 bc	0.0451
**403**	2146040 ± 172232 a	2587867 ± 55329 ac	2799301 ± 81630 b	2894793 ± 312548 bc	0.0189

Table S1: Mean and standard error of OD values; P-value of ANOVA analysis performed for each spot. ANOVA followed by Fisher's probable least-squares difference test used a cut-off significance at p=0.05.
Different letters indicate significantly different values based on one-way ANOVA (P<0.05).

Table S2: Spot, Precursor ion m/z, Precursor ion mass, Ion charge, Collision Energy, Number of spectra for identification, Delta MS (ppm), Sequence, Modification, Peptide score*, Protein, Mr (kDa) / pI Theor, Mr (kDa) / pI Observed, AC number (gi NCBI) and reference organism

Spot	Precursor ion *m/z*	Precursor ion mass	Ion charge	Collision Energy	Number of spectra for identification	Delta MS (ppm)
**1**	499.7448	997.4716	2	27.7343	3	0.0035
	634.3162	1900.0014	3	33.0590		-0.0746
	672.8431	1343.6717	2	38.3799		-0.0328
**4**	779.7021	2336.1219	3	41.7821	6	-0.0375
	849.1069	2544.3184	3	45.9464		-0,0196
	881.9135	1761.8356	2	51.2377		-0,0232
	901.1237	2700.4195	3	49.0674		-0,0703
	958.4847	1914.9687	2	55.9468		-0,0139
	1121.5524	2241.1488	2	65.9755		-0,0586
**5**	685.0386	2052.0779	3	36.1023	3	0,0161
	765.7438	2294.2158	3	40.9446		-0,0063
	792.8974	1583.8056	2	45.7632		-0,0254
**6**						
**9**	560.9855	1679.8420	3	28.6591	2	0,0928
	651.7146	1952.0004	3	34.1029		0,1215
**11**						
**12**	691.4135	2071.1062	3	36.4848	6	0,1124
	787.8108	2360.2699	3	42.2686		0,1405
	830.4928	2488.3649	3	44.8296		0,0918
	839.8314	2516.3710	3	45.3899		0,1014
	972.5544	1943.0113	2	56.8121		0,0829
	999.9303	2996.6223	3	54.9958		0,1469
**13**	533.9739	1598.8093	3	27.0384	3	0,0904
	580.0078	1736.9097	3	29.8005		0,0919
	984.5322	1966.9596	2	57.5487		0,0902
**15**	553.6752	1657.9151	3	28.2205	3	0,0887
	637.8936	1273.7030	2	36.2305		0,0697
	694.0883	2079.1266	3	36.6453		0,1165
**17**	636.8839	1271.6834	2	36.1684	7	0.0699
	731.9008	2923.4207	4	37.1821		0.1535
	801.0901	2400.1340	3	43.0654		0.1145
	837.8213	2510.3228	3	45.2693		0.1192
	911.5125	1820.9785	2	53.0580		0.0320
	1027.5902	2053.1659	2	60.1968		0.0968

Spot	Precursor ion *m/z*	Precursor ion mass	Ion charge	Collision Energy	Number of spectra for identification	Delta MS (ppm)
	1041.5874	2081.0753	2	61.0576		0.0849
21	592.8823	1183.6924	2	33.4623	3	0.0575
	652.3962	1302.6244	2	37.1224		0.1534
	823.8148	2468.3063	3	44.4289		0.1164
	952.9200	2855.5909	3	52.1752		0.1473
24	646.8553	1291.6561	2	36.7816	5	0.0399
	746.6922	2982.6138	4	38.0548		0.1259
	766.7999	2297.2744	3	41.0080		0.1034
	844.4931	2530.3288	3	45.6696		0.1287
	1008.0950	2014.0921	2	58.9978		0.0833
26	637.8842	1273.7030	2	36.2299	3	0.0507
	694.0809	2079.1266	3	36.6449		0.0943
	830.0070	1657.9151	2	48.0454		0.0844
29	706.4118	1410.7547	2	40.4443	4	0.0542
	723.0436	2166.0269	3	38.3826		0.0820
	934.8457	2801.3930	3	51.0907		0.1222
	962.4739	1922.8938	2	56.1921		0.0393
36	584.8701	1167.6863	2	32.9695	7	0.0394
	596.3448	1190.6329	2	33.6752		0.0421
	634.3784	2533.3765	4	31.4283		0.1079
	643.6361	2570.3931	4	31.9745		0.1224
	693.9255	1385.7878	2	39.6764		0.0486
	880.0154	1757.9523	2	51.1209		0.0640
	924.8406	2771.4018	3	50.4904		0.0981
38	550.3171	1647.8654	3	28.0190	28	0.0640
	554.3278	1659.8800	3	28.2597		0.0816
	574.3507	1719.9632	3	29.4610		0.0670
	595.3571	1782.9476	3	30.7214		0.1019
	636.8744	1271.6874	2	36.1678		0.0469
	639.8563	1277.6438	2	36.3512		0.0542
	642.3441	2565.2322	4	31.8983		0.1150
	651.8819	1301.6835	2	37.0907		0.0658
	680.7096	2039.0146	3	35.8426		0.0923
	719.3786	2155.0215	3	38.1627		0.0926
	720.4191	2158.1422	3	38.2251		0.0932
	726.7532	2177.1481	3	38.6052		0.0898
	750.9470	1499.8208	2	43.1832		0.0586
	771.4340	1540.7919	2	44.4432		0.0615
	778.9420	3111.6030	4	39.9576		0.1359

Spot	Precursor ion *m*/*z*	Precursor ion mass	Ion charge	Collision Energy	Number of spectra for identification	Delta MS (ppm)
	804.4366	1606.8025	2	46.4729		0.0561
	827.4428	1652.8055	2	47.8877		0.0655
	828.1893	3308.5878	4	42.8632		0.1402
	830.1150	2487.2104	3	44.8069		0.1127
	866.8253	2597.3642	3	47.0095		0.0897
	872.8115	2615.3054	3	47.3687		0.1072
	945.0227	1887.9578	2	55.1189		0.0730
	980.0612	1958.0261	2	57.2738		0.0817
	984.5175	2950.4086	3	54.0711		0.1220
	1025.5705	2049.0531	2	60.0726		0.0733
	1143.9033	3428.5871	3	63.6342		0.1009
	1320.6548	3958.8207	3	74.2393		0.1217
	1417.4676	4249.2279	3	80.0481		0.1531
**40**						
	515.3138	1028.5688	2	28.6918	17	0.0442
	590.0189	1766.9526	3	30.4011		0.0823
	644.8700	1287.6679	2	36.6595		0.0576
	650.3428	1947.9149	3	34.0206		0.0915
	677.3682	2028.9939	3	35.6421		0.0888
	704.7050	2110.9953	3	37.2823		0.0979
	731.4296	2191.1637	3	38.8858		0.1032
	750.9495	1499.8208	2	43.1834		0.0637
**43**	772.7796	2315.2161	3	41.3668		0.1008
	804.4419	1606.8025	2	46.4732		0.0668
	821.1850	3280.5565	4	42.4499		0.1544
	871.5031	2611.3799	3	47.2902		0.1075
	984.5263	2950.4086	3	54.0716		0.1458
	1032.5805	2063.0687	2	60.5037		0.0777
	1042.9209	3125.6186	3	57.5753		0.1222
	1143.9096	3428.5871	3	63.6346		0.1199
	1320.6718	3958.8207	3	74.2403		0.1728
	621.8361	1241.5928	2	35.2429	9	0.0649
	637.8865	1273.7030	2	36.2300		0.0555
	684.8948	2735.4031	4	34.4088		0.1471
	694.0809	2079.1266	3	36.6449		0.0943
**44**	700.6430	2798.3736	4	35.3379		0.1694
	736.0777	2205.1371	3	39.1647		0.0741
	758.1046	2271.1760	3	40.4863		0.1161
	830.0070	1657.9151	2	48.0454		0.0844

Spot	Precursor ion *m*/*z*	Precursor ion mass	Ion charge	Collision Energy	Number of spectra for identification	Delta MS (ppm)
	858.2344	3428.7194	4	44.6358		0.1891
**45**	601.8723	1201.6489	2	34.0151	4	0.0811
	625.3751	1248.6649	2	35.4606		0.0707
	736.4183	2206.1230	3	39.1851		0.1100
	873.5283	1744.9393	2	50.7220		0.1028
**49**	483.2906	964.5495	2	26.7224	4	0.0172
	735.8304	1469.6939	2	42.2536		-0.0477
	773.9093	1545.8290	2	44.5954		-0.0251
	993.0000	1984.1204	2	58.0695		-0.1350
**50**	531.2869	1060.5917	2	29.6741	11	-0.0325
	588.7955	1175.6121	2	33.2109		-0.0356
	644.8010	1287.6208	2	36.6553		-0.0322
	655.0737	2616.3424	4	32.6493		-0.0768
	706.0682	2820.3330	4	35.6580		-0.0891
	770.3496	2308.1124	3	41.2210		-0.0856
	778.3722	2332.1699	3	41.7023		-0.0751
	795.8602	1589.7561	2	45.9454		-0.0504
	822.3772	2464.2135	3	44.3426		-0.1038
	896.9062	1791.8692	2	52.1597		-0.0715
	923.0554	2766.2594	3	50.3833		-0.1149
**51**	553.6334	1657.9151	3	28.2180	3	-0.0369
	637.8463	1273.7030	2	36.2275		-0.0250
	694.0291	2079.1266	3	36.6417		-0.0611
	531.9477	1592.8596	3	26.9169	34	-0.0382
	550.2841	1647.8654	3	28.0170		-0.0349
	554.2881	1659.8800	3	28.2573		-0.0375
	574.3103	1719.9632	3	29.4586		-0.0542
	595.3045	1782.9476	3	30.7183		-0.0558
	636.8319	1271.6874	2	36.1652		-0.0382
	642.3061	2565.2322	4	31.8961		-0.0369
	651.8293	1301.6835	2	37.0875		-0.0394
	653.3416	1304.7088	2	37.1805		-0.0403
	675.3342	2023.0197	3	35.5201		-0.0390
	696.3604	2086.1211	3	36.7816		-0.0617
	714.0000	2139.0266	3	37.8400		-0.0484
	720.0395	2157.1582	3	38.2024		-0.0615
	726.7078	2177.1481	3	38.6025		-0.0466

Spot	Precursor ion *m/z*	Precursor ion mass	Ion charge	Collision Energy	Number of spectra for identification	Delta MS (ppm)
	742.3832	1482.7943	2	42.6566		-0.0425
	771.3794	1540.7919	2	44.4398		-0.0477
**52**	778.8923	3111.6030	4	39.9546		-0.0628
	804.3861	1606.8025	2	46.4697		-0.0448
	807.0421	2418.1600	3	43.4225		-0.0554
	827.3889	1652.8055	2	47.8844		-0.0423
	828.1354	3308.5878	4	42.8600		-0.0755
	830.0556	2487.2104	3	44.8033		-0.0654
	866.7508	2597.3642	3	47.0050		-0.1336
	872.7589	2615.3054	3	47.3655		-0.0506
	927.1322	2778.4334	3	50.6279		-0.0587
	944.9593	1887.9578	2	55.1150		-0.0537
	971.9965	1942.0193	2	56.7778		-0.0409
	979.9996	1958.0261	2	57.2700		-0.0414
	984.4558	2950.4086	3	54.0673		-0.0631
	1025.5045	2049.0531	2	60.0685		-0.0586
	1059.2886	4233.2330	4	56.4980		-0.1078
	1127.5114	3379.6058	3	62.6507		-0.0933
	1143.8336	3428.5871	3	63.6300		-0.1082
	1320.5765	3958.8207	3	74.2346		-0.1132
	550.2863	1647.8654	3	28.0172	12	-0.0283
	639.8089	1277.6438	2	36.3482		-0.0406
	642.3037	2565.2322	4	31.8959		-0.0464
	726.7052	2177.1481	3	38.6023		-0.0541
	742.3883	1482.7943	2	42.6569		-0.0323
	804.3967	1606.8025	2	46.4704		-0.0236
**53**	944.9593	1887.9578	2	55.1150		-0.0537
	980.4923	1959.0101	2	57.3003		-0.0401
	984.4499	2950.4086	3	54.0670		-0.0808
	1025.5165	2049.0531	2	60.0693		-0.0346
	1059.5416	4234.2170	4	56.5130		-0.0797
	1149.1776	3444.5820	3	63.9507		-0.0711
	522.3051	1042.6023	2	29.1218	11	-0.0067
	555.7776	1109.5546	2	31.1803		-0.0139
	606.3223	1210.6598	2	34.2888		-0.0297
	628.3487	1882.0411	3	32.7009		-0.0169
	645.3339	1288.6697	2	36.6880		-0.0164
**54**	729.7207	2186.1630	3	38.7832		-0.0228
	777.9045	1553.8301	2	44.8411		-0.0356

Spot	Precursor ion *m*/*z*	Precursor ion mass	Ion charge	Collision Energy	Number of spectra for identification	Delta MS (ppm)
	971.8330	2912.5324	3	53.3100		-0.0552
	999.4654	1996.9445	2	58.4671		-0.0283
	1016.0241	2030.0619	2	59.4855		-0.0282
	1099.5723	3295.7605	3	60.9743		-0.0653
**55**	524.9469	1571.8307	3	26.4968	10	-0.0118
	554.3049	1659.8832	3	28.2583		0.0097
	617.8267	1233.6605	2	34.9963		-0.0216
	709.3541	1416.7283	2	40.6253		-0.0347
	762.8824	1523.7503	2	43.9173		-0.0481
	782.8778	1563.8045	2	45.1470		-0.0634
	846.4042	1690.8427	2	49.0539		-0.0489
	911.4241	1820.9017	2	53.0526		-0.0680
	912.0000	1821.8931	2	53.0880		0.0924
	998.4493	2992.4342	3	54.9070		-0.1082
**59**	414.7229	827.4694	2	22.5055	14	-0.0382
	523.2645	1044.5604	2	29.1808		-0.0459
	587.2601	2345.1301	4	28.6483		-0.1188
	634.8000	2535.1601	4	31.4532		0.0108
	638.6012	1912.8778	3	33.3161		-0.0961
	650.2950	1947.9592	3	34.0177		-0.0960
	691.9751	2072.9915	3	36.5185		-0.0880
	698.6536	2093.0429	3	36.9192		-0.1041
	711.6493	2132.0287	3	37.6990		-0.1025
	740.8196	1479.6994	2	42.5604		-0.0748
	774.0951	3092.4893	4	39.6716		-0.1382
	990.4336	2968.4243	3	54.4260		-0.1453
	1126.9894	2252.0750	2	66.3098		-0.1107
	1305.0501	2608.2333	2	77.2606		-0.1476
**61**	393.2619	784.5058	2	21.1856	15	0.0034
	598.8650	1195.7177	2	33.8302		-0.0023
	626.3303	1250.6911	2	35.5193		-0.0451
	655.0728	1962.2129	3	34.3044		-0.0162
	673.9977	2018.9884	3	35.4399		-0.0172
	696.3744	1390.7708	2	39.8270		-0.0365
	709.3865	2833.5086	4	35.8538		0.0083
	739.7306	2216.1913	3	39.3838		-0.0213
	740.3880	2957.5683	4	37.6829		-0.0454
	829.3264	1656.6838	2	48.0036		-0.0456

Spot	Precursor ion *m*/*z*	Precursor ion mass	Ion charge	Collision Energy	Number of spectra for identification	Delta MS (ppm)
	830.3922	2488.2533	3	44.8235		-0.0985
	894.7216	3574.8856	4	46.7886		-0.0285
	952.0318	1902.0491	2	55.5500		-0.0375
	954.3000	3813.0201	4	50.3037		0.1508
	965.4481	1928.8817	2	56.3751		-0.0623
**64**	927.9880	3708.0614	4	48.7513	1	-0.1386
	639.9724	1916.9632	3	33.3983	5	-0.0679
	658.3308	1972.0364	3	34.4998		-0.0657
**66**	727.0462	2178.1783	3	38.6228		-0.0617
	796.9069	1591.8457	2	46.0098		-0.0465
	855.1119	2562.4130	3	46.3067		-0.0993
	404.5543	1210.6598	3	19.2733	17	-0.0187
	471.5870	1411.7711	3	23.2952		-0.0318
	522.2944	1042.6023	2	29.1211		-0.0281
	542.2782	1082.5648	2	30.3501		-0.0229
	545.3099	1632.9312	3	27.7186		-0.0232
	555.7666	1109.5546	2	31.1796		-0.0360
	581.6376	1741.9210	3	29.8983		-0.0300
	628.3346	1882.0160	3	32.7001		-0.0341
**67**	633.0132	1896.0568	3	32.9808		-0.0391
	645.3149	1288.6697	2	36.6869		-0.0545
	666.6337	1996.9445	3	34.9980		-0.0653
	729.7055	2186.1630	3	38.7823		-0.0684
	767.7053	2300.1437	3	41.0623		-0.0495
	784.7221	2351.2076	3	42.0833		-0.0631
	858.0506	2571.1864	3	46.4830		-0.0566
	1016.0092	2030.0619	2	59.4846		-0.0580
	1023.8614	3068.6335	3	56.4317		-0.0710
	513.2725	1024.5441	2	28.5663	16	-0.0137
	574.3103	1719.9348	3	29.4586		-0.0259
	599.3060	1196.6190	2	33.8573		-0.0216
	612.6304	1834.9061	3	31.7578		-0.0366
	614.3356	1840.0094	3	31.8601		-0.0245
	627.0017	1878.0152	3	32.6201		-0.0319
	659.8458	1317.6969	2	37.5805		-0.0199
	717.3276	1432.6682	2	41.1156		-0.0275
**69**	718.6729	2153.0245	3	38.1204		-0.0277
	755.7339	2264.2205	3	40.3440		-0.0408

Spot	Precursor ion *m*/*z*	Precursor ion mass	Ion charge	Collision Energy	Number of spectra for identification	Delta MS (ppm)
	778.7564	2333.2896	3	41.7254		-0.0423
	794.3864	2380.1771	3	42.6632		-0.0397
	810.3930	1618.7951	2	46.8392		-0.0236
	927.9223	1853.8651	2	54.0672		-0.0350
	1004.5205	2007.0830	2	58.7780		-0.0401
	1312.6169	3934.9047	3	73.7570		-0.0760
**74**	485.7193	969.4556	2	26.8717	13	-0.0316
	523.2765	1044.5638	2	29.1815		-0.0253
	539.7785	1077.5488	2	30.1964		-0.0064
	589.9794	1766.9526	3	30.3988		-0.0363
	652.2973	1302.6312	2	37.1163		-0.0511
	709.9829	2126.9902	3	37.5990		-0.0635
	726.6969	2177.1004	3	38.6018		-0.0316
	763.8529	1525.7736	2	43.9770		-0.0824
	772.7137	2315.2161	3	41.3628		-0.0969
	782.3826	3125.6186	4	40.1606		-0.1173
	871.7650	2611.2233	3	47.3059		-0.0906
	1025.4708	2049.0055	2	60.0665		-0.0783
	1149.4723	3445.5660	3	63.9683		-0.1710
**78**	489.9197	1466.7558	3	24.3952	8	-0.0185
	585.2757	1168.5553	2	32.9945		-0.0185
	603.8340	1205.6656	2	34.1358		-0.0122
	653.3583	1304.7089	2	37.1815		-0.0068
	661.6901	1982.0796	3	34.7014		-0.0312
	784.4335	1566.8730	2	45.2427		-0.0205
	872.9720	1743.9560	2	50.6878		-0.0266
	1144.6066	2287.2253	2	67.3933		-0.0265
**83**	414.7686	827.4694	2	22.5083	10	0.0533
	634.5506	2534.1761	4	31.4385		-0.0028
	698.6808	2093.0429	3	36.9208		-0.0224
	702.3448	1402.6728	2	40.1942		0.0023
	711.6818	2132.0287	3	37.7009		-0.0051
	740.8553	1479.6994	2	42.5626		-0.0034
	870.4195	2608.2333	3	47.2252		0.0033
	990.4660	2968.4243	3	54.4280		-0.0481
	1037.4833	2072.9915	2	60.8052		-0.0396

Spot	Precursor ion *m/z*	Precursor ion mass	Ion charge	Collision Energy	Number of spectra for identification	Delta MS (ppm)
	1127.0334	2252.0750	2	66.3126		-0.0227
86	587.9776	1760.9230	3	30.2787	13	-0.0120
	650.9616	1949.8643	3	34.0577		-0.0014
	653.6864	1958.0374	3	34.2212		-0.0002
	666.8731	2663.4759	4	33.3455		-0.0124
	672.7055	2015.0952	3	35.3623		-0.0007
	698.0348	2091.0994	3	36.8821		-0.0169
	723.9052	1445.7959	2	41.5202		-0.0205
	737.0767	2208.2154	3	39.2246		-0.0070
	738.8859	1475.7773	2	42.4415		-0.0201
	807.4174	2419.2496	3	43.4450		-0.0193
	836.7913	2507.3748	3	45.2075		-0.0226
	854.4026	1706.7974	2	49.5458		-0.0067
	911.4673	1820.9554	2	53.0552		-0.0354
87	686.8916	1371.7795	2	39.2438	2	-0.0109
	717.3728	1432.7562	2	41.1184		-0.0252
96	485.7399	969.4556	2	26.8730	13	0.0097
	523.2722	1044.5638	2	29.1812		-0.0339
	539.7676	1077.5488	2	30.1957		-0.0282
	589.9748	1766.9526	3	30.3985		-0.0499
	652.3117	1302.6312	2	37.1172		-0.0224
	709.9779	2126.9902	3	37.5987		-0.0785
	726.7272	2177.1004	3	38.6036		0.0593
	763.8503	1525.7736	2	43.9768		-0.0876
	772.7137	2315.2161	3	41.3628		-0.0969
	782.3826	3125.6186	4	40.1606		-0.1173
	871.4221	2611.3799	3	47.2853		-0.1353
	1025.4798	2049.0055	2	60.0670		-0.0603
	1056.0285	4220.0851	4	56.3057		-0.1051
104	568.3103	1701.9202	3	29.0986	3	-0.0111
	623.0373	1866.0938	3	32.3822		-0.0039
	695.8662	1389.7252	2	39.7958		-0.0074
	500.9462	1499.8208	3	25.0568	17	-0.0040
	536.6307	1606.8752	3	27.1978		-0.0049
	580.9692	1739.8995	3	29.8582		-0.0138
	589.9893	1766.9526	3	30.3994		-0.0064

Spot	Precursor ion *m*/*z*	Precursor ion mass	Ion charge	Collision Energy	Number of spectra for identification	Delta MS (ppm)
	652.3221	1302.6312	2	37.1178		-0.0016
	677.3340	2028.9939	3	35.6400		-0.0136
	709.9962	2126.9902	3	37.5998		-0.0236
	726.7179	2177.1004	3	38.6031		0.0314
**107**	742.4036	1482.7943	2	42.6578		-0.0016
	763.8873	1525.7736	2	43.9791		-0.0135
	772.7353	2315.2161	3	41.3641		-0.0320
	782.4096	3125.6186	4	40.1622		-0.0094
	812.3754	1622.7974	2	46.9611		-0.0612
	871.4616	2611.3799	3	47.2877		-0.0169
	990.7137	3958.8207	4	52.4521		0.0048
	1025.5225	2049.0055	2	60.0696		0.0250
	1149.1903	3444.5820	3	63.9514		-0.0330
	518.2628	1551.7722	3	26.0958	14	-0.0055
	672.5913	2686.3384	4	33.6829		-0.0023
	711.3570	2131.0520	3	37.6814		-0.0029
	712.8467	1423.6772	2	40.8401		0.0016
	753.0763	2256.1961	3	40.1846		0.0110
	755.3631	2263.0790	3	40.3218		-0.0115
**108**	780.4014	2338.1957	3	41.8241		-0.0135
	786.3891	2356.1699	3	42.1833		-0.0244
	808.0696	2421.1886	3	43.4842		-0.0017
	891.2040	3560.8257	4	46.5810		-0.0388
	978.1474	2931.4250	3	53.6888		-0.0047
	980.8707	2939.6008	3	53.8522		-0.0105
	1019.5023	2037.0055	2	59.6994		-0.0153
	1032.9116	3095.7019	3	56.9747		0.0110
	472.2789	942.5498	2	26.0452	3	-0.0067
**111**	570.3195	1707.9420	3	29.2192		-0.0055
	674.3779	1346.7558	2	38.4742		-0.0145
	553.6179	1657.8383	3	28.2171	12	-0.0063
	580.9827	1739.9359	3	29.8590		-0.0095
	613.0478	2448.1768	4	30.1698		-0.0147
	656.3401	1310.6772	2	37.3649		-0.0116
	688.8643	1375.7347	2	39.3652		-0.0208
**112**	704.9000	2815.4538	4	35.5891		0.1171
	752.9144	1503.8297	2	43.3042		-0.0155

Spot	Precursor ion *m*/*z*	Precursor ion mass	Ion charge	Collision Energy	Number of spectra for identification	Delta MS (ppm)
	769.0736	2304.1961	3	41.1444		0.0029
	830.1285	2487.3809	3	44.8077		-0.0172
	1039.0406	2076.0963	2	60.9010		-0.0297
	1039.5207	3115.5608	3	57.3712		-0.0205
	1158.0822	2314.1805	2	68.2221		-0.0307
**114**	494.6000	1480.7674	3	24.6760	8	0.0107
	554.2793	1106.5608	2	31.0882		-0.0168
	581.9765	1742.9203	3	29.9186		-0.0126
	719.6852	2156.0525	3	38.1811		-0.0188
	736.4284	1470.8599	2	42.2903		-0.0176
	747.9085	2987.6194	4	38.1266		-0.0147
	750.8956	1499.7984	2	43.1801		-0.0217
	872.1309	2613.4128	3	47.3279		-0.0420
**134**	468.2449	1401.7194	3	23.0947	13	-0.0064
	641.3213	1280.6401	2	36.4413		-0.0120
	643.6480	1927.9309	3	33.6189		-0.0087
	659.6990	1976.0612	3	34.5819		0.0140
	704.3594	2813.4083	4	35.5572		0.0003
	751.3346	1500.6766	2	43.2071		-0.0220
	753.2000	3008.6295	4	38.4388		0.1414
	766.7298	2297.2744	3	41.0038		-0.1067
	801.4030	1600.7984	2	46.2863		-0.0071
	808.0962	2421.2838	3	43.4858		-0.0171
	924.4903	1846.9823	2	53.8562		-0.0163
	982.9399	1963.8986	2	57.4508		-0.0333
	1120.9226	3359.8017	3	62.2554		-0.0557
**136**	494.8976	1481.6841	3	24.6939	16	-0.0131
	537.9768	1610.9355	3	27.2786		-0.0269
	542.3371	1082.6699	2	30.3537		-0.0102
	570.2881	1707.8620	3	29.2173		-0.0194
	577.5989	1729.8060	3	29.6559		-0.0311
	594.8019	1187.6186	2	33.5803		-0.0295
	619.6402	1855.9138	3	32.1784		-0.0151
	658.1290	2628.5116	4	32.8296		-0.0247
	681.1000	2720.2209	4	34.1849		0.1500
	689.3560	2065.0732	3	36.3614		-0.0269
	717.3477	1432.6987	2	41.1169		-0.0178
	738.3131	1474.6365	2	42.4063		-0.0248
	761.3860	3041.5492	4	38.9218		-0.0342
	832.1726	3324.7202	4	43.0982		-0.0587

Spot	Precursor ion *m*/*z*	Precursor ion mass	Ion charge	Collision Energy	Number of spectra for identification	Delta MS (ppm)
	962.8255	2885.4481	3	52.7695		0.0066
	1071.1375	3210.4703	3	59.2683		-0.0797
**139**	609.6573	1825.9785	3	31.5794	2	-0.0285
	664.3648	1326.7395	2	37.8584		-0.0245
**145**	494.6038	1480.8112	3	24.6762	20	-0.0215
	521.8193	1041.6434	2	29.0919		-0.0194
	542.7909	1083.5812	2	30.3816		-0.0139
	590.2988	2357.1897	4	28.8276		-0.0236
	600.6504	1798.9577	3	31.0390		-0.0283
	611.8378	2443.3475	4	30.0984		-0.0253
	639.6762	1916.0353	3	33.3806		-0.0285
	640.6479	1918.9537	3	33.4389		-0.0320
	667.3183	1998.9858	3	35.0391		-0.0528
	683.3441	2047.0487	3	36.0006		-0.0384
	694.8560	1387.7347	2	39.7336		-0.0373
	710.3731	2128.1376	3	37.6224		-0.0402
	713.6297	2850.5353	4	36.1042		-0.0458
	770.4405	1538.8920	2	44.3821		-0.0254
	787.0853	2358.2682	3	42.2251		-0.0341
	811.0971	2430.3217	3	43.6658		-0.0523
	867.9922	1734.0039	2	50.3815		-0.0342
	884.9292	1767.8654	2	51.4231		-0.0215
	910.4411	2728.3571	3	49.6265		-0.0557
	1149.5459	2297.1250	2	67.6971		-0.0477
**147**	695.8093	2779.3593	4	35.0527	5	-0.1510
	770.3808	1538.8344	2	44.3784		-0.0875
	771.8789	1541.8276	2	44.4706		-0.0842
	871.3427	1740.7995	2	50.5876		-0.1286
	992.9201	1983.9513	2	58.0646		-0.1256
**148**	587.2805	1758.9152	3	30.2368	7	-0.0954
	648.3092	1942.0425	3	33.8986		-0.1366
	752.3568	1502.7882	2	43.2699		-0.0892
	780.7232	2339.3114	3	41.8434		-0.1635
	807.3801	2419.2496	3	43.4428		-0.1311
	836.7534	2507.3748	3	45.2052		-0.1364
	854.3506	1706.7974	2	49.5426		-0.1107
	588.9887	1764.0298	3	30.3393	15	-0.0855
	647.9968	1941.0643	3	33.8798		-0.0956
	707.6147	2826.5392	4	35.7493		-0.1096

Spot	Precursor ion *m*/*z*	Precursor ion mass	Ion charge	Collision Energy	Number of spectra for identification	Delta MS (ppm)
	730.5989	2918.4994	4	37.1053		-0.1328
	762.3711	2284.2328	3	40.7423		-0.1415
	763.3774	2287.2325	3	40.8026		-0.1221
	767.6950	2300.1868	3	41.0617		-0.1238
**149**	772.3708	3085.6461	4	39.5699		-0.1918
	773.3916	2317.2754	3	41.4035		-0.1224
	800.6660	3198.7764	4	41.2393		-0.1416
	849.9321	1697.9312	2	49.2708		-0.0816
	864.4000	3453.6180	4	44.9996		-0.0471
	866.4199	2596.3245	3	46.9852		-0.0866
	873.4065	1744.8995	2	50.7145		-0.1011
	1066.5105	2131.1314	2	62.5904		-0.1249
	661.0734	2640.3911	4	33.0033	10	-0.1266
	735.0286	2202.1644	3	39.1017		-0.1006
	768.3621	2302.1594	3	41.1017		-0.0950
	772.4021	3085.6924	4	39.5717		-0.1132
**154**	773.3577	2317.1550	3	41.4015		-0.1036
	808.0350	2421.1886	3	43.4821		-0.1056
	825.0977	2472.3588	3	44.5059		-0.0875
	840.3798	1678.8349	2	48.6834		-0.0897
	951.7898	2852.4742	3	52.1074		-0.1266
	966.4794	1931.0364	2	56.4385		-0.0922
	552.7852	1103.5975	2	30.9963	14	-0.0417
	617.9859	1850.9890	3	32.0792		-0.0532
	623.6804	1868.0619	3	32.4208		-0.0426
	678.3412	2032.0663	3	35.7005		-0.0646
	701.7070	2102.1082	3	37.1024		-0.0089
	717.8621	1433.7514	2	41.1485		-0.0418
	749.9001	1497.8403	2	43.1189		-0.0547
**163**	769.7206	2306.2305	3	41.1832		-0.0906
	824.3956	2470.2281	3	44.4637		-0.0631
	868.1108	2601.3836	3	47.0866		-0.0730
	888.3793	1774.7951	2	51.6353		-0.0510
	954.4386	2860.2939	3	52.2663		-0.0881
	995.7821	2984.4251	3	54.7469		-0.1005
	1100.4826	2199.0332	2	64.6797		-0.0826
	555.7776	1109.5546	2	31.1803	9	-0.0139
	606.3085	1210.6598	2	34.2880		-0.0574
	729.7106	2186.1630	3	38.7826		-0.0532
	767.7001	2300.1437	3	41.0620		-0.0651
**164**	784.9137	1567.8457	2	45.2722		-0.0330

Spot	Precursor ion *m*/*z*	Precursor ion mass	Ion charge	Collision Energy	Number of spectra for identification	Delta MS (ppm)
	858.0396	2571.1864	3	46.4824		-0.0895
	1016.0182	2030.0619	2	59.4851		-0.0401
	1023.8555	3068.6335	3	56.4313		-0.0890
	1099.5723	3295.7605	3	60.9743		-0.0653
**170**	552.2744	1653.8878	3	28.1365	5	-0.0865
	706.3744	1410.7343	2	40.4420		-0.0204
	723.0058	2166.0269	3	38.3803		-0.0313
	934.7798	2801.3177	3	51.0868		-0.0754
	962.4216	1922.8938	2	56.1889		-0.0653
**172**	617.7918	1233.6030	2	34.9942	8	-0.0340
	631.8440	1261.6918	2	35.8584		-0.0184
	744.8727	1487.7831	2	42.8097		-0.0522
	752.4408	1502.8933	2	43.2751		-0.0263
	821.4312	1640.8774	2	47.5180		-0.0296
	826.7497	2477.2843	3	44.6050		-0.0569
	842.4005	1682.8264	2	48.8076		-0.0400
	1013.4634	3037.4855	3	55.8078		-0.1171
**174**	551.6288	1651.8570	3	28.0977	13	0.0075
	630.6626	1888.9795	3	32.8398		-0.0134
	665.8471	1329.7041	2	37.9496		-0.0244
	723.8616	1445.7514	2	41.5175		-0.0428
	769.8575	1537.7269	2	44.3462		-0.0264
	821.7265	2462.2594	3	44.3036		-0.1017
	825.4144	1648.8461	2	47.7630		-0.0317
	943.3930	2827.2937	3	51.6036		-0.1366
	1011.4815	2021.0040	2	59.2061		-0.0556
	1016.1339	3045.5155	3	55.9680		-0.1356
	1171.5366	2341.1649	2	69.0495		-0.1063
	1200.4932	2399.0917	2	70.8303		-0.1200
	1253.0164	2504.1343	2	74.0605		-0.1161
**175**						
**178**	672.5816	2686.3384	4	33.6823	12	-0.0412
	712.8292	1423.6772	2	40.8390		-0.0334
	753.0557	2256.1961	3	40.1833		-0.0507
	780.3909	2338.1957	3	41.8235		-0.0449
	786.3786	2356.1699	3	42.1827		-0.0559
	891.2012	3560.8257	4	46.5809		-0.0500
	959.8023	2876.4550	3	52.5881		-0.0698
	978.1210	2931.4250	3	53.6873		-0.0838
	980.8560	2939.6008	3	53.8514		-0.0545
	1019.4904	2037.0055	2	59.6987		-0.0393

Spot	Precursor ion *m*/*z*	Precursor ion mass	Ion charge	Collision Energy	Number of spectra for identification	Delta MS (ppm)
	1032.8815	3095.7019	3	56.9729		-0.0793
	1211.5695	2421.1886	2	71.5115		-0.0642
**179**	649.8556	1297.7428	2	36.9661	10	-0.0462
	686.0278	2055.1299	3	36.1617		-0.0684
	707.0193	2118.0779	3	37.4212		-0.0418
	736.7106	2207.1528	3	39.2026		-0.0428
	835.9706	1669.9767	2	48.4122		-0.0501
	905.0651	2712.2490	3	49.3039		-0.0755
	932.4502	2794.3946	3	50.9470		-0.0658
	993.4367	2977.3527	3	54.6062		-0.0645
	1041.8352	3122.5441	3	57.5101		-0.0602
	1092.0348	4364.2151	4	58.4301		-0.1051
**185**	757.3518	1512.7130	2	43.5771	2	-0.0240
	1008.0266	2014.0921	2	58.9936		-0.0535
**186**	746.6436	2982.6138	4	38.0520	3	-0.0686
	757.3286	1512.7130	2	43.5757		-0.0704
	1008.0266	2014.0921	2	58.9936		-0.0535
**188**	694.0340	2079.1266	3	36.6420	4	-0.0463
	700.5885	2798.3736	4	35.3347		-0.0488
	829.9504	1657.9151	2	48.0419		-0.0289
	1063.9976	2126.0545	2	62.4359		-0.0738
**189**	638.6414	1912.9492	3	33.3185	3	-0.0468
	698.9755	2093.9615	3	36.9385		-0.0570
	929.3980	1856.8369	2	54.1580		-0.0554
**192**	600.2740	1198.5659	2	33.9169	10	-0.0324
	622.2800	1863.8693	3	32.3368		-0.0511
	623.3085	1866.9462	3	32.3985		-0.0425
	817.4717	2449.4560	3	44.0483		-0.0627
	840.4722	2518.4662	3	45.4283		-0.0715
	866.9190	1731.8832	2	50.3155		-0.0597
	875.9911	1750.0280	2	50.8735		-0.0604
	903.9636	1805.9498	2	52.5938		-0.0371
	907.4562	1812.9622	2	52.8086		-0.0643
	1141.5602	2281.1801	2	67.2060		-0.0743
	552.7998	1103.5850	2	30.9972	18	-0.0125
	618.3390	1851.9730	3	32.1003		0.0222
	623.6982	1868.0619	3	32.4219		0.0110
	653.3431	1304.6435	2	37.1806		0.0282
	717.8788	1433.7514	2	41.1495		-0.0083

Spot	Precursor ion *m*/*z*	Precursor ion mass	Ion charge	Collision Energy	Number of spectra for identification	Delta MS (ppm)
**196**	749.9096	1497.8403	2	43.1194		-0.0357
	764.3836	2290.2355	3	40.8630		-0.1064
	824.9463	1647.9018	2	47.7342		-0.0238
	862.7837	2585.3887	3	46.7670		-0.0594
	863.4111	1724.8443	2	50.0998		-0.0367
	863.4359	1724.8920	2	50.1013		-0.0348
	888.3786	1774.7951	2	51.6353		-0.0525
	935.0025	1868.0619	2	54.5027		-0.0715
	995.7726	2984.4251	3	54.7464		-0.1292
	1044.0155	2086.1133	2	61.2070		-0.0968
	1076.8032	3227.4930	3	59.6082		-0.1051
	1086.4822	4342.0126	4	58.1024		-0.1131
	1100.4757	2199.0332	2	64.6793		-0.0963
**197**	540.2713	1617.8264	3	27.4163	8	-0.0344
	581.9856	1742.9791	3	29.9191		-0.0443
	638.6177	1912.8778	3	33.3171		-0.0464
	732.3570	2194.1032	3	38.9414		-0.0540
	832.4050	1662.8287	2	48.1929		-0.0332
	840.3934	2518.2227	3	45.4236		-0.0643
	1060.4717	2119.0110	2	62.2190		-0.0821
	1156.0112	2310.0804	2	68.0947		-0.0726
**200**						
**203**						
**205**	417.7394	833.4395	2	22.6910	17	0.0246
	538.3128	1074.6186	2	30.1062		-0.0076
	600.3328	1198.6346	2	33.9205		0.0165
	623.6936	1868.0619	3	32.4216		-0.0031
	717.8688	1433.7514	2	41.1489		-0.0284
	749.9147	1497.8403	2	43.1198		-0.0255
	769.7380	2306.2305	3	41.1843		-0.0384
	828.7277	2483.2233	3	44.7237		-0.0621
	838.9786	1675.9661	2	48.5972		-0.0236
	839.9257	1677.8760	2	48.6554		-0.0392
	870.4547	2608.3683	3	47.2273		-0.0262
	894.8757	1787.7903	2	52.0349		-0.0536
	935.0111	1868.0619	2	54.5032		-0.0543
	987.4373	2959.3757	3	54.2462		-0.0856

Spot	Precursor ion *m*/*z*	Precursor ion mass	Ion charge	Collision Energy	Number of spectra for identification	Delta MS (ppm)
	989.7727	2966.3822	3	54.3864		-0.0858
	1085.8092	3254.5403	3	60.1486		-0.1345
	1103.9554	2205.9736	2	64.8933		-0.0773
**206**	441.7679	881.5011	2	24.1687	5	0.0201
	541.9655	1622.8603	3	27.5179		0.0145
	604.2659	1206.5703	2	34.1624		-0.0530
	755.3366	2263.0627	3	40.3202		-0.0748
	1199.5717	2397.1668	2	70.7737		-0.0381
**207**	530.3186	1058.5760	2	29.6146	2	0.0467
	599.8835	1197.7081	2	33.8928		0.0444
**210**	550.3303	1098.6550	2	30.8453	11	-0.0090
	600.3176	1797.9526	3	31.0191		-0.0216
	615.9664	1844.8581	3	31.9580		0.0194
	654.6590	1960.9789	3	34.2795		-0.0237
	658.3308	1971.9730	3	34.4998		-0.0023
	682.9891	2045.9582	3	35.9793		-0.0128
	707.6820	2120.0215	3	37.4609		0.0026
	738.3666	1474.7602	2	42.4095		-0.0416
	849.3997	1696.8097	2	49.2381		-0.0249
	936.1635	2805.4912	3	51.1698		-0.0225
	991.0321	1980.0568	2	57.9485		-0.0071
**213**	516.2838	1545.8515	3	25.9770	4	-0.0219
	711.6968	2132.0803	3	37.7018		-0.0118
	743.3712	1484.7663	2	42.7173		-0.0386
	861.4396	1720.8937	2	49.9785		-0.0290
**214**	617.8019	1233.6030	2	34.9948	4	-0.0137
	631.8496	1261.6918	2	35.8588		-0.0073
	826.7559	2477.2843	3	44.6054		-0.0385
	1216.0826	2430.1526	2	71.7891		-0.0019
**219**	717.3853	1432.7674	2	41.1192	7	-0.0113
	738.3844	1474.7820	2	42.4106		-0.0278
	744.4160	1486.8429	2	42.7816		-0.0254
	746.8995	1491.8086	2	42.9343		-0.0240
	821.4346	2461.2587	3	44.2861		0.0233
	982.0210	1962.0574	2	57.3943		-0.0301
	1090.5217	3268.5962	3	60.4313		-0.0528

Spot	Precursor ion *m/z*	Precursor ion mass	Ion charge	Collision Energy	Number of spectra for identification	Delta MS (ppm)
**242**	553.6488	1657.9151	3	28.2189	7	0.0094
	621.8058	1241.5928	2	35.2411		0.0042
	694.0562	2079.1266	3	36.6434		0.0203
	700.5934	2798.3736	4	35.3350		-0.0289
	715.3672	2143.0811	3	37.9220		-0.0013
	736.0370	2205.1371	3	39.1622		-0.0478
	752.3979	2254.1495	3	40.1439		0.0223
**245**	555.7798	1109.5546	2	31.1805	3	-0.0095
	784.9163	1567.8457	2	45.2724		-0.0277
	971.8534	2912.5324	3	53.3112		0.0061
**249**	542.3306	1082.6699	2	30.3533	4	-0.0233
	570.2971	1707.8620	3	29.2178		0.0074
	614.3147	1839.9189	3	31.8589		0.0034
	854.9366	1707.8620	2	49.5786		-0.0035
**251**	623.6921	1868.0619	3	32.4215	10	-0.0076
	696.3752	2086.1133	3	36.7825		-0.0094
	749.9104	1497.8403	2	43.1195		-0.0341
	764.4129	2290.2355	3	40.8648		-0.0186
	862.7955	2585.3887	3	46.7677		-0.0242
	888.3960	1774.7951	2	51.6364		-0.0175
	989.7881	2966.3822	3	54.3873		-0.0396
	1071.1620	3210.5141	3	59.2697		-0.0499
	1082.5037	4326.0177	4	57.8677		-0.0320
	1100.5012	2199.0332	2	64.6808		-0.0453
**252**	505.9101	1514.7419	3	25.3546	11	-0.0333
	539.2681	1614.8002	3	27.3561		-0.0178
	599.8404	1197.6982	2	33.8902		-0.0321
	652.0207	1953.0571	3	34.1212		-0.0169
	775.8969	1549.8021	2	44.7177		-0.0230
	776.3718	2326.1627	3	41.5823		-0.0692
	782.3939	1562.7974	2	45.1172		-0.0242
	788.7000	3150.6350	4	40.5333		0.1359
	867.7604	2600.2857	3	47.0656		-0.0264
	874.4282	1746.8788	2	50.7773		-0.0369
	935.4644	1868.9407	2	54.5311		-0.0265
**257**						
**261**	792.9747	2376.0252	3	42.5785	1	-0.1230
**262**						

Spot	Precursor ion *m/z*	Precursor ion mass	Ion charge	Collision Energy	Number of spectra for identification	Delta MS (ppm)
265	591.8313	1181.7020	2	33.3976	10	-0.0539
	636.3309	1270.7132	2	36.1344		-0.0660
	650.9879	1950.0422	3	34.0593		-0.1005
	672.6617	2015.0422	3	35.3597		-0.0788
	719.6927	2156.1689	3	38.1816		-0.1126
	771.3534	1540.7620	2	44.4382		-0.0699
	832.0430	2493.2269	3	44.9226		-0.1199
	871.7685	2612.4061	3	47.3061		-0.1223
	911.9339	1821.9472	2	53.0839		-0.0940
	1240.0140	2478.1624	2	73.2609		-0.1489
266	613.2658	1836.8101	3	31.7959	2	-0.0345
	797.8300	1593.6770	2	46.0665		-0.0316
267						
268	673.8453	1345.7315	2	38.4415	6	-0.0555
	687.3532	1372.7351	2	39.2722		-0.0432
	793.6949	2378.1390	3	42.6217		-0.0762
	825.4663	1648.9876	2	47.7662		-0.0694
	957.4620	2869.5081	3	52.4477		-0.1440
	1000.5228	1999.1288	2	58.5322		-0.0978
272	434.7696	867.5178	2	23.7383	6	0.0068
	533.9427	1598.8093	3	27.0366		-0.0030
	584.7900	1167.5520	2	32.9646		0.0135
	635.3239	1902.9608	3	33.1194		-0.0109
	723.3728	1444.7596	2	41.4874		-0.0286
	822.3952	1642.8162	2	47.5773		-0.0404
273	511.2649	1530.7943	3	25.6759	7	-0.0215
	552.2700	2205.1153	4	26.5839		-0.0644
	675.6506	2024.0255	3	35.5390		0.0954
	821.0588	2460.2107	3	44.2635		-0.0560
	838.1005	2511.3544	3	45.2860		-0.0746
	845.7593	2534.3209	3	45.7456		-0.0649
	948.9427	1895.9305	2	55.3600		-0.0597
274	481.9066	1442.7365	3	23.9144	12	-0.0384
	510.2615	1018.5448	2	28.3811		-0.0363
	533.7661	1065.5529	2	29.8266		-0.0352
	538.5853	1612.8056	3	27.3151		-0.0716
	626.2748	1250.5779	2	35.5159		-0.0427
	658.6265	1972.9279	3	34.5176		-0.0703
	791.7697	2372.3792	3	42.5062		-0.0918
	807.0049	2418.1121	3	43.4203		-0.1193

Spot	Precursor ion *m/z*	Precursor ion mass	Ion charge	Collision Energy	Number of spectra for identification	Delta MS (ppm)
	843.8046	2528.4803	3	45.6283		-0.0883
	863.3953	2528.4803	3	46.8037		-0.0694
	872.9498	1743.9382	2	50.6864		-0.0531
	991.5423	2971.6971	3	54.4925		-0.0922
276	680.6460	2039.0146	3	35.8388	5	-0.0983
	726.6825	2177.1481	3	38.6010		-0.1223
	812.3620	1622.7974	2	46.9603		-0.0879
	843.3639	1684.7953	2	48.8669		-0.0821
	984.4293	2950.4086	3	54.0658		-0.1425
278	646.6742	1937.0946	3	33.8005	10	-0.0937
	702.6874	2105.1269	3	37.1612		-0.0864
	775.0283	2322.1855	3	41.5017		-0.1225
	820.4095	1638.8941	2	47.4552		-0.0897
	842.0886	2523.3544	3	45.5253		-0.1104
	861.3819	2581.2409	3	46.6829		-0.1170
	895.0725	2682.3137	3	48.7044		-0.1179
	895.9218	1789.9210	2	52.0992		-0.0919
	935.9314	1869.9369	2	54.5598		-0.0886
	1134.5013	2267.1169	2	66.7718		-0.1289
282	559.2808	1674.8577	3	28.5568	2	-0.0370
	1006.4511	2011.0011	2	58.8967		-0.1134
284	537.2666	1072.6240	2	30.0419	16	-0.1054
	550.7610	1099.6125	2	30.8718		-0.1051
	598.2653	1194.6397	2	33.7933		-0.1236
	615.2552	1842.9111	3	31.9153		-0.1673
	642.7951	1283.7085	2	36.5319		-0.1328
	657.6292	1970.0507	3	34.4578		-0.1849
	657.6580	1970.1048	3	34.4595		-0.1525
	677.3071	1352.7300	2	38.6544		-0.1302
	721.2464	1440.6191	2	41.3567		-0.1409
	736.3190	1470.7865	2	42.2836		-0.1629
	779.3053	1556.7471	2	44.9273		-0.1510
	780.8749	1559.8923	2	45.0238		-0.1569
	798.7958	1595.7501	2	46.1259		-0.1730
	814.8947	1627.9145	2	47.1160		-0.1396
	907.9217	1814.0037	2	52.8372		-0.1748
	942.9506	1884.0608	2	54.9915		-0.1742
286	693.2963	1384.7449	2	39.6377	5	-0.1668
	712.3290	1422.7758	2	40.8082		-0.1323
	755.3785	2263.2827	3	40.3227		-0.1691
	886.4288	1770.9978	2	51.5154		-0.1547

Spot	Precursor ion *m/z*	Precursor ion mass	Ion charge	Collision Energy	Number of spectra for identification	Delta MS (ppm)
	922.9045	1843.9832	2	53.7586		-0.1887
287	591.8016	1181.7020	2	33.3958	11	-0.1133
	608.8342	1215.7590	2	34.4433		-0.1052
	615.2970	1228.6928	2	34.8408		-0.1133
	641.6629	1922.1201	3	33.4998		-0.1533
	642.8046	1283.7085	2	36.5325		-0.1138
	648.3163	1294.7357	2	36.8715		-0.1177
	698.2970	2092.0437	3	36.8978		-0.1744
	714.3379	2140.1739	3	37.8603		-0.1822
	753.2921	1504.7086	2	43.3275		-0.1388
	771.3195	1540.7620	2	44.4361		-0.1377
	942.4473	1883.0364	2	54.9605		-0.1564
292	618.9641	1854.0298	3	32.1378	13	-0.1592
	631.2916	1260.6925	2	35.8244		-0.1239
	639.2948	1915.0091	3	33.3577		-0.1466
	645.2982	1288.7067	2	36.6858		-0.1250
	686.8203	1371.7146	2	39.2394		-0.0885
	701.8261	1401.7715	2	40.1623		-0.1339
	780.3516	1558.8508	2	44.9916		-0.1622
	794.3969	1586.9072	2	45.8554		-0.1280
	815.3253	1628.7908	2	47.1425		-0.1549
	835.0445	2502.3053	3	45.1027		-0.1935
	849.8937	1697.9287	2	49.2685		-0.1558
	894.3720	1786.9142	2	52.0039		-0.1847
	990.4276	1979.0337	2	57.9113		-0.1924
305	550.3083	1647.9018	3	28.0185	15	0.0012
	552.7984	1103.5975	2	30.9971		-0.0152
	623.6757	1868.0619	3	32.4205		-0.0567
	678.3583	2032.0663	3	35.7015		-0.0134
	701.6996	2102.1082	3	37.1020		-0.0312
	717.8696	1433.7514	2	48.6554		-0.0268
	749.9129	1497.8403	2	48.6554		-0.0290
	769.7413	2306.2305	3	41.1845		-0.0283
	824.4064	2470.2281	3	44.4644		-0.0308
	868.1191	2601.3836	3	47.0871		-0.0482
	888.3988	1774.7951	2	51.6365		-0.0120
	926.9839	1851.9730	2	54.0095		-0.0198
	954.1145	2859.3980	3	52.2469		-0.0763
	995.7880	2984.4251	3	54.7473		-0.0828

Spot	Precursor ion *m*/*z*	Precursor ion mass	Ion charge	Collision Energy	Number of spectra for identification	Delta MS (ppm)
	1100.5012	2199.0332	2	64.6808		-0.0453
**309**	511.7330	1021.5484	2	28.4716	4	-0.0970
	537.5793	1609.8055	3	27.2548		-0.0895
	826.3195	1650.7930	2	47.8186		-0.1685
	1067.6000	2132.9862	2	62.6574		0.1993
**311**	521.7785	1041.6434	2	29.0894	17	-0.1008
	536.7847	1071.6440	2	30.0123		-0.0892
	537.7660	1073.6233	2	30.0726		-0.1058
	600.6021	1798.9577	3	31.0361		-0.1732
	601.5850	1801.8880	3	31.0951		-0.1548
	640.5980	1918.9537	3	33.4359		-0.1815
	649.7743	1297.6514	2	36.9611		-0.1173
	667.2722	1998.9858	3	35.0363		-0.1910
	673.3420	1344.8017	2	38.4105		-0.1322
	683.2926	2047.0487	3	35.9976		-0.1928
	694.8016	1387.7347	2	39.7303		-0.1461
	741.3346	1480.8112	2	42.5921		-0.1566
	770.3807	1538.8920	2	44.3784		-0.1452
	787.3402	1572.8359	2	45.4214		-0.1702
	867.9286	1734.0039	2	50.3776		-0.1613
	884.8483	1767.8654	2	51.4182		-0.1833
	958.9290	1916.0353	2	55.9741		-0.1920
**312**	421.7162	841.4850	2	22.9355	11	-0.0671
	540.2364	1617.8264	3	27.4142		-0.1391
	555.2477	1662.8287	3	28.3149		-0.1074
	584.2292	1166.5568	2	32.9301		-0.1129
	590.2873	1178.6659	2	33.3027		-0.1058
	638.5750	1912.8778	3	33.3145		-0.1745
	717.6537	2150.0895	3	38.0592		-0.1502
	718.7506	1435.6653	2	41.2032		-0.1786
	732.3138	2194.1032	3	38.9388		-0.1577
	840.3526	2518.2227	3	45.4212		-0.1868
	1060.4136	2119.0110	2	62.2154		-0.1982
**318**	628.8934	1883.8537	3	32.7336	9	-0.1953
	700.6529	2099.1085	3	37.0392		-0.1718
	703.3033	1404.7323	2	40.2532		-0.1403
	708.6340	2123.0721	3	37.5180		-0.1918
	711.2845	1420.7272	2	40.7440		-0.1728
	713.9649	2139.0670	3	37.8379		-0.1941
	720.7710	1439.6868	2	41.3274		-0.1593
	808.2852	1614.7171	2	46.7095		-0.1613

Spot	Precursor ion *m*/*z*	Precursor ion mass	Ion charge	Collision Energy	Number of spectra for identification	Delta MS (ppm)
	829.8424	1657.8498	2	48.0353		-0.1797
**319**	472.2381	942.4616	2	26.0426	4	-0.0882
	674.3243	1346.6341	2	38.4709		-0.1217
	570.2769	1707.8089	3	29.2166		-0.1332
	622.3033	1863.8880	3	32.3382		-0.1551
**321**	489.6999	977.4971	2	27.1165	17	-0.1118
	619.7800	1237.6554	2	35.1165		-0.1099
	646.6479	1937.0946	3	33.7989		-0.1726
	654.7835	1307.6721	2	37.2692		-0.1196
	655.2822	1963.0044	3	34.3169		-0.1797
	702.6551	2105.1269	3	37.1593		-0.1835
	768.3075	1534.7515	2	44.2509		-0.1511
	775.3333	2323.1695	3	41.5200		-0.1914
	776.3534	1550.8569	2	44.7457		-0.1646
	783.3713	1564.8937	2	45.1773		-0.1656
	801.3392	1600.8420	2	46.2824		-0.1781
	820.3584	1638.8941	2	47.4520		-0.1919
	840.3987	1638.8941	2	48.6845		-0.1689
	895.8769	1679.9358	2	52.0964		-0.1818
	918.3432	1834.8697	2	53.4781		-0.1980
	935.8769	1834.8697	2	54.5564		-0.1977
	1134.6000	2267.1169	2	66.7779		0.0685
**326**	918.3432	1834.8697	2	53.4781	2	-0.1980
	1134.4728	2267.1169	2	66.7701		-0.1859
**328**	665.2848	1328.6936	2	37.9150	4	-0.1384
	726.3012	1450.7304	2	41.6675		-0.1425
	730.8368	1459.8147	2	41.9465		-0.1556
	825.3613	1648.8308	2	47.7597		-0.1228
**329**	521.9048	1562.6925	3	26.3143	2	-0.1419
	709.2949	1416.7136	2	40.6216		-0.1383
**334**	603.7810	1205.6656	2	34.1325	6	-0.1182
	653.2912	1304.7089	2	37.1774		-0.1410
	661.6418	1982.0796	3	34.6985		-0.1760
	721.8477	1441.8253	2	41.3936		-0.1445
	784.3678	1566.8730	2	45.2386		-0.1518
	872.9027	1743.9560	2	50.6835		-0.1652
**335**	576.6088	1726.9465	3	29.5965	3	-0.1420
	623.2663	1244.6500	2	35.3309		-0.1319
	952.9228	1903.9965	2	55.6048		-0.1664
**336**	527.2702	1052.6131	2	29.4271	4	-0.0873
	646.3122	1290.7296	2	36.7482		-0.1198

Spot	Precursor ion *m*/*z*	Precursor ion mass	Ion charge	Collision Energy	Number of spectra for identification	Delta MS (ppm)
[illegible]	913.3571	1824.8676	2	53.1715		-0.1679
	1457.6534	2913.3192	2	86.6457		-0.0270
	484.7504	967.5815	2	26.8121	11	-0.0953
	533.7271	1065.5455	2	29.8242		-0.1059
	543.0157	2168.1702	4	26.0379		-0.1366
	576.2602	1150.6135	2	32.4400		-0.1076
	609.7636	1217.6292	2	34.5005		-0.1165
**337**	651.2865	1300.6986	2	37.0541		-0.1401
	673.8015	1345.7136	2	38.4388		-0.1252
	692.9536	2076.0310	3	36.5772		-0.1921
	734.7746	1467.6783	2	42.1886		-0.1437
	815.8816	1629.9090	2	47.1767		-0.1603
	1020.8964	2039.9721	2	59.7851		-0.1940
	682.7589	1363.6521	2	38.9897	3	-0.1488
**338**	712.3315	1422.7732	2	40.8084		-0.1246
	900.3878	1798.9254	2	52.3738		-0.1644
	524.2441	1046.5760	2	29.2410	17	-0.1025
	526.7906	1051.6754	2	29.3976		-0.1086
	573.2265	1144.5513	2	32.2534		-0.1128
	606.6129	1816.9835	3	31.3968		-0.1667
	635.9528	1904.9778	3	33.1572		-0.1412
	647.6153	1939.9891	3	33.8569		-0.1649
	664.7921	1327.7096	2	37.8847		-0.1399
	724.3059	1446.7388	2	41.5448		-0.1416
**339**	726.2987	1450.7304	2	41.6674		-0.1475
	730.8444	1459.8075	2	41.9469		-0.1332
	730.8470	1459.8147	2	41.9471		-0.1353
	733.3839	1464.9028	2	42.1031		-0.1496
	741.8473	1481.8453	2	42.6236		-0.1653
	825.3505	1648.8308	2	47.7591		-0.1443
	843.3175	1684.8091	2	48.8640		-0.1885
	980.8676	1959.9095	2	57.3234		-0.1888
	1031.4309	2061.0154	2	60.4330		-0.1681
	521.8962	1562.7974	3	26.3138	7	-0.1306
	599.7967	1197.6982	2	33.8875		-0.1194
	684.2943	2050.0483	3	36.0577		-0.1872
**340**	775.8342	1549.8021	2	44.7138		-0.1484
	874.3506	1746.8788	2	50.7726		-0.1922
	935.3869	1868.9407	2	54.5263		-0.1814
	977.4385	1953.0571	2	57.1125		-0.1947
	550.2753	1098.5360	2	30.8419	10	-0.1190

Spot	Precursor ion *m*/*z*	Precursor ion mass	Ion charge	Collision Energy	Number of spectra for identification	Delta MS (ppm)
**342**	686.2976	1370.5806	2	39.2073		-0.1541
	698.7996	1395.5846	2	39.9762		-0.1512
	849.3177	1696.6208	2	49.2330		-0.1888
	600.2648	1797.7724	3	31.0159		-0.1802
	608.2796	1821.8171	3	31.4968		-0.1778
	615.8943	1844.6610	3	31.9537		-0.1971
	618.5657	1852.6753	3	32.1139		-0.1950
	659.9371	1976.7896	3	34.5962		-0.1843
	936.2000	2805.5782	3	51.1720		0.0870
**343**	600.7605	1199.6398	2	57.9113	5	-0.1332
	605.7250	1209.5778	2	34.2521		-0.1423
	647.3055	1939.0751	3	33.8383		-0.1805
	714.3304	1426.8031	2	40.9313		-0.1570
	835.8649	1669.8900	2	48.4057		-0.1748
**352**	481.2160	960.5280	2	26.5948	7	-0.1106
	591.2367	1180.5975	2	33.3611		-0.1387
	689.2994	1376.7452	2	39.3919		-0.1610
	721.7973	1441.7255	2	41.3905		-0.1454
	725.7763	1449.7140	2	41.6352		-0.1760
	615.5852	1843.9217	3	31.9351		-0.1880
	960.3914	1918.9538	2	56.0641		-0.1856
**354**	553.5870	1657.9151	3	28.2152	4	-0.1759
	609.2687	1216.6564	2	34.4700		-0.1335
	621.7356	1241.5928	2	35.2367		-0.1361
	637.7918	1273.7030	2	36.2242		-0.1339
**356**	435.7220	869.5334	2	23.7969	18	-0.1039
	503.9085	1508.7835	3	25.2345		-0.0797
	512.7548	1023.6077	2	28.5344		-0.1127
	533.7314	1065.5706	2	29.8245		-0.1224
	579.2501	1734.9053	3	29.7550		-0.1770
	584.2382	1749.8897	3	30.0543		-0.1968
	596.7565	1191.6288	2	33.7005		-0.1303
	617.9346	1850.9778	3	32.0761		-0.1959
	628.5787	1882.8982	3	32.7147		-0.1839
	635.2987	1268.7201	2	36.0709		-0.1372
	658.2803	1972.0113	3	34.4968		-0.1922
	660.7964	1319.7238	2	37.6390		-0.1455
	669.2868	2005.0368	3	35.1572		-0.1983
	689.7888	1377.7140	2	39.4220		-0.1509
	783.8013	1565.7838	2	45.2038		-0.1957

Spot	Precursor ion *m/z*	Precursor ion mass	Ion charge	Collision Energy	Number of spectra for identification	Delta MS (ppm)
	786.8330	1571.8307	2	45.3902		-0.1793
	790.3128	1578.8042	2	45.6042		-0.1931
	887.3912	1772.9520	2	51.5746		-0.1842
357	514.2788	1026.6478	2	28.6281	7	-0.1048
	627.8533	1253.8111	2	35.6130		-0.1191
	642.8284	1283.7853	2	36.5339		-0.1431
	766.2941	1530.7565	2	44.1271		-0.1829
	840.3309	1678.8274	2	48.6804		-0.1803
	891.3913	1780.9571	2	51.8206		-0.1891
	893.8819	1785.9261	2	51.9737		-0.1769
358	368.8403	1103.5723	3	17.1304	26	-0.0733
	495.2393	988.5706	2	27.4572		-0.1065
	510.2255	1018.5447	2	28.3789		-0.1084
	513.2003	1024.4899	2	28.5618		-0.1039
	557.2359	1112.5713	2	31.2700		-0.1141
	561.7267	1121.5604	2	31.5462		-0.1216
	568.7390	1135.5761	2	31.9774		-0.1126
	591.7675	1181.6517	2	33.3937		-0.1313
	598.2562	1194.6285	2	33.7928		-0.1307
	617.2572	1232.6400	2	34.9613		-0.1402
	625.7686	1249.6554	2	35.4848		-0.1328
	627.2432	1878.8761	3	32.6346		-0.1683
	632.7751	1263.6711	2	35.9157		-0.1354
	645.2553	1932.9404	3	33.7153		-0.1962
	659.3045	1975.0891	3	34.5583		-0.1975
	694.8485	1387.8187	2	39.7332		-0.1363
	701.7864	1401.6987	2	40.1599		-0.1406
	708.7562	1415.6568	2	40.5885		-0.1590
	726.2937	1450.7303	2	41.6671		-0.1576
	731.1000	2920.4971	4	37.1349		-0.1262
	744.8147	1487.7773	2	42.8061		-0.1625
	751.8481	1501.8392	2	43.2387		-0.1576
	790.3392	1578.8253	2	45.6059		-0.1615
	822.8311	1643.8254	2	47.6041		-0.1778
	1254.0000	3758.8454	3	70.2400		0.1327
	1311.6000	3931.7622	3	73.6960		0.0159
	468.2145	934.5124	2	25.7952	12	-0.0980
	488.7303	975.5502	2	27.0569		-0.1040
	548.2184	1094.5542	2	30.7154		-0.1319

Spot	Precursor ion *m/z*	Precursor ion mass	Ion charge	Collision Energy	Number of spectra for identification	Delta MS (ppm)
**360**	557.7822	1113.6758	2	31.3036		-0.1260
	637.9503	1911.0142	3	33.2770		-0.1850
	680.7950	1359.7180	2	38.8689		-0.1425
	701.2976	1400.7260	2	40.1298		-0.1453
	731.3510	1460.8464	2	41.9781		-0.1590
	790.3418	1578.8293	2	45.6060		-0.1603
	794.2675	1586.6956	2	45.8475		-0.1752
	914.3793	1826.9315	2	53.2343		-0.1875
	1394.9000	4181.8133	3	78.6940		-0.1351
**362**	587.2714	1172.6401	2	33.1172	3	-0.1118
	635.9387	1904.9567	3	33.1563		-0.1626
	682.6268	2045.0429	3	35.9576		-0.1845
**363**	550.2401	1098.5921	2	30.8398	9	-0.1264
	620.2581	1857.9445	3	32.2155		-0.1920
	691.2755	2071.0004	3	36.4765		-0.1958
	742.2861	1482.7143	2	42.6506		-0.1566
	767.2978	1532.7372	2	44.1888		-0.1561
	792.3812	1582.9083	2	45.7314		-0.1604
	843.8263	1685.7784	2	48.8953		-0.1404
	921.3970	1840.9782	2	53.6659		-0.1987
	957.3662	1912.9166	2	55.8780		-0.1987
**368**	489.7123	977.4971	2	27.1173	10	-0.0869
	569.9437	1706.9580	3	29.1966		-0.1488
	711.3269	2131.1538	3	37.6796		-0.1948
	750.3592	1498.8541	2	43.1471		-0.1502
	767.3290	1532.8086	2	44.1907		-0.1652
	776.3612	1550.8569	2	44.7462		-0.1489
	783.3661	1564.8937	2	45.1770		-0.1761
	787.3480	1572.8359	2	45.4219		-0.1544
	895.8684	1789.9210	2	52.0959		-0.1987
	935.8769	1869.9369	2	54.5564		-0.1977
	436.7021	871.4763	2	23.8572	15	-0.0866
	484.2391	966.5611	2	26.7807		-0.0975
	586.5997	1756.9393	3	30.1960		-0.1621
	617.7739	1233.6605	2	34.9931		-0.1272
	717.2874	1432.7232	2	41.1132		-0.1629
	754.3105	1506.7719	2	43.3901		-0.1654
	760.8252	1519.7995	2	43.7907		-0.1636

Spot	Precursor ion *m/z*	Precursor ion mass	Ion charge	Collision Energy	Number of spectra for identification	Delta MS (ppm)
**376**	776.8128	1551.7682	2	44.7740		-0.1571
	779.8545	1557.8766	2	44.9611		-0.1822
	783.3163	1564.7885	2	45.1740		-0.1706
	786.8382	1571.8307	2	45.3905		-0.1688
	797.3670	1592.8926	2	46.0381		-0.1733
	830.8599	1659.8832	2	48.0979		-0.1780
	846.3340	1690.8427	2	49.0495		-0.1892
	911.8546	1820.8697	2	53.0791		-0.1910
**380**	473.2258	944.5331	2	26.1034	13	-0.0960
	482.2480	962.5801	2	26.6583		-0.0987
	584.2292	1749.8396	3	30.0538		-0.1739
	611.7636	1221.6506	2	34.6235		-0.1379
	626.2537	1250.6143	2	35.5146		-0.1215
	626.6522	1877.9860	3	32.5991		-0.1638
	646.2907	1290.6972	2	36.7469		-0.1303
	655.7786	1309.6812	2	37.3304		-0.1386
	751.7762	1501.7161	2	43.2342		-0.1783
	779.3445	1556.8311	2	44.9297		-0.1566
	824.8635	1647.8872	2	47.7291		-0.1746
	875.9106	1749.9876	2	50.8685		-0.1660
	968.4429	1935.0677	2	56.5592		-0.1965
**381**	535.5832	1603.8933	3	27.1350	11	-0.1657
	568.6162	1702.9981	3	29.1170		-0.1837
	577.2635	1152.6404	2	32.5017		-0.1280
	665.2969	1328.7299	2	37.9158		-0.1506
	693.6071	2077.9891	3	36.6164		-0.1896
	703.3082	1404.7613	2	40.2535		-0.1594
	747.2656	1492.6695	2	42.9568		-0.1529
	784.8270	1567.7995	2	45.2669		-0.1601
	816.8451	1631.8552	2	47.2360		-0.1796
	824.3686	1646.8992	2	47.6987		-0.1764
	889.3845	1776.9291	2	51.6971		-0.1746
**382**	599.6040	1795.9680	3	30.9762	2	-0.1777
	683.3219	2047.1062	3	35.9993		-0.1622
**385**	569.2261	1136.5502	2	32.0074	10	-0.1126
	600.7628	1199.5823	2	33.9469		-0.0711
	628.2899	1254.6932	2	35.6398		-0.1279
	647.7584	1293.6540	2	36.8371		-0.1518
	664.7704	1327.6772	2	37.8834		-0.1511
	736.3063	1470.7566	2	42.2828		-0.1584

Spot	Precursor ion *m*/*z*	Precursor ion mass	Ion charge	Collision Energy	Number of spectra for identification	Delta MS (ppm)
	802.8300	1603.8304	2	46.3740		-0.1849
	867.3437	1732.8672	2	50.3416		-0.1944
	906.3419	1810.8559	2	52.7400		-0.1866
	1014.3000	4053.0365	4	53.8437		0.1344
**387**	511.8559	1532.7372	3	25.7114	10	-0.1914
	550.2467	1098.5921	2	30.8402		-0.1132
	620.2721	1857.9445	3	32.2163		-0.1500
	629.2552	1884.9105	3	32.7553		-0.1667
	690.9480	2070.0164	3	36.4569		-0.1943
	742.2912	1482.7143	2	42.6509		-0.1464
	754.3465	2260.2103	3	40.2608		-0.1925
	792.3839	1582.9083	2	45.7316		-0.1551
	921.4027	1840.9782	2	53.6663		-0.1873
	957.3778	1912.9166	2	55.8787		-0.1755
**388**	633.7604	1265.6404	2	35.9763	3	-0.1343
	758.3315	1514.8052	2	43.6374		-0.1567
	763.8846	1525.9133	2	43.9789		-0.1586
**389**						
**392**	520.8034	1039.7005	2	29.0294	11	-0.1083
	650.7990	1299.7286	2	37.0241		-0.1451
	656.8104	1311.7510	2	37.3938		-0.1448
	679.8032	1357.7493	2	38.8079		-0.1576
	688.8150	1375.7612	2	39.3621		-0.1457
	733.3027	1464.7613	2	42.0981		-0.1705
	738.3207	1474.7932	2	42.4067		-0.1664
	770.2897	1538.7252	2	44.3728		-0.1605
	772.3525	1542.8657	2	44.4997		-0.1752
	788.3655	1574.8919	2	45.4845		-0.1755
	905.9105	1809.9910	2	52.7135		-0.1844
**394**	558.3257	1114.6499	2	31.3370	5	-0.0130
	586.9574	1757.8485	3	30.2174		0.0019
	737.4193	1472.8351	2	42.3513		-0.0111
	804.4092	1606.8355	2	46.4712		-0.0316
	825.3768	2473.1947	3	44.5226		-0.0863
**395**	542.7800	1083.6540	2	30.3810	8	-0.1086
	575.5971	1723.9396	3	29.5358		-0.1701
	597.5897	1789.9284	3	30.8554		-0.1812
	623.2897	1244.7016	2	35.3323		-0.1368
	646.8623	1291.8228	2	36.7820		-0.1127

Spot	Precursor ion *m*/*z*	Precursor ion mass	Ion charge	Collision Energy	Number of spectra for identification	Delta MS (ppm)
	656.7360	1311.5918	2	37.3893		-0.1344
	736.3292	1470.6439	2	42.2842		-0.1643
	907.3715	1812.9145	2	52.8033		-0.1862
	532.5981	1594.9195	3	26.9559	7	-0.1470
	536.7869	1071.5924	2	30.0124		-0.0332
	708.0133	2121.1794	3			-0.1613
**398**	714.8236	1427.7872	2	40.9617		-0.1545
	717.6361	2150.0830	3	38.0582		-0.1965
	778.8635	1555.8821	2	44.9001		-0.1697
	859.3487	1716.8822	2	49.8499		-0.1993
	676.7880	1351.7136	2	38.6225	3	-0.1521
**399**	864.3836	1726.9288	2	50.1596		-0.1762
	900.3372	1798.8414	2	52.3707		-0.1816
	437.1642	1308.6422	3	21.2299	7	-0.1715
	501.8648	1502.7518	3	25.1119		-0.1792
	519.8974	1556.8562	3	26.1938		-0.1858
**403**	550.2313	1098.5921	2	30.8392		-0.1440
	656.7408	1311.6347	2	37.3896		-0.1677
	681.8000	2723.3449	4	34.2262		-0.1740
	770.3131	1538.7980	2	44.3743		-0.1865

Table S2: Spot, Precursor ion m/z, Precursor ion mass, Ion charge, Collision Energy, Number of spectra for identification, Delta MS (ppm), Sequence, Modification, Peptide score*, Protein, Mr (kDa) / pI Theor, Mr (kDa) / pI Observed, AC number (gi NCBI) and reference organism

*Ions score is -10*Log(P), where P is the probability that the observed match is a random event.
Individual ions scores > for example "51" indicate identity or extensive homology (p<0.05).
Protein scores are derived from ions scores as a non-probabilistic basis for ranking protein hits.
Ion scores in red indicate identity or extensive homology accordingly to MASCOT search engine (p<0,05)

Spot	Sequence
1	ASDTGSYLGK<br>SGGVIQQATEQVKSAAAGAK<br>SGGVIQQATEQVK
4	VDWRETPDAHEIVVDVPGMR<br>FRLPENADLDSVAASLDSGVLTVR<br>ETPDAHEIVVDVPGMR<br>RFRLPENADLDSVAASLDSGVLTVR<br>GLDEAAVSDVGLLAADPFR<br>LPENADLDSVAASLDSGVLTVR
5	VSFIVDDAADSVKPVTYVK<br>GGKVSFIVDDAADSVKPVTYVK<br>QLSHAATAASTLFQR
6	
9	KPPGFAFIDFDDRR<br>VTSGELEDEFRVFGVLR
11	
12	VQASIGANTWVVSGTPQTKK<br>LQDLLPSIINQLGPDNLDNLR<br>KLQDLLPSIINQLGPDNLDNLR<br>LQDLLPSIINQLGPDNLDNLRR<br>VQASIGANTWVVSGTPQTK<br>IGVNTIPGIEEVNIFKDDVVIQFVNPK
13	IDWKETPEAHVFK<br>ETPEAHVFKADLPGVK<br>SIVPSATSTNSETAAFASAR
15	VFLAGADNVLQKLDR<br>VFLAGADNVLQK<br>LSPGTAFVVPAGHPFVAVASR
17	ATLGDVLANATAR<br>MGAVGHDQATDATAVQGVTVSETRVPGGGR<br>MGAVGHDQATDATAVQGVTVSETR<br>DITKVTIGEALEATALAAGDAPVER<br>IVTEFVAGQAVGQYLAR<br>VTIGEALEATALAAGDAPVER

Spot	Sequence
	LVADKPVESADALGVAGAENR
21	LQQAIAASILR<br>FEYPTIQSYR<br>FEYPTIQSYRLQQAIAASILR<br>SLALTVQQPYALLQQPSLVNLYLQR
24	FYSLPALNDPR<br>LGSDANKINPLVPVDLVIDHSVQVDVAR<br>INPLVPVDLVIDHSVQVDVAR<br>VLLQDFTGVPAVVDLAAMRDAMAK<br>VLLQDFTGVPAVVDLAAMR
26	VFLAGADNVLQK<br>LSPGTAFVVPAGHPFVAVASR<br>VFLAGADNVLQKLDR
29	IFYILWSPSSAK<br>YAIYDFDFVTAEDVQKSR<br>SGLNGIQVELQATDASEISLDEIKDR<br>YAIYDFDFVTAEDVQK
36	VLPSIGNEVLK<br>DLQMVSLTLR<br>AVVAQFNADQLLTERPHVSALVR<br>TMSEGTHLLVPILQKPFIFDIR<br>IEAAKEIASVLSR<br>LISEATTTAGNGLIELR<br>VLSRPDVEHLPDIFTSLGLEYDEK
38	IEKLSEVNPMLGFR<br>AVRQMIMAPTLELR<br>VNDAEKLVTIGGHVLR<br>LKVLANADTPDDALTAR<br>QVAEKVFANVGK<br>LSEVNPMLGFR<br>MVEPGHLDQLLHPQFENPSAYK<br>QMIMAPTLELR<br>SDFEGIFRAMDGLPVTIR<br>AETSPEDVGGMHAAVGILTER<br>QLELAVLAVFNSWESPRAK<br>KLYGEFLVNAQGEDVVAGIR<br>QQALDRLLPYQR<br>IAVDMVNEGLVEPR<br>DDVGKFIPVYLAQGILQHDPFEVLDQR

Spot	Sequence
	LGISYPELTEMQAR
	FLDMFGNVVMDIPR
	AETSPEDVGGMHAAVGILTERGGMTSHAAVVAR
	QPLSPPALSGDLGTFMAWVDDVR
	FIPVYLAQGILQHDPFEVLDQR
	QPLSPPALSGDLGTFMAWVDDVRK
	EGEWLSLNGSTGEVILGK
	QLELAVLAVFNSWESPR
	SGAAVSMPGMMDTVLNLGLNDEVAAGLAAK
	LYGEFLVNAQGEDVVAGIR
	AALVADEIAEQAEFFSFGTNDLTQMTFGYSR
	AALVADEIAEQAEFFSFGTNDLTQMTFGYSRDDVGK
	AIFEAAIAMTNQGVQVFPEIMVPLVGTPQELGHQVTLIR
**40**	
	IGTMIEIPR
	LKVLANADTPEDALAAR
	QMIMAPTVELR
	VGICGEHGGEPSSVAFFAK
	SDFEGIFRAMDGLSVTIR
	AETSPEDVGGMHAAAGILTER
	KLYGEFLINAQGEDVVAGIR
	QQALDRLLPYQR
**43**	LLDPPLHEFLPEGNVEEIVR
	LGISYPELTEMQAR
	AETSPEDVGGMHAAAGILTERGGMTSHAAVVAR
	FIPIYLAQGILQHDPFEVLDQR
	SGAAVSMPGMMDTVLNLGLNDEVAAGLAAK
	LYGEFLINAQGEDVVAGIR
	DDVGKFIPIYLAQGILQHDPFEVLDQR
	AALVADQIAEQAEFFSFGTNDLTQMTFGYSR
	AALVADQIAEQAEFFSFGTNDLTQMTFGYSRDDVGK
	NPESFLSSFSK
	VFLAGADNVLQK
	SYTIKQGHVFVAPAGAVTYLANTDGR
	LSPGTAFVVPAGHPFVAVASR
**44**	GPYSLLDQRPSIANQHGQLYEADAR
	ILHTISVPGEFQFFFGPGGR
	QGHVFVAPAGAVTYLANTDGRK
	VFLAGADNVLQKLDR

Spot	Sequence
	ILHTISVPGEFQFFFGPGGRNPESFLSSFSK
45	DLQMVNLTLR<br>MLLGLNATGFGR-<br>VAAGLGAAASLASASLYTVDGGER<br>LISEATAMAGTGLIELR
49	IRGVFGFGI-<br>FGSWPDGLYSSVR<br>LVFDEPESGVTIIK<br>LSSLLAGLTILDGEGGLSLR
50	VAQDAVIAFK<br>VMPQGFGATIR<br>DIGFNGLADPNR<br>VAQDAVIAFKTALWFWMNNVHR<br>EIAAFFAHVTHETGHFCYISEINK<br>SAFLSAVNAYPGFAHGGTEVEGK<br>DIGFNGLADPNRVAQDAVIAFK<br>TALWFWMNNVHR<br>SAFLSAVNAYPGFAHGGTEVEGKR<br>GPLQISWNYNYGPAGR<br>SGGGGGGGGGGGGGSGGANVANVVTDAFFNGIK
51	VFLAGADNVLQKLDR<br>VFLAGADNVLQK<br>LSPGTAFVVPAGHPFVAVASR
	TIGYKVGTMIEIPR<br>IEKLSEVNPMLGFR<br>AVRQMIMAPTLELR<br>VNDAEKLVTIGGHVLR<br>LKVLANADTPDDALTAR<br>QVAEKVFANVGK<br>MVEPGHLDQLLHPQFENPSAYK<br>QMIMAPTLELR<br>GVGELVKFATER<br>SDFEGIFRAMDGLPVTIR<br>KQLELAVLAVFNSWESPR<br>AETSPEDVGGMHAAVGILTER<br>QLELAVLAVFNSWESPRAK<br>KLYGEFLVNAQGEDVVAGIR

Spot	Sequence
	QQALDRLLPYQR
	IAVDMVNEGLVEPR
52	DDVGKFIPVYLAQGILQHDPFEVLDQR
	LGISYPELTEMQAR
	FLDMFGNVVMDIPRSLFEEK
	FLDMFGNVVMDIPR
	AETSPEDVGGMHAAVGILTERGGMTSHAAVVAR
	QPLSPPALSGDLGTFMAWVDDVR
	FIPVYLAQGILQHDPFEVLDQR
	QPLSPPALSGDLGTFMAWVDDVRK
	AAILVRAETSPEDVGGMHAAVGILTER
	EGEWLSLNGSTGEVILGK
	SAVKIAVDMVNEGLVEPR
	QLELAVLAVFNSWESPR
	SGAAVSMPGMMDTVLNLGLNDEVAAGLAAK
	LYGEFLVNAQGEDVVAGIR
	AIFEAAIAMTNQGVQVFPEIMVPLVGTPQELGHQVTLIR
	SGAAVSMPGMMDTVLNLGLNDEVAAGLAAKSGER
	AALVADEIAEQAEFFSFGTNDLTQMTFGYSR
	AALVADEIAEQAEFFSFGTNDLTQMTFGYSRDDVGK
	IEKLSEVNPMLGFR
	LSEVNPMLGFR
	MVEPGHLDQLLHPQFENPSAYK
	KLYGEFLVNAQGEDVVAGIR
	QQALDRLLPYQR
	LGISYPELTEMQAR
53	EGEWLSLNGSTGEVILGK
	QLELAVLAVFNSWESPR
	SGAAVSMPGMMDTVLNLGLNDEVAAGLAAK
	LYGEFLVNAQGEDVVAGIR
	AIFEAAIAMTNQGVQVFPEIMVPLVGTPQELGHQVTLIR
	AALVADEIAEQAEFFSFGTNDLTQMTFGYSR
	LVTVEDIVR
	FFEVPTGWK
	TIFDFEAIKK
	DSQDALAPLVDVALKLSK
	SMPTSAALDVVAK
54	RVWVGQNSLMSTPAVSAVIR
	DSQDALAPLVDVALK

Spot	Sequence
	FFVTPSDSVAIIAANAVQSIPYFASGLK
	ELMANLVSMQSSLSDVNK
	VWVGQNSLMSTPAVSAVIR
	FFVTPSDSVAIIAANAVQSIPYFASGLKGVAR
	SKFESLVHDLIER
	VQEVVSEIFGKNPSK
	VQEVVSEIFGK
	EVDEVLLVGGMTR
	QYSPSQVGAFVLTK
55	AVITVPAYFNDAQR
	QAVTNPQNTFFGTKR
	SQVFSTAADNQTQVGIR
	IINEPTAAALSYGMNNK
	ATNGDTFLGGEDFDNTLLEFLVSDFKK
	WKIVPW-
	DGTNIVLWK
	DGNVVLAPANPRDEHQHWYK
	MVNNIYLNFDAFHGDKDHGGVR
	MVNNIYLNFDAFHGDK
	GHGGVHDGTTVVLWEWAK
59	ILPWGDEAYAAGGSSAANAPR
	FSTQVKDEEGNPAFALVNK
	HSNSVKDEEGYPAFALVNR
	DEEGYPAFALVNR
	LVPFNPEYQDESVLWTESGDVGK
	LVPFNPEYQDESVLWTESGDVGKGFR
	LVPYNPGYQDESVLWTESR
	LVPFNPEYQDESVLWTESGDVGK
	LTLPLTK
	VGLTVDLPVVGK
	VPYDFLVSLAK
	VGLTVDLPVVGKLTLPLTK
	NPYSHAIPVCEVTYTLR
	SGELKLPTLSSIF-
	GFVADKLANIQKPEAELADVTVGHVGR
61	LANIQKPEAELADVTVGHVGR
	LANIQKPEAELADVTVGHVGRDGATLAGR
	DAGRDWDIDYEMR

Spot	Sequence
	VDVRNPYSHAIPVCEVTYTLR<br>GFVADKLANIQKPEAELADVTVGHVGRDGATLAGR<br>LDVPVKVPYDFLVSLAK<br>TVASGTVPDPGSLAGDGATTRLDVPVKVPYDFLVSLAK<br>TVASGTVPDPGSLAGDGATTR
**64**	LQQAIATSNLPLSPLFFQQSPALSLVQSLVQTIR
**66**	YAGSEFKGADGTAYIVLR<br>VAEAEDKTPGGLILTETTK<br>HLILKEDDIIGILETDDVK<br>IAVDIETGAQVVYSK<br>TVGGILLPSTAQTKPQGGEVVAVGAGR
**67**	TIFDFEAIKK<br>YFSKDAVQIITK<br>LVTVEDIVR<br>TIFDFEAIK<br>LVFRLSGTGSVGATIR<br>FFEVPTGWK<br>DNLGGDKLVTVEDIVR<br>TGRDSQEALAPLVDVALK<br>DSQEALAPLVDVALKLSK<br>SMPTSAALDVVAK<br>ELMANLVSMQSSLSDVNK<br>RVWVGQNSLMSTPAVSAVIR<br>ATGAFILTASHNPGGPTEDFGIK<br>ELMANLVSMQSSLSDVNKLIK<br>SDVSEVVAADEFEYKDPVDGSVSK<br>VWVGQNSLMSTPAVSAVIR<br>RFFVTPSDSVAIIAANAVQSIPYFASGLK
**69**	TLLDYTATK<br>VFRTNIFSYFLVSK<br>EGATVAFTFVR<br>ESIGDVTEADLERVFR<br>TLLDYTATKGAIVAFTR<br>TNIFSYFLVSKHAVPR<br>TNIFSYFLVSK<br>ESIGDVTEADLER<br>MEPGACIINTSSVNAYKGNK<br>VNGVAPGPVWTPLIPASFGKEK

Spot	Sequence
	GIRVNGVAPGPVWTPLIPASFGK
	VASAYGGRIDVVVNNAAEQYER
	IDVVVNNAAEQYER
	MEPGACIINTSSVNAYK
	VNGVAPGPVWTPLIPASFGK
	AAQPAEIAPSFVFLASNQDSSYMSGQILHVNGGVIVNS.-
	SDFEGIFR
	IGTMIEIPR
	AMDGLSVTIR
	LKVLANADTPEDALAAR
	QMIMAPTVELR
	AETSPEDVGGMHAAAGILTER
74	KLYGEFPINAQGEDVVAGIR
	VLANADTPEDALAAR
	LLDPPLHEFLPEGNVEEIVR
	DDVGKFIPIYLAQGILQHDPFEVLDQR
	FIPIYLAQGILQHDPFEVLDQR
	LYGEFPINAQGEDVVAGIR
	AALVADQIAEQAEFFSFGTNDLTQMTFGYSR
	GWPTEFAAYKVAK
	GWPTEFAAYK
	LDELLSTFLR
78	VVNVSSDFGLLR.Y
	NVTEALLPLLLQASSSGGGR
	QTGLLTPAQGAANVVK
	VALLPEGGPTGAFFALGK
	VALLPEGGPTGAFFALGKEAPFV-
	WKIVPW-
	MVNNIYLNFDAFHGDKDHGGVR
	FSTQVKDEEGNPAFALVNK
	DEEGNPAFALVNK
83	HSNSVKDEEGYPAFALVNR
	DEEGYPAFALVNR
	LVPFNPEYQDESVLWTESGDVGK
	LVPFNPEYQDESVLWTESGDVGKGFR
	ILPWGDEAYAAGGSSAANAPR

Spot	Sequence
	LVPYNPGYQDESVLWTESR
	LLEDSLAEKMLAGEVK
	IGFDLDYDEKDTSYNR
	NPNRPIASFIFSGPTGVGK
	RLGHNFVGTEQILLGLIGEGTGIAAK
	QLGHNYIGSEHLLLGLLR
86	NTLLIMTSNVGSSVIEKGGR
	VITLDMGLLVAGTK
	FQPVKVPEPTVDETIQILR
	GSGFVAVEIPFTPR
	LIGSPPGYVGYTEGGQLTEAVRR
	LGHNFVGTEQILLGLIGEGTGIAAK
	ALAAYYFGSEEAMIR
	NTLLIMTSNVGSSVIEK
87	LIALDMGALIAGAK
	VIQQYVENELAK
	SDFEGIFR
	IGTMIEIPR
	AMDGLSVTIR
	LKVLANADTPEDALAAR
	QMIMAPTVELR
96	AETSPEDVGGMHAAAGILTER
	KLYGEFPINAQGEDVVAGIR
	VLANADTPEDALAAR
	LLDPPLHEFLPEGNVEEIVR
	DDVGKFIPIYLAQGILQHDPFEVLDQR
	FIPIYLAQGILQHDPFEVLDQR
	LYGEFPINAQGEDVVAGIR
	AIFEAAIAMSNQGVEVFPEIMVPLVGLPQELGHQVNVIK
	GFPRPLLDISAANFGK
104	KLLAGLLAVESAQDAVIR
	VPSYAGGVAEITAR
	QQALDRLLPYQR
	TIGYKIGTMIEIPR
	LLPYQRSDFEGIFR
	LKVLANADTPEDALAAR

Spot	Sequence
107	QMIMAPTVELR<br>SDFEGIFRAMDGLSVTIR<br>AETSPEDVGGMHAAAGILTER<br>KLYGEFPINAQGEDVVAGIR<br>QQALDRLLPYQR<br>VLANADTPEDALAAR<br>LLDPPLHEFLPEGNVEEIVR<br>DDVGKFIPIYLAQGILQHDPFEVLDQR<br>LGISYPELTEMQAR<br>FIPIYLAQGILQHDPFEVLDQR<br>AALVADQIAEQAEFFSFGTNDLTQMTFGYSRDDVGK<br>LYGEFPINAQGEDVVAGIR<br>AALVADQIAEQAEFFSFGTNDLTQMTFGYSR
108	LWGENFFDPATKK<br>LYMEARPLEEGLAEAIDDGRIGPR<br>GHVFEEMQRPGTPLYNIK<br>LWGENFFDPATK<br>STLTDSLVAAAGIIAQEVAGDVR<br>LYMEARPLEEGLAEAIDDGR<br>GVQYLNEIKDSVVAGFQWASK<br>YRVENLYEGPLDDVYATAIR<br>DLQEDFMGGAEIIVSPPVVSFR<br>NMSVIAHVDHGKSTLTDSLVAAAGIIAQEVAGDVR<br>DGNEYLINLIDSPGHVDFSSEVTAALR<br>LLEPVYLVEIQAPENALGGIYGVLNQK<br>VENLYEGPLDDVYATAIR<br>LLEPVYLVEIQAPENALGGIYGVLNQKR
111	GLIGDIISR<br>IIGATRPWEAAPGTIR<br>GLIGDIISRFEK
112	LSSINVENVEENRR<br>LSTKKPWSLSFSFGR<br>ANSEATLGTYKGDAAADTESLHVK<br>KPWSLSFSFGR<br>VTPEVIAEYTVR<br>ALNEHHVLLEGTLLKPNMVTPGSDSK<br>KVTPEVIAEYTVR

Spot	Sequence
	NAAYIGTPGKGILAADESTGTIGK<br>AVLKIGPNEPSQLAIDLNAQGLAR<br>IGPNEPSQLAIDLNAQGLAR<br>TVPAAVPAVVFLSGGQSEEEATRNLNAMNK<br>TVPAAVPAVVFLSGGQSEEEATR
114	AFRDTIDLFVER<br>DTIDLFVER<br>DKLGDRPVFVLVEADA-<br>KLPGEVISEEYSLEYGTDK<br>GFIFGPPIALAIGAK<br>VIPDFPKPGIMFQDITTLLLDPKAFR<br>LGDRPVFVLVEADA-<br>VIPDFPKPGIMFQDITTLLLDPK
134	FGFLKWGSSAFR<br>YYSLPALSDPR<br>LTGRSDDTVAMVESYLR<br>NGVTATDLVLTVTQMLRK<br>YTVHLPTNVSEIKPGQDVTVTTDNGK<br>SDDTVAMVESYLR<br>LGSDPNKINPLVPVDLVIDHSVQVDVAR<br>INPLVPVDLVIDHSVQVDVAR<br>DVWPSTEEIAEVVK<br>NMLVVPPGSGIVHQVNLEYLAR<br>NGVTATDLVLTVTQMLR<br>FVEFYGQGMSELSLADR<br>ANYLASPPLVVAYALAGTVNIDFEKEPIGISK
136	FGHVTFFWNGNR<br>LDQLQLLLKGVSER<br>LDQLQLLLK<br>GWDAQVLGEAPYKFK<br>ALEYADFDNFDRVR<br>YLVSPPEIDR<br>YAGMLQYDGELKLPSR<br>IQILTSHTLQPVPVAIGGPGLHPGVK<br>AHGTAVGLPSDDDMGNSEVGHNALGAGR<br>YLVSPPEIDRTSGEYLVK<br>GWDAQVLGEAPYK<br>ALEYADFDNFDR<br>IILDAVEQVGGIYLVTADHGNAEDMVKR<br>VHILTDGRDVLDGSSIGFVETLENDLLELR

Spot	Sequence
	IILDAVEQVGGIYLVTADHGNAEDMVK<br>SGYFDATKEEYVEVPSDSGITFNVAPNMK
139	DDLRLPTDETLVAQIK<br>LPTDETLVAQIK
145	YLKPAVAGFLMQK<br>IGVIESLLAK<br>TLPGVLALDDA-<br>AGLADKMSHISTGGGASLELLEGK<br>VFVRADLNVPLDDAQK<br>ELDYLVGAVANPKKPFAAIVGGSK<br>SLVEEDKLELATSLIEK<br>LASVADLYVNDAFGTAHR<br>ADLNVPLDDAQKITDDTR<br>KLASVADLYVNDAFGTAHR<br>ELDYLVGAVANPK<br>GVTTIIGGGDSVAAVEKAGLADK<br>YLKPAVAGFLMQKELDYLVGAVANPK<br>GVSLLLPTDIVVADK<br>GVSLLLPTDIVVADKFAADAESK<br>LAELTTTKGVTTIIGGGDSVAAVEK<br>LAAALPEGGVLLLENVR<br>TVIWNGPMGVFEFQK<br>TVIWNGPMGVFEFQKFAAGTEAIAK<br>IVPATAIPDDWMGLDVGPDATK
147	LYIQTIDLDHEDKFDFDPLDVTK<br>GPILLEDYHLIEK<br>LGPNYLMLPVNAPK<br>DLYDSIAAGNYPEWK<br>EGNFDLVGNNMPVFFIR
148	FLPDKAIDLIDEAGSR<br>NPNRPIASFIFAGPTGVGK<br>GNGFVAVEIPFTPR<br>VGLKNPNRPIASFIFAGPTGVGK<br>LIGSPPGYVGYTEGGQLTEAVRR<br>LGHNFVGTEQILLGLIGEGTGIAAK<br>ALAAYYFGSEEAMIR
	VILFIDEIHLVLGAGR<br>VVGQTEAVSAVAEAVLRSR<br>AGLGRPQQPTGSFLFLGPTGVGKTELAK

Spot	Sequence
149	TVDFRNTVIIMTSNLGAEHLLAGMVGK<br>AGLGRPQQPTGSFLFLGPTGVGK<br>RFQQVFVAEPSVPDTVSILR<br>NTVIIMTSNLGAEHLLAGMVGK<br>HFRPELLNRLDEIVIFDPLSHEQLR<br>NNPVLIGEPGVGKTAVVEGLAQR<br>GIALAVTDAALDIILSLSYDPVYGARPIRR<br>VVGQTEAVSAVAEAVLR<br>ALAEQLFDDENLLVRIDMSEYMEQHSVAR<br>AHVAVFNTLLQVLDDGRLTDGQGR<br>ALAEQLFDDENLLVR<br>FQQVFVAEPSVPDTVSILR
154	VELPPNPEDALEVFVDGHAVRIPK<br>ALSEVAGALLPYDSLSAVRDR<br>VELPPNPEDALEVFVDGHAVR<br>AVTWRDALAVVAEVLHQVKPEEITGVAGK<br>NWELKGTETIDVTDAVGSNIR<br>VSSTPFKTVVENFYMTDAITR<br>DALAVVAEVLHQVKPEEITGVAGK<br>FATEVAGVQDLGMLGR<br>SNYLMNTSIAGLEKADVFLLVGTQPR<br>ALSEVAGALLPYDSLSAVR
163	AGIALNGNFVK<br>SSIFDAKAGIALNGNFVK<br>VPTVDVSVVDLTVRLEK<br>FGIVEGLMTTVHAITATQK<br>VVISAPSKDAPMFVVGVNEK<br>AASFNIIPSSTGAAK<br>VPTVDVSVVDLTVR<br>LTGMSFRVPTVDVSVVDLTVR<br>LVSWYDNEWGYSTRVVDLIR<br>VINDKFGIVEGLMTTVHAITATQK<br>LVSWYDNEWGYSTR<br>AGIALNGNFVKLVSWYDNEWGYSTR<br>AEAEGSLKGILGYVEEDLVSTDFQGDSR<br>GILGYVEEDLVSTDFQGDSR
164	FFEVPTGWK<br>TIFDFEAIKK<br>RVWVGQNSLMSTPAVSAVIR<br>ATGAFILTASHNPGGPTEDFGIK<br>DSQEALAPLVDVALK

Spot	Sequence
	SDVSEVVAADEFEYKDPVDGSVSK
	VWVGQNSLMSTPAVSAVIR
	RFFVTPSDSVAIIAANAVQSIPYFASGLK
	FFVTPSDSVAIIAANAVQSIPYFASGLKGVAR
	SRIFYILWSPSSAK
	IFYILWSPSSAK
**170**	YAIYDFDFVTAEDVQKSR
	SGLNGIQVELQATDASEISLDEIKDR
	YAIYDFDFVTAEDVQK
	IASFVDPDGWK
	VVLVDNTDFLK
	SAEAVDLATKELGGK
**172**	ILRQPGPLPGINTK
	VVLVDNTDFLKELH.-
	IASFVDPDGWKVVLVDNTDFLK
	GNAYAQVAIGTNDVYK
	EPGPVKGGSTVIAFAQDPDGYMFELIQR
	NFFKVTTTDDPVIR
	DQYNHSILSVIGYKPR
	SVQSWDIVIQR
	WKLDEETSLVAR
	WTAQALLAGADMMK
	QFLTLNALNEFDPKVTGVDWR
**174**	QFLTLNALNEFDPK
	NGEKVTFDEPNPFATEGEEAASVAYR
	DFAAQINLNTANMWGIVK
	EDINSAHSLAVEATYVNQNFSQQVLLR
	DGSQLDLLTVNETAQEALPEAK
	VTFDEPNPFATEGEEAASVAYR
	DAVFDFAGLEDSIGLASADDSSFR
**175**	
	LYMEARPLEEGLAEAIDDGRIGPR
	LWGENFFDPATK
	STLTDSLVAAAGIIAQEVAGDVR
	GVQYLNEIKDSVVAGFQWASK
	YRVENLYEGPLDDVYATAIR
**178**	NMSVIAHVDHGKSTLTDSLVAAAGIIAQEVAGDVR
	STLTDSLVAAAGIIAQEVAGDVRMTDTR
	DGNEYLINLIDSPGHVDFSSEVTAALR
	LLEPVYLVEIQAPENALGGIYGVLNQK
	VENLYEGPLDDVYATAIR

Spot	Sequence
	LLEPVYLVEIQAPENALGGIYGVLNQKR<br>DLQEDFMGGAEIIVSPPVVSFR
179	VVVDAMGLLLPR<br>LTSHKFPVLNLVQMSAVR<br>FLSEALGVMHVAEKLQSR<br>FLLAGGFSKGQPHFAENIFK<br>AGQLLIVPQGYLVATK<br>AQGEGFQYIAFETNPDTMVSHVAGK<br>NSVLSDLPAAVIASSYAISMEEAAELK<br>VDLYQDAIMSPFWNFNAHSAMYGIR<br>NSVLSDLPAAVIASSYAISMEEAAELKNGR<br>AGQLLIVPQGYLVATKAQGEGFQYIAFETNPDTMVSHVAGK
185	SDETVAMIEAYLR<br>VLLQDFTGVPAVVDLAAMR
186	LGSDANKINPLVPVDLVIDHSVQVDVAR<br>SDETVAMIEAYLR<br>VLLQDFTGVPAVVDLAAMR
188	LSPGTAFVVPAGHPFVAVASR<br>GPYSLLDQRPSIANQHGQLYEADAR<br>VFLAGADNVLQKLDR<br>QGHVFVAPAGAVTYLANTDGR
189	VVDMYKEAFESIEIPK<br>ISTMTADDYFEKYPELR<br>FSQEPQPIDWEYYR
192	FALESFWDGK<br>SEHAFYLDWAVHSFR<br>ISEEEYISAIKEEISK<br>ALGIDTVPVLVGPVSYLLLSKPAK<br>SFSLLSLLGSILPIYKEVVAELK<br>AAGASWIQFDEPTLVK<br>SFSLLSLLGSILPIYK<br>GMLTGPVTILNWSFVR<br>TLTSLSGVTAYGFDLIR<br>EVEDLEAAGIQVIQIDEAALR
	AGIALNGNFVK<br>SSIFDAKAGIALNGNFVK<br>VPTVDVSVVDLTVRLEK<br>DAPMFVVGVNEK<br>AASFNIIPSSTGAAK

Spot	Sequence
196	VPTVDVSVVDLTVR<br>LTGMSFRVPTVDVSVVDLTVR<br>VLPVLNGKLTGMSFR<br>VINDKFGIVEGLMTTVHAITATQK<br>DAPMFVVGVNEKEYK<br>TLLFGEKEVAVFGCR<br>LVSWYDNEWGYSTR<br>VPTVDVSVVDLTVRLEK<br>AEAEGSLKGILGYVEEDLVSTDFQGDSR<br>VVISAPSKDAPMFVVGVNEK<br>VALQSDDVELVAVNDPFISTDYMTYMFK<br>VALQSDDVELVAVNDPFISTDYMTYMFKYDTVHGQWK<br>GILGYVEEDLVSTDFQGDSR
197	AHGGVHDGTEIVLWK<br>ATGLAIKHSLGQSHPVK<br>MVNNIYLNFDAFHGDK<br>ILPWGPEANSSAAHAGGPHAVR<br>VKDEEGMPAFALVNK<br>LAPFSPDQEDASVLWTESKDVGK<br>LAPFSPDQEDASVLWTESK<br>LVPYNPDYQDESVLWTESR
200	
203	
205	IGINGFGR<br>IKIGINGFGR<br>AGIALNDHFIK<br>VPTVDVSVVDLTVRIEK<br>AASFNIIPSSTGAAK<br>VPTVDVSVVDLTVR<br>LTGMSFRVPTVDVSVVDLTVR<br>LVSWYDNEWGYSNRVVDLIR<br>TLLFGEKPVTVFGIR<br>VLPELNGKLTGMSFR<br>VIHDNFGIIEGLMTTVHAITATQK<br>LVSWYDNEWGYSNR<br>VPTVDVSVVDLTVRIEK<br>AASEGPLKGIMGYVEEDLVSTDFTGDSR

Spot	Sequence
	NPEEIPWGEAGAEYVVESTGVFTDKDK<br>VALQSEDVELVAVNDPFITTDYMTYMFK<br>GIMGYVEEDLVSTDFTGDSR
**206**	LPWPELK<br>TCGIGPRLPWPELK<br>ELADIPAYCR<br>-.SAGTSCVPGWAIPHNPLPSCR<br>CTALSILMDGAIPPGPDAQLEGR
**207**	APGKAFLDAVA-<br>VDIGQVLLSVR
**210**	NIPLVAFIGR<br>GVDRVFVDHPLFLER<br>SSFDFIDGYEKPVEGR<br>TGGLGDVLGGLPPAMAANGHR<br>FAFSDYPELNLPERFK<br>YDQYKDAWDTSVVSEIK<br>FKSSFDFIDGYEKPVEGR<br>FSLLCQAALEAPR<br>FAFSDYPELNLPER<br>AGILEADRVLTVSPYYAEELISGIAR<br>VLTVSPYYAEELISGIAR
**213**	LPSSPDVALHLIER<br>RHHLAFSVADYDGFVTGLK<br>LAAFYEAVLGFER<br>IPSPTYSGFQVAWLR
**214**	IASFVDPDGWK<br>VVLVDNTDFLK<br>IASFVDPDGWKVVLVDNTDFLK<br>GGSTVIAFAQDPDGYMFELIQR
**219**	FVQAGSEVSALLGR<br>QDVLLFIDNIFR<br>TVLIMELINNIAK<br>QDVLLFIDNIFR<br>YKELQDIIAILGLDELSEEDR<br>DINKQDVLLFIDNIFR<br>GFQMILSGELDNLPEQAFYLVGNIDEAAAK

Spot	Sequence
242	VFLAGADNVLQKLDR<br>NPESFLSSFSK<br>LSPGTAFVVPAGHPFVAVASR<br>GPYSLLDQRPSIANQHGQLYEADAR<br>QGHVFVAPAGAVTYLANTDGR<br>ILHTISVPGEFQFFFGPGGR<br>QGHVFVAPAGAVTYLANTDGRK
245	FFEVPTGWK<br>DSQEALAPLVDVALK<br>FFVTPSDSVAIIAANAVQSIPYFASGLK
249	LDQLQLLLK<br>GWDAQVLGEAPYKFK<br>YAGMLQYDGELKLPSR<br>GWDAQVLGEAPYKFK
251	VPTVDVSVVDLTVRLEK<br>VVISAPSKDAPMFVVGVNEK<br>VPTVDVSVVDLTVR<br>LTGMSFRVPTVDVSVVDLTVR<br>VINDKFGIVEGLMTTVHAITATQK<br>LVSWYDNEWGYSTR<br>NPEEIPWGSVGAEYVVESTGVFTDQEK<br>VALQSDDVELVAVNDPFISTDYMTYMFK<br>VALQSDDVELVAVNDPFISTDYMTYMFKYDTVHGQWK<br>GILGYVEEDLVSTDFQGDSR
252	IWHHTFYNELR<br>GYSFTTSAEREIVR<br>AVFPSIVGRPR<br>VAPEEHPVLLTEAPLNPK<br>DLTDSLMKILTER<br>KDLYGNIVLSGGSTMFPGIADR<br>LDLAGRDLTDSLMK<br>TTGIVLDSGDGVSHTVPIYEGYALPHAILR<br>LAYIALDYEQELETAKNSSSVEK<br>SYELPDGQVITIGAER<br>LAYIALDYEQELETAK
257	
261	MMDVQSVAQQLQMMMQLER
262	

Spot	Sequence
265	LADLVGVTLGPK<br>DLITILEDAIR<br>KSQYLDDIATLTGGTVIR<br>LASKVDAIIETLENDEQK<br>LSGGVAVIQVGAQTETELKEK<br>EVELEDPVENIGAK<br>TNDLAGDGTTTSVVLAQGMITEGVK<br>GGYPILIVTEDIEQEALATLVVNR<br>SQYLDDIATLTGGTVIR<br>YGYNAATGKYEDLMAAGIIDPTK
266	SRGFGFVTFSDEDAMR<br>GFGFVTFSDEDAMR
267	
268	MELVDAAFPLLK<br>IVQGLPIDEFSR<br>ELVSDDEWLNGEFITTVQQR<br>VLVVANPANTNALILK<br>GVMLGADQPVILHMLDIPPAAEALNGVK<br>VLVTGAAGQIGYALVPMIAR
272	SLVPSLPR<br>IDWKETPEAHVFK<br>TSSETAAFAGAR<br>ASMENGVLTVTVPKEEAK<br>ASMENGVLTVTVPK<br>VEVEDGNVLQISGER
273	NVRPDYLNNIWK<br>NVRPDYLNNIWKVMNWK<br>LGWAIDEDFGSFEALVKK<br>MNAEGAALQGSGWVWLALDKEAK<br>ALEQLDTAVSKGDASAVVQLQGAIK<br>GASLVPLLGIDVWEHAYYLQYK<br>LGWAIDEDFGSFEALVK
274	VEDLADEVERIR<br>EVDVVGVFR<br>DAIKVMFNL.-<br>VSPRVEDLADEVER<br>DVEEAFEVSAR<br>FGFSQRDVEEAFEVSAR<br>AGVGPETGVLVVGAGPIGLVSLLAAR<br>GAQGSDAAAAGGEVEENMAAWLVAK

Spot	Sequence
	RAGVGPETGVLVVGAGPIGLVSLLAAR
	GKGAQGSDAAAAGGEVEENMAAWLVAK
	IMPFKLPPVGPYDVR
	AGVGPETGVLVVGAGPIGLVSLLAARAFGAPR
276	SDFEGIFRAMDGLPVTIR
	KLYGEFLVNAQGEDVVAGIR
	LGISYPELTEMQAR
	FLDMFGNVVMDIPR
	SGAAVSMPGMMDTVLNLGLNDEVAAGLAAK
278	VQIVGDDLLVTNPTRVAK
	TLVLPVPAFNVINGGSHAGNK
	YNQLLRIEEELGDAAVYAGAK
	VQIVGDDLLVTNPTR
	LTDEIGQKVQIVGDDLLVTNPTR
	QIFDSRGNPTVEVDVGLSDGSYAR
	GAVPSGASTGIYEALELRDGGSDYLGK
	GAVPSGASTGIYEALELR
	LAMQEFMILPTGASSFK
	SGETEDTFIADLSVGLSTGQIK
282	SVSINEFLKPAEGER
	GPAAAAPSIEDQAQFPSLGGK-
284	NTSAVSIILR
	IADDDVILVK
	YFVEAGAIAVR
	SAKDSYLLNGYALNTGR
	VLVELAELQDR
	MQLGVQVLVTDPRELEK
	EVGDGTTSVVIIAAELLKR
	AITAPTPDILGER
	GANDYMLDEIER
	MQLGVQVLVTDPR
	DSYLLNGYALNTGR
	IIQFATEAAITILR
	NNLEYGVIEPSMSK
	VLSVNAAKDATELVAK
	EVGDGTTSVVIIAAELLK
	EQLAIAEFAESLLIIPK
286	VTSPLLEPSSVEK
	LFQVEYAIEAIK
	LFQVEYAIEAIKLGSTAIGLK
	ELTLQEAETIALSILK

Spot	Sequence
	YHLYTPAEVEAVIARL-
	LADLVGVTLGPK
	ALSYPLKLIAK
	IAAIKAPGFGER
	LQTGVNKLADLVGVTLGPK
	DLINVLEEAIR
**287**	VVAAGANPVQITR
	NAGVNGSVVTEKVLSNDNFK
	LAGGVAVIQVGAQTETELKEK
	GYISPYFVTDSEK
	EVELEDPVENIGAK
	LAGGVAVIQVGAQTETELK
	GILLGAVHGMVEALFRR
	NTVESITGIISK
	FDYILTQQAFVTVDKK
	DLFPLLPEAFK
	VSLAGHEEYIVR
	DSLVEAKSDIVVK
**292**	GGRDLFPLLPEAFK
	DLFPLLPEAFKGIK
	FYEKEGLPAFPMGK
	SDIFGERGILLGAVHGMVEALFR
	GILLGAVHGMVEALFR
	FDYILTQQAFVTVDK
	QIGVGWGSQGPAQAQNLR
	VLPVLNGKLTGMSFR
	AGIALNGNFVK
	VPTVDVSVVDLTVRLEK
	FGIVEGLMTTVHAITATQK
	VVISAPSKDAPMFVVGVNEK
	AASFNIIPSSTGAAK
	VPTVDVSVVDLTVR
**305**	LTGMSFRVPTVDVSVVDLTVR
	LVSWYDNEWGYSTRVVDLIR
	VINDKFGIVEGLMTTVHAITATQK
	LVSWYDNEWGYSTR
	SSIFDAKAGIALNGNFVK
	AGIALNGNFVKLVSWYDNEWGYSTR
	AEAEGSLKGILGYVEEDLVSTDFQGDSR

Spot	Sequence
	GILGYVEEDLVSTDFQGDSR
	ELFEFLPK
**309**	MLISMVETELEKR
	YVELTSDFVYPYR
	SNFSVVDGPASSAAGNPDEIAK
	IGVIESLLAK
	YSLKPLVPR
	KPFAAIVGGSK
	VFVRADLNVPLDDAQK
	MSHISTGGGASLELLEGK
	LASVADLYVNDAFGTAHR
	ADLNVPLDDAQK
	ADLNVPLDDAQKITDDTR
**311**	ASVPTIKFLLEK
	KLASVADLYVNDAFGTAHR
	ELDYLVGAVANPK
	YLKPAVAGFLMQK
	GVSLLLPTDIVVADK
	GVTTIIGGGDSVAAVEK
	LAAALPEGGVLLLENVR
	TVIWNGPMGVFEFQK
	SLVEEDKLELATSLIEK
	WKILPW-
	AHGGVHDGTEIVLWK
	VKDEEGMPAFALVNK
	AGGEDYSLTVR
	DGAVVLAPVNPK
**312**	MVNNIYLNFDAFHGDK
	DEEGYPAFALVNKVTGEALK
	DEEGMPAFALVNK
	ILPWGPEANSSAAHAGGPHAVR
	LAPFSPDQEDASVLWTESKDVGK
	LAPFSPDQEDASVLWTESK
	GNGTGGESIYGEKFPDEK
	HVVFGQVVEGMDVVKAIEK
	IVMELYANEVPK
	IVMELYANEVPKTAENFR
**318**	IVMELYANEVPK
	IVMELYANEVPKTAENFR
	VFFDMTVGGAPAGR
	VIPEFMCQGGDFTR

Spot	Sequence
	HVVFGQVVEGMDVVK
	GLIGDIISR
	GLIGDIISRFEK
319	IIGATRPWEAAPGTIR
	RIIGATRPWEAAPGTIR
	FRAPVEPY-
	ISGDSLKDLYK
	VQIVGDDLLVTNPTRVAK
	DGGSDYLGKGVLK
	VNQIGSVTESIEAVRMSK
	TLVLPVPAFNVINGGSHAGNK
	IEEELGDAAVYAGAK.F
	YNQLLRIEEELGDAAVYAGAK
321	IPLYQHIANLAGNK
	AVSNVNNIIGPAIVGK
	VNQIGSVTESIEAVR
	VQIVGDDLLVTNPTR
	KIPLYQHIANLAGNK
	GAVPSGASTGIYEALELR
	GNPTVEVDVGLSDGSYAR
	LAMQEFMILPTGASSFK
	SGETEDTFIADLSVGLSTGQIK
326	GNPTVEVDVGLSDGSYAR
	SGETEDTFIADLSVGLSTGQIK
	QLEVTINGIGER
328	ELLDGLYTGIDSR
	ANEFGIVLGKLSGR
	GTYEIISPDDIGLTR
329	FLDELAKIDFPGAK
	VVEAYEASPPEVK
	LDELLSTFLR
	VVNVSSDFGLLR
	NVTEALLPLLLQASSSGGGR
334	QLASNGITVVLTAR
	QTGLLTPAQGAANVVK
	VALLPEGGPTGAFFALGK
	ALDLELDLTDKGLGVR
335	ALDLELDLTDK
	GVDEILLISVNDPFVMK
	AVVIHVPYR
	FSGKDVVIVATR
336	

Spot	Sequence
[illegible]	DLYINNAIQMDVTGSR
	GLEPSEFEDSVAQAFFDLENGNQELK
	QILATIPGR
	SLFSADAISR
	QILATIPGRATGAYSHSQGIK
	KAPDAFYALR
	TGWGLEISDLK
**337**	AEKDGILESLAR
	GLRDAIAAGIMSR
	TGWGLEISDLKMQLENAR
	AFHEAFLAEYRD.-
	LLESTGIVVVPGSGFR
	SMGYGEDDLPLVSLQSVSK
	HLFYQDASDLR
**338**	ALKDTLNWAVHR
	NPPPPQGIEIIYNYGK
	ANEFGIVLGK
	IIQIPTVLR
	DIDAAWEAVR
	SNREFLYHILGEVIK
	TPDQVVAIAREMVAYAR
	YKGTYEIISPDDIGLTR
	QLEVTINGIGER
	AGNASLEEVVMAIK
**339**	ELLDGLYTGIDSR
	EFLYHILGEVIK
	ANEFGIVLGKLSGR
	AVDKIIQIPTVLR
	LIADIKANTPGIEK
	GTYEIISPDDIGLTR
	SFSGSGASMDIVVSSVR
	EYSMTSVTEGIDAIATTR
	VTDEDLEALLSDEIFQPK
	LDLAGRDLTDSLMK
	AVFPSIVGRPR
	SYELPDGQVITIGAERFR
**340**	DLTDSLMKILTER
	SYELPDGQVITIGAER
	LAYIALDYEQELETAK
	VAPEEHPVLLTEAPLNPK
	NIPLVAFIGR

Spot	Sequence
342	VFVDHPLFLER<br>EALQAEVGLPVDR<br>FAFSDYPELNLPER<br>GVDRVFVDHPLFLER<br>ALNKEALQAEVGLPVDR<br>SSFDFIDGYEKPVEGR<br>IYGPDAGTDYRDNQLR<br>TGGLGDVLGGLPPAMAANGHR<br>AGILEADRVLTVSPYYAEELISGIAR
343	EGVEVAQLVEK<br>VSFGENFSPAR<br>LRSPAAEALGPTHLLHTR<br>VKEGVEVAQLVEK<br>SPAAEALGPTHLLHTR
352	SIFEIPGAK<br>EVAIETASFSK<br>LQAGYLFPEIAR<br>NAPYVWVHFPGR<br>NSWDVFAEILEK<br>ELLFSDGHPVAKDFNR<br>ANVVTTPGTGFGPGGEGFVR
354	VFLAGADNVLQKLDR<br>VLRPFDEVSR<br>NPESFLSSFSK<br>VFLAGADNVLQK
356	IAGLEVLR<br>TTPSVVAYTKTGER<br>IPAVQELVR<br>TTPSVVAYTK<br>QAVVNPENTFFSVKR<br>NQADSVVYQTEKQLK<br>IVDWLASNFK<br>IINEPTAASLAYGFEKK<br>MAEVDDEAKQVSYGVVK<br>DAGRIAGLEVLR<br>QDITITGASTLPKDEVER<br>IVDWLASNFKK<br>FDIDANGILSVAAIDKGTGK<br>LVDDASKFLNEK<br>AVVTVPAYFNDSQR

Spot	Sequence
	QFAAEEISAQVLRK
	QAVVNPENTFFSVK
	LSLSDLDEVILVGGSTR
	TFPVILLPK
	VKTFPVILLPK
	TFPVILLPKEK
**357**	GWETENIEEIALK.I
	SNVEYIAGGATQNSIR
	IAVITQGADPVVVAEDGK
	VRGWETENIEEIALK
	NIQEYKGPR
	HDPPIVLAK
	FIDASTIPR
	DFDVAALMK
	AAAAEPVKDEL-
	EADGIVDYLK
	TADDIVDFIK
	GDAAVERPLVR
	YEIQGFPTIK
	KLAPEYENAAK
	EADGIVDYLKK
	SDYDFGHTLHANHLPR
	TADDIVDFIKK
**358**	VVTFDKNPDNHPYLMK
	NRPLATKYEIQGFPTIK
	ALSKHDPPIVLAK
	SPEDATALIDDKK
	SAYSAAAEEFKDK
	SEPIPEVNNEPVK
	SEPIPEVNNEPVKVVVADNVHDFVFK
	VVVADNVHDFVFK
	LLKPFDELVVDSK
	KSEPIPEVNNEPVK
	EIKSPEDATALIDDK
	FLIGDIEASQGAFQYFGLKEDQTPLILIQDGDSK
	MDATANDVPSEFDVQGYPTLYFVTPSGKVTSYDSGR
	ALGDYLGVK
	GVAINFVTR
	DIIMREFR

Spot	Sequence
	VLITTDLLAR
	GFKDQIYDIFQLLPSK
	ELAQQIEKVMR
360	GLDVIQQAQSGTGK
	ILASGVHVVVGTPGR
	DQIYDIFQLLPSK
	MFVLDEADEMLSR
	GIYAYGFEKPSAIQQR
	MQELLSTGETEEFFTSYDEVFESFDDMGLQENLLR
	LESALAATEIR
362	LAVAGAFHTSFMQPAVSR
	IPVISNVDAQPHSDPNTIK
	EGLLQLPSDK
	HQSELAHGANAGLDIAVR
	QMGLSDQDIVALSGGHTLGR
	AFFDDYKEAHLK
363	LAWHSAGTFDVSSR
	ALLSDPVFRPLVEK
	NYPTVSAEYSEAVEK
	ELLSGDKEGLLQLPSDK
	NYPTVSAEYSEAVEKAR
	FRAPVEPY-<br>RIPLYQHIANLAGNK
	QLVLPVPAFNVINGGSHAGNK
	LGANAILAVSLAVCK
368	IEEELGAIAVYAGAK
	IPLYQHIANLAGNK
	AVNNVNSVIGPALIGK
	VNQIGSVTESIEAVK
	AAVPSGASTGVYEALELR
	LAMQEFMILPTGAASFK
	IAGLDVER
	HLNITLTR
	EVDEVLLVGGMTRVPK
	VQEVVSEIFGK
	EVDEVLLVGGMTR
	QYSPSQVGAFVLTK
	TTPSVVAFTQKGER

**Spot**	**Sequence**
**376**	QAVTNPQNTFFGTK
	VPKVQEVVSEIFGK
	AVITVPAYFNDAQR
	SKFESLVHDLIER
	LLGEFDLVGIPPAPR
	VQEVVSEIFGKNPSK
	QAVTNPQNTFFGTKR
	SQVFSTAADNQTQVGIR
	EGILSIWK
	TALAFVTLK
	FGDDEFGHMLVNILK
	LPLWPSPDAAR
	VSDDEVAFLTR
	KGAIPALPTVATAQDLIAK
**380**	NVLSLWFDGLK
	APGGAPANVACAIAK
	DDSIFHNEEKLR
	TALAFVTLKHDGER
	EGILSIWKEADFIK
	GAIPALPTVATAQDLIAK
	GAIPALPTVATAQDLIAKAN –
	AKIVHVDIDPAEIGK
	LPKPPATELLEQVLR
	SPVIANHLFR
	KGADILVESLER
	DVFAYPGGASMEIHQALTR
**381**	IVHVDIDPAEIGK
	HEQGEAFAASGYAR
	HNYLVLDVDDIPR
	LALQGMNALLEGSTSK
	IVHVDIDPAEIGKNK
	LALQGMNALLEGSTSKK
**382**	TPIDVAKLNNQDEVLK
	HGAAVTLQNLDGKTPIDVAK
	FDPDFIDIR
	LYDDLTTGFR
	LAADTPLLTGQR
	FTMLQTWPVR
	KLYDDLTTGFR
**385**	EDDLNEIVQLVGK

Spot	Sequence
	DALAESDKITLETAK<br>EASIYTGITIAEYFR<br>LAEMPADSGYPAYLAAR<br>VGHDNLIGEIIRLEGDSATIQVYEETAGLMVNDPVLR
387	LAWHSAGTFDVSSR<br>EGLLQLPSDK<br>HQSELAHGANAGLDIAVR<br>AKNYPTVSAEYSEAVEK<br>QMGLSDQDIVALSGGHTLGR<br>AFFDDYKEAHLK<br>ALLSDPVFRPLVEKYAADEK<br>ALLSDPVFRPLVEK<br>ELLSGDKEGLLQLPSDK<br>NYPTVSAEYSEAVEKAR
388	NLDEFVQFVR<br>QAAAASASLASLVEAR<br>GLLAAGKPFLWVVR
389	
392	LLDKLVVLK<br>SIPSIVELDSLK<br>VLQLETAAGAAIR<br>IVTEDFLPLPSK<br>FFDKAIGINVPR<br>VSGDVWFGSGITLK.G<br>IFNTNNLWVNLK<br>YLSGEAEQIEWSK<br>IVTEDFLPLPSKGK<br>FKSIPSIVELDSLK<br>LVEAEALKMEIIPNPK
394	TIAWLDKLR<br>AFGGQSLDFGKGGQAYR<br>LGANSLLDIVVFGR<br>AVIELENYGLPFSR<br>LAYRPVHMNTLDDEVESFPPK
395	ADLVVDVLIK<br>IPVSLVFDDIKSTYK<br>IDVDTPFGNMKLPISK<br>IPVSLVFDDIK<br>VVLLVDVPIIGR

Spot	Sequence
	DFGSAVWDMIR
	DIQPGSIIPYLVR
	DGEIPIPYKPDVDVEK
	LYKEHIPVTQLVR
	AANGVVIATEK
	LVQIEHALTAVGSGQTSLGIK
**398**	LPSILVDETSVQK
	IQALTPNIGVVYSGMGPDFR
	KLPSILVDETSVQK
	VLSPAEIKDFLEEVE.-
	LLNDEFYIGLR
**399**	ILGLGDLGSQGMGIPVGK
	AIFASGSPFDPVEYDGK
	LPDATQGSDHLR
	LAWHSAGTFDVATK
	ALLSDPSFRPLVDK
**403**	EGLLQLPSDK
	AYPTVNEDYLK
	TGGPFGTMKNPAEQAHGANAGLEIAIR
	VKAYPTVNEDYLK

Spot	Modification	Peptide score*	Protein	M_r (kDa) / pI Theor
1	-----	46	Late embryogenesis abundant protein, group 3	18588/ 7.85
	-----	**64**		
	-----	48		
4	Oxidation (M)	28	22.0 kDa class IV heat shock protein precursor	22872/ 6.01
	------	**96**		
	Glu->pyro-Glu (N-term E); Oxidation (M)	18		
	-----	9		
	-----	31		
	-----	22		
5	------	21	Fasciclin-like arabinogalactan protein 8 precursor	44699/ 6.56
	------	45		
	Gln->pyro-Glu (N-term Q)	**58**		
6			ND	
9	------	24	Unknown	19898/11.53
	------	**52**		
11			ND	
12	------	34	Unknown	17757/ 6.62
	------	**54**		
	-------	**54**		
	------	17		
	------	45		
	------	31		
13	------	**52**	Heat shock protein 17.2	17152/ 5.54
	------	43		
	------	**74**		
15	-------	**54**	Vicilin-like embryo storage protein	66122/ 6.23
	-------	**57**		
	-------	39		
17	-------	**81**	rab28	27693/ 4.90
	-------	**57**		
	-------	**73**		
	------	**96**		
	------	**82**		
	-------	**103**		

Spot	Modification	Peptide score*	Protein	M_r (kDa) / pI Theor
	-------	**87**		
	Deamidated (NQ)	**67**		
21	------	**57**	Zein-alpha 19D1 precursor	26616/ 9.21
	Deamidated (NQ)	9		
	------	**58**		
	-------	22		
	-------	24		
24	-------	**43**	Putative aconitate hydratase	98021/ 5.67
	-------	19		
	-------	38		
	------	**59**		
26	-------	48	Vicilin-like embryo storage protein	66122/ 6.23
	-------	47		
	-------	**50**	Actin depolymerizing factor	
29	-------	**64**		15890/ 5.46
	Deamidated (NQ)	**66**		
	-------	49		
	-------	27	Prohibitin3	
	Oxidation (M)	**62**		
	-------	**51**		
36	Oxidation (M)	30		30580/ 7.00
	-------	32		
	-------	37		
	-------	16		
	Oxidation (M)	43		
	2 Oxidation (M)	38		
	------	**68**		
	------	47		
	Gln->pyro-Glu (N-term Q)	**63**		
	Oxidation (M)	**58**		
	Oxidation (M)	15		
	------	28		
	Oxidation (M)	44		
	Oxidation (M)	44		
	Deamidated (NQ)	17		
	------	**85**		
	------	48		
38	------	**81**	Pyruvate orthophosphate dikinase	102444/ 5.71
	------	46		

Spot	Modification	Peptide score*	Protein	M_r (kDa) / pI Theor
	------	38		
	------	**90**		
	2 Oxidation (M)	4		
	Oxidation (M)	29		
	-------	42		
	Oxidation (M)	27		
	-------	**51**		
	-------	**91**		
	-------	**101**		
	-------	**98**		
	-------	49		
	Oxidation (M)	31		
	+ 2 Oxidation (M)	15		
**40**			ND	
**43**	------	**79**	C4-specific pyruvate orthophosphate dikinase	102343/ 5.50
	------	**83**		
	------	**62**		
	Carbamidomethyl (C)	**57**		
	Oxidation (M)	36		
	-------	**60**		
	------	**68**		
	------	31		
	------	**77**		
	------	**63**		
	2 Oxidation (M)	2		
	------	48		
	3 Oxidation (M)	**79**		
	------	**72**		
	------	**54**		
	Deamidated (NQ)	**103**		
	Deamidated (NQ); Oxidation (M)	28		
**44**	------	**77**	Vicilin-like embryo storage protein	66122/ 6.23
	------	**63**		
	------	**51**		
	------	**55**		
	------	18		
	------	27		
	------	21		
	------	30		

Spot	Modification	Peptide score*	Protein	M_r (kDa) / pI Theor
	------	27		
45	------	**66**	Prohibitin 2	30702/ 6.55
	------	42		
	------	24		
	------	**61**		
49	------	33	Activator of 90 kDa heat shock protein ATPase	38577/5.33
	------	38		
	------	**58**		
	------	**53**		
50	------	**70**	Unknown	29193/ 8.44
	------	28		
	------	**65**		
	------	**53**		
	Carbamidomethyl (C)	33		
	------	**60**		
	2 Deamidated (NQ)	29		
	Oxidation (M)	**51**		
	------	**63**		
	------	**54**		
	Deamidated (NQ)	**92**		
51	-------	**62**	Vicilin-like embryo storage protein	66122/ 6.23
	-------	**63**		
	-------	44		
	Oxidation (M)	38		
	Oxidation (M)	50		
	2 Oxidation (M)	43		
	------	**87**		
	------	**66**		
	Gln->pyro-Glu (N-term Q)	46		
	Oxidation (M)	24		
	------	**65**		
	------	**55**		
	------	45		
	------	46		
	-------	42		
	-------	48		
	--------	**75**		

Spot	Modification	Peptide score*	Protein	M_r (kDa) / pI Theor
	Gln->pyro-Glu (N-term Q)	47		
	------	**101**		
**52**	------	**54**	Chain A, Pyruvate Phosphate Dikinase	95132/ 5.27
	------	**52**		
	2 Oxidation (M)	13		
	------	**97**		
	2 Oxidation (M)	8		
	Oxidation (M)	**75**		
	------	45		
	Oxidation (M)	25		
	Oxidation (M)	32		
	------	49		
	Oxidation (M)	**76**		
	-------	**100**		
	3 Oxidation (M)	**82**		
	------	**94**		
	Oxidation (M)	23		
	3 Oxidation (M)	34		
	-------	**72**		
	Oxidation (M)	7		
	Oxidation (M)	36		
	Oxidation (M)	33		
	Oxidation (M)	9		
	--------	**73**		
	Gln->pyro-Glu (N-term Q)	41		
	-------	24		
**53**	-------	**69**	Pyruvate orthophosphate dikinase	102444/ 5.71
	Deamidated (NQ)	39		
	+ 3 Oxidation (M)	44		
	-------	**73**		
	Deamidated (NQ); Oxidation (M)	9		
	Oxidation (M)	45		
	-------	**59**		
	-------	51		
	-------	26		
	-------	32		
	-------	46		
**54**	Oxidation (M)	**59**	Phosphoglucomutase 1	63058/ 5.46
	-------	23		

Spot	Modification	Peptide score*	Protein	M_r (kDa) / pI Theor
	-------	37		
	2 Oxidation (M)	29		
	Oxidation (M)	30		
	-------	15		
	------	42		
	------	51		
	------	48		
	------	**80**		
	------	**55**		
**55**	------	33	Heat shock 70 kDa protein	72704/ 5.62
	Gln->pyro-Glu (N-term Q)	**77**		
	-----	**77**		
	Oxidation (M)	22		
	------	**54**		
	------	15		
	------	**52**		
	------	14		
	Deamidated (NQ); Oxidation (M)	48		
	Oxidation (M)	**55**		
	------	**62**		
**59**	------	**95**	Unknown	38371/ 6.30
	------	**65**		
	------	**75**		
	------	**69**		
	------	51		
	------	**58**		
	------	**56**		
	------	**62**		
	------	32		
	------	**68**		
	------	28		
	------	**54**		
	Carbamidomethyl (C)	**56**		
	------	**74**		
	------	**76**		
**61**	------	**73**	Late embryogenesis abundant protein Lea14-A	16078/ 8.05
	------	27		
	Oxidation (M)	16		

Spot	Modification	Peptide score*	Protein	M_r (kDa) / pI Theor
	Carbamidomethyl (C)	**48**		
	------	25		
	------	**64**		
	-------	21		
	------	**62**		
**64**	-------	**115**	z1B alpha zein protein	16047/ 8.00
	-------	**84**		
	-------	**54**		
**66**	-------	52	Unknown	25739/ 8.49
	-------	**98**		
	-------	52		
	-------	36		
	-------	35		
	-------	**54**		
	-------	22		
	-------	46		
	-------	**57**		
	-------	**60**		
	-------	32		
**67**	-------	51	Phosphoglucomutase 2	63002/ 5.47
	-------	46		
	-------	**59**		
	2 Oxidation (M)	**79**		
	Oxidation (M)	**68**		
	-------	48		
	-------	43		
	2 Oxidation (M)	27		
	Oxidation (M)	42		
	-------	27	Unknown	
	-------	43		
	-------	**67**		
	-------	40		
	-------	**57**		
	-------	**58**		
	-------	**55**		
	-------	**80**		
**69**	Carbamidomethyl (C)	20		32924/ 5.78
	Deamidated (NQ)	38		

Spot	Modification	Peptide score*	Protein	M_r (kDa) / pI Theor
	Deamidated (NQ)	**52**		
	-------	**67**		
	-------	46		
	Carbamidomethyl (C)	27		
	Deamidated (NQ)	**75**		
	Deamidated (NQ); Oxidation (M)	19		
74	-------	**56**	Pyruvate orthophosphate dikinase	102471/ 5.52
	Oxidation (M)	**54**		
	Oxidation (M)	47		
	-------	**53**		
	Gln->pyro-Glu (N-term Q); 2 Oxidation (M)	**66**		
	Oxidation (M)	33		
	2 Deamidated (NQ)	48		
	-------	**77**		
	-------	**66**		
	-------	**79**		
	-------	43		
	2 Deamidated (NQ)	49		
	2 Deamidated (NQ); Oxidation (M)	**66**		
78	-------	35	Unknown	20867/ 6.84
	-------	36		
	-------	**58**		
	-------	38		
	-------	**97**		
	-------	23		
	-------	**62**		
	-------	17		
83	-------	32	Unknown	38371/ 6.30
	Oxidation (M)	**53**		
	-------	48		
	-------	30		
	-------	47		
	-------	49		
	-------	9		
	-------	18		
	-------	**57**		

Spot	Modification	Peptide score*	Protein	M_r (kDa) / pI Theor
	-------	**59**		
86	Oxidation (M)	32	OSJNBa0039C07.4	98436/ 5.79
	------	11		
	-------	31		
	-------	39		
	Gln->pyro-Glu (N-term Q)	46		
	Oxidation (M)	24		
	-------	44		
	-------	**56**		
	-------	45		
	-------	26		
	-------	**81**		
	Oxidation (M)	44		
	Oxidation (M)	42		
87	Oxidation (M)	**54**	Putative heat shock protein	82531/ 5.43
	-------	22		
96	-------	**53**	Pyruvate orthophosphate dikinase	102471/ 5.52
	Oxidation (M)	**55**		
	Oxidation (M)	21		
	------	46		
	Gln->pyro-Glu (N-term Q); 2 Oxidation (M)	**59**		
	Oxidation (M)	37		
	2 Deamidated (NQ)	39		
	------	**71**		
	------	49		
	------	**54**		
	-------	37		
	2 Deamidated (NQ)	43		
	2 Oxidation (M)	6		
104	-------	46	Unknown	34010/ 4.82
	------	**90**		
	-------	**57**		
	-------	15		
	Oxidation (M)	51		
	-------	20		
	-------	**78**		

Spot	Modification	Peptide score*	Protein	M_r (kDa) / pI Theor
	Gln->pyro-Glu (N-term Q); 2 Oxidation (M)	**55**		
	Oxidation (M)	36		
	Oxidation (M)	44		
	2 Deamidated (NQ)	45		
**107**	Gln->pyro-Glu (N-term Q)	47	Pyruvate orthophosphate dikinase	102471/ 5.52
	-------	**87**		
	-------	**73**		
	-------	**58**		
	Oxidation (M)	34		
	-------	48		
	Deamidated (NQ); Oxidation (M)	49		
	2 Deamidated (NQ)	**57**		
	Deamidated (NQ); Oxidation (M)	**106**		
	-------	27		
	Oxidation (M)	21		
	Oxidation (M)	18		
	------	46		
	------	**85**		
	Oxidation (M)	**54**		
**108**	------	**58**	Os02g0519900	93961/ 5.85
	------	**72**		
	Oxidation (M)	44		
	Oxidation (M)	**64**		
	------	15		
	------	39		
	------	**56**		
	------	**64**		
	------	**46**		
**111**	------	25	Nucleoside diphosphate kinase 1	16835/6.3
	------	43		
	------	24		
	------	**59**		
	------	46		
	------	**51**		
	-------	**59**		
**112**	Oxidation (M)	**71**	Unknown	38566/ 7.52
	-------	50		

Spot	Modification	Peptide score*	Protein	M_r (kDa) / pI Theor
	-------	**69**		
	-------	**83**		
	-------	**81**		
	Oxidation (M)	11		
	-------	**74**		
	-------	30		
	-------	41		
	-------	27		
**114**	-------	50	Unknown	19336/ 5.14
	-------	50		
	Oxidation (M)	25		
	-------	**57**		
	Oxidation (M)	30		
	------	36	Putative aconitate hydratase 1	
	------	16		
	Oxidation (M)	38		
	Deamidated (NQ); Oxidation (M)	26		
	Deamidated (NQ)	35		
	Oxidation (M)	**55**		
**134**	Deamidated (NQ)	**70**		106913/ 6.63
	------	41		
	------	16		
	Oxidation (M)	38		
	Oxidation (M)	35		
	Oxidation (M)	50		
	------	11		
	Deamidated (NQ)	15		
	------	37		
	------	**59**		
	------	40		
	------	34		
	------	41		
	Oxidation (M)	45		
**136**	------	40	Phosphoglycerate mutase	60592/ 5.29
	Oxidation (M)	44		
	------	24		
	------	50		
	------	**74**		
	Oxidation (M)	24		
	------	42		

Spot	Modification	Peptide score*	Protein	M_r (kDa) / pI Theor
	Oxidation (M)	13		
	Oxidation (M)	30		
139	-------	43	Translation initiation factor 5A	17486/ 5.61
	-------	**54**		
145	Oxidation (M)	48	3-phosphoglycerate kinase	42413/ 5.65
	------	**56**		
	------	27		
	Oxidation (M)	46		
	------	35		
	------	31		
	------	**95**		
	------	**67**		
	------	**55**		
	------	**95**		
	------	**97**		
	------	51		
	Oxidation (M)	32		
	------	**72**		
	------	**67**		
	------	**77**		
	------	**68**		
	Oxidation (M)	**59**		
	Deamidated (NQ); Oxidation (M)	**67**		
	Oxidation (M)	**56**		
147	------	26	Catalase isozyme 1	56841/ 7.40
	------	31		
	Oxidation (M)	**55**		
	------	31		
	Oxidation (M)	30		
148	------	39	Os12g0230100	101954/ 6.62
	------	22		
	------	45		
	------	20		
	------	20		
	------	**60**		
	Oxidation (M)	37		
	------	46		
	------	43		
	------	50		

Spot	Modification	Peptide score*	Protein	M_r (kDa) / pI Theor
	2 Oxidation (M)	23		
	------	42		
	------	53		
	2 Oxidation (M)	32		
**149**	------	21	Heat-shock protein 101	101069/ 5.85
	------	26		
	------	28		
	------	**77**		
	2 Oxidation (M)	19		
	2 Deamidated (NQ)	11		
	------	**53**		
	------	36		
	-------	44		
	-------	30		
	------	20		
	-------	16		
	-------	**74**		
**154**	Oxidation (M)	21	Unknown	80628/ 6.10
	-------	16		
	Oxidation (M)	22		
	Oxidation (M)	**67**		
	-------	28		
	Deamidated (NQ)	34		
	-------	41		
	-------	50		
	Oxidation (M)	**68**		
	Oxidation (M)	25		
	------	27		
	------	**82**	Glyceraldehyde-3-phosphate	
**163**	Oxidation (M)	**60**	dehydrogenase	24930/ 8.44
	------	26		
	Oxidation (M)	**104**		
	------	47		
	------	**57**		
	------	**67**		
	------	**81**		
	------	45		
	------	20		
	Oxidation (M)	**65**		
	------	**57**		
**164**	------	48	Phosphoglucomutase 2	63002/ 5.47

Spot	Modification	Peptide score*	Protein	M_r (kDa) / pI Theor
	------	16		
	Oxidation (M)	39		
	------	21		
	------	9		
	------	34		
	------	**56**		
**170**	-------	72	Actin depolymerizing factor	15890/ 5.46
	Deamidated (NQ)	**82**		
	------	**78**		
	------	**60**		
	------	**82**		
	------	47		
	------	**53**		
**172**	------	48	Lactoylglutathione lyase	35140/ 6.62
	------	15		
	------	52		
	Oxidation (M)	40		
	------	44		
	------	51		
	------	**82**		
	------	32		
	2 Oxidation (M)	13		
	------	32		
**174**	------	**60**	Unknown	64846/ 5.51
	------	**78**		
	Oxidation (M)	24		
	------	**74**		
	------	49		
	------	**69**		
	------	25		
**175**			ND	
	Oxidation (M)	21		
	--------	46		
	-------	**106**		
	--------	52		
	--------	**79**		
**178**	Oxidation (M)	54	Os02g0519900	93961/ 5.85
	Oxidation (M)	9		
	-------	22		
	-------	**56**		
	------	**60**		

Spot	Modification	Peptide score*	Protein	M_r (kDa) / pI Theor
	------	45		
	Oxidation (M)	**95**		
	Oxidation (M)	**76**		
	Oxidation (M)	**67**		
	Deamidated(NQ)	40		
	------	18		
	Deamidated (NQ); Oxidation (M)	34		
**179**	------	**67**	Legumin1	52798/ 6.20
	Oxidation (M)	43		
	Oxidation (M)	28		
	Oxidation (M)	48		
	Deamidated (NQ); Oxidation (M)	27		
**185**	Oxidation (M)	**42**	Putative aconitate hydratase	98021/ 5.67
	-------	33		
	-------	20		
**186**	Oxidation (M)	**42**	Putative aconitate hydratase	98021/ 5.67
	-------	26		
	-------	**52**		
	-------	21		
**188**	-------	32	Vicilin-like embryo storage protein	66122/ 6.23
	Gln->pyro-Glu (N-term Q)	29		
	Oxidation (M)	43		
**189**	Oxidation (M)	50	Unknown	19915/ 5.19
	------	**55**		
	------	**59**		
	-------	29		
	------	14		
	------	28		
	-------	21		
**192**	-------	14	Methionine synthase protein	83736/ 5.93
	-------	**55**		
	Oxidation (M)	**65**		
	-------	52		
	-------	52		
	Deamidated (NQ)	43		
	Deamidated (NQ)	15		
	-------	49		
	-------	46		
	-------	34		

Spot	Modification	Peptide score*	Protein	M_r (kDa) / pI Theor
196	-------	**85**	Glyceraldehyde-3-phosphate dehydrogenase	36428/ 6.61
	-------	47		
	Deamidated (NQ); Oxidation (M)	29		
	-------	**66**		
	-------	35		
	Carbamidomethyl (C)	45		
	-------	42		
	-------	32		
	-------	**92**		
	-------	10		
	Deamidated (NQ); Oxidation (M)	18		
	Deamidated (NQ); Oxidation (M)	21		
	-------	46		
197	-------	**61**	Unknown	37857/ 6.70
	-------	20		
	Oxidation (M)	**62**		
	-------	54		
	Oxidation (M)	**72**		
	-------	49		
	-------	**113**		
	-------	**75**		
200			ND	
203			ND	
205	Deamidated (NQ)	48	Glyceraldehyde-3-phosphate dehydrogenase	36519/ 6.41
	Deamidated (NQ)	34		
	Deamidated (NQ)	39		
	-------	49		
	-------	46		
	------	**83**		
	Oxidation (M)	**60**		
	------	18		
	------	**63**		
	Deamidated (NQ); Oxidation (M)	**57**		
	------	49		
	------	**65**		
	-------	20		
	Oxidation (M)	**53**		

Spot	Modification	Peptide score*	Protein	M_r (kDa) / pI Theor
	-------	**63**		
	Oxidation (M)	43		
	Oxidation (M)	**76**		
**206**	------	27	Chain A, Bifunctional Hageman Factor AMYLASE INHIBITOR FROM MAIZE	13570/ 6.51
	Carbamidomethyl (C)	31		
	Carbamidomethyl (C)	**49**		
	2 Carbamidomethyl (C)	16		
	Carbamidomethyl (C); Oxidation (M)	27		
**207**	-------	26	QM protein	24903/ 10.27
	-------	**67**		
**210**	------	42	Granule-bound starch synthase precursor	66567/ 6.59
	-------	23		
	------	45		
	Deamidated (NQ)	39		
	------	18		
	------	44		
	------	22		
	Carbamidomethyl (C)	**85**		
	------	35		
	------	**58**		
	------	49		
**213**	------	**92**	Hypothetical protein Z477F24.14	15597/ 4.94
	------	52		
	------	**62**		
	-------	**59**		
**214**	------	**42**	Glyoxalase I	32336/ 5.59
	------	23		
	-----	23		
	Oxidation (M)	20		
**219**	-------	**81**	ATP synthase beta chain	45679/ 4.92
	Gln->pyro-Glu (N-term Q)	40		
	Oxidation (M)	**63**		
	------	**66**		
	------	19		
	------	**75**		
	Oxidation (M)	44		

Spot	Modification	Peptide score*	Protein	M_r (kDa) / pI Theor
	-------	**66**		
	-------	34		
	-------	46		
**242**	-------	12	Vicilin-like embryo storage protein	66122/ 6.23
	-------	**64**		
	-------	40		
	Gln->pyro-Glu (N-term Q)	52		
	-------	**49**		
**245**	------	17	Phosphoglucomutase 2	63002/ 5.47
	------	11		
	-------	30		
**249**	------	27	Phosphoglycerate mutase	60592/ 5.29
	------	**51**		
	-------	18		
	-------	46		
	-------	44		
	-------	**56**		
	-------	30		
**251**	--------	**63**	Glyceraldehyde-3-phosphate dehydrogenase	36428/ 6.61
	-------	54		
	-------	26		
	------	18		
	Deamidated (NQ)	9		
	-------	33		
	-------	36		
	-------	28		
	-------	**57**		
	-------	50		
	Oxidation (M)	**71**		
**252**	Oxidation (M)	**89**	Actin	41699/ 5.24
	Oxidation (M)	50		
	------	**93**		
	-------	**56**		
	-------	**71**		
	-------	55		
**257**			ND	
**261**	5 Oxidation (M)	**80**	Zein protein precursor	19448/ 8.05
**262**			ND	

Spot	Modification	Peptide score*	Protein	M_r (kDa) / pI Theor
	------	**61**		
	-------	**77**		
	------	47		
	------	50		
	-------	36		
**265**	------	43	Os02g0102900	63759/ 5.77
	Oxidation (M)	**63**		
	------	54		
	------	39		
	Deamidated (NQ); Oxidation (M)	26		
**266**	Oxidation (M)	**35**	Glycine-rich RNA binding protein	15908/ 5.22
	Oxidation (M)	29		
**267**			ND	
	Oxidation (M)	**59**		
	------	47		
**268**	------	**60**	Malate dehydrogenase5	35567/ 5.77
	------	**59**		
	Deamidated (NQ)	23		
	------	**79**		
	------	28	Hypothetical protein LOC100191552	
	------	45		
**272**	------	23		17869/ 6.86
	Deamidated (NQ)	**71**		
	------	**53**		
	------	**58**		
	--------	18		
	Oxidation (M)	16		
	--------	48		
**273**	Oxidation (M)	35	Unknown	25571/ 7.11
	--------	25		
	--------	35		
	--------	**51**		
	--------	34		
	--------	36		
	Oxidation (M)	30		
	--------	20		
	-------	38		
**274**	------	53	Sorbitol dehydrogenase	39063/ 6.27
	-------	**108**		
	Oxidation (M)	**57**		

Spot	Modification	Peptide score*	Protein	M_r (kDa) / pI Theor
	-------	**91**		
	-------	32		
	Oxidation (M)	27		
	------	19		
	Oxidation (M)	37		
	-------	**58**		
**276**	Oxidation (M)	16	Pyruvate, orthophosphate dikinase	102444/ 5.71
	2 Oxidation (M)	53		
	3 Oxidation (M)	35		
	-------	24		
	Deamidated (NQ)	35		
	-------	**56**		
	-------	**91**		
**278**	-------	**65**	Enolase1	48033/ 5.2
	-------	53		
	-------	16		
	-------	**83**		
	-------	14		
	-------	17		
**282**	-------	**43**	Unknown	40439/ 5.72
	-------	36		
	------	**58**		
	------	**52**		
	------	**72**		
	Deamidated (NQ)	**57**		
	------	**65**		
	Oxidation (M)	24		
	------	43		
**284**	------	50	Unknown	59158/ 5.78
	Oxidation (M)	**79**		
	Oxidation (M)	**75**		
	Deamidated (NQ)	**67**		
	Deamidated (NQ)	**83**		
	Oxidation (M)	33		
	-------	34		
	------	47		
	------	**55**		
	-------	**40**		
	-------	32		
**286**	-------	28	Unknown	25961/ 4.76
	-------	31		

Spot	Modification	Peptide score*	Protein	M_r (kDa) / pI Theor
	-------	16		
	-------	**80**		
	-------	20		
	-------	32		
	-------	**63**		
	-------	**62**		
**287**	-------	**68**	Os06g0114000	64046/ 5.60
	Deamidated (NQ)	38		
	-------	**71**		
	-------	49		
	-------	51		
	-------	51		
	Oxidation (M)	27		
	------	37		
	------	**63**		
	------	**56**		
	------	35		
	------	26		
**292**	------	22	Unknown	62963/ 6.31
	------	17		
	Oxidation (M)	21		
	Oxidation (M)	7		
	Oxidation (M)	37		
	------	26		
	Gln->pyro-Glu (N-term Q)	51		
	Deamidated (NQ); Oxidation (M)	14		
	Deamidated (NQ)	27		
	------	54		
	Oxidation (M)	**56**		
	Oxidation (M)	**63**		
	------	**56**		
	------	**87**	Glyceraldehyde-3-phosphate	
**305**	Oxidation (M)	**72**	dehydrogenase	24930/ 8.44
	------	11		
	Oxidation (M)	16		
	------	52		
	Deamidated (NQ)	25		
	------	28		
	------	**89**		

Spot	Modification	Peptide score*	Protein	M_r (kDa) / pI Theor
	------	**76**		
309	------	46	Unknown	60980/ 5.96
	2 Oxidation (M)	45		
	------	**63**		
	Deamidated (NQ)	16		
311	------	**64**	Unknown	42413/ 5.65
	------	44		
	------	**53**		
	------	24		
	Oxidation (M)	**79**		
	------	**74**		
	------	35		
	------	48		
	------	33		
	------	**79**		
	-------	**95**		
	Oxidation (M)	50		
	-------	**66**		
	------	**77**		
	------	**77**		
	Oxidation (M)	21		
	------	**105**		
312	------	13	Unknown	37857/ 6.7
	------	30		
	Oxidation (M)	39		
	------	**54**		
	------	**53**		
	Oxidation (M)	**62**		
	------	46		
	Oxidation (M)	33		
	-------	27		
	-------	32		
	------	**72**		
318	-------	39	Peptidyl-prolyl cis-trans isomerase	18337/ 8.91
	Oxidation (M)	**81**		
	-------	41		
	-------	49		
	Oxidation (M)	16		
	Oxidation (M)	**64**		
	Oxidation (M)	45		
	Oxidation (M)	33		

Spot	Modification	Peptide score*	Protein	M_r (kDa) / pI Theor
	Oxidation (M)	**86**		
319	------	48	Nucleoside diphosphate kinase 1	16835/ 6.30
	------	**55**		
	------	41		
	------	29		
321	------	31	Enolase1	48033/ 5.20
	------	**59**		
	------	34		
	------	50		
	Oxidation (M)	43		
	Deamidated (NQ)	30		
	------	**98**		
	Deamidated (NQ)	**55**		
	------	**80**		
	------	**70**		
	------	**81**		
	------	**99**		
	------	**62**		
	------	40		
	------	**87**		
	------	**61**		
	------	27		
326	------	27	Enolase1	48033/ 5.20
	------	**39**		
328	Deamidated (NQ)	26	Unknown	67138/ 7.02
	------	**69**		
	------	38		
	------	38		
329	------	39	Unknown	22630/ 4.89
	------	**68**		
334	------	**58**	Carbonyl reductase 1	32662/ 6.16
	------	**73**		
	------	**88**		
	------	**93**		
	------	**64**		
	------	49		
335	------	**58**	Unknown	17312/ 4.85
	------	**59**		
	Oxidation (M)	**56**		
336	------	**52**	Unknown	22198/ 9.76
	------	**59**		

Spot	Modification	Peptide score*	Protein	M_r (kDa) / pI Theor
[illegible]	Oxidation (M)	41		[illegible]
	Deamidated (NQ)	36		
	------	**53**		
	------	**59**		
	------	19		
	------	**63**		
	------	**85**		
**337**	------	34	Alanine aminotransferase 2	53000/ 6.23
	Oxidation (M)	**56**		
	Oxidation (M)	**57**		
	------	41		
	------	**59**		
	Oxidation (M)	**62**		
	------	49		
**338**	------	**95**	Unknown	13346/ 8.01
	------	14		
	------	**72**	Unknown	
	------	38		
	------	49		
	------	30		
	Oxidation (M)	42		
	------	32		
	------	**60**		
	Oxidation (M)	**65**		
**339**	------	**77**		67138/ 7.02
	------	23		
	------	**85**		
	------	41		
	------	51		
	------	**54**		
	------	**61**		
	Oxidation (M)	**80**		
		**64**		
	Oxidation (M)	50		
	------	25		
	------	26		
**340**	Oxidation (M)	30	Hypothetical protein LOC100191561	41699/ 5.24
	------	**61**		
	------	**54**		
	------	**54**		
	------	34		

Spot	Modification	Peptide score*	Protein	M_r (kDa) / pI Theor
342	------	40	Granule-bound starch synthase precursor	66567/ 6.59
	------	**87**		
	------	**55**		
	------	22		
	------	47		
	------	48		
	------	21		
	Deamidated (NQ); Oxidation (M)	29		
	------	**63**		
343	------	**55**	Unknown	26645/ 7.11
	------	**80**		
	------	**53**		
	------	**62**		
	------	**90**		
352	------	43	Unknown	49566/ 6.55
	------	38		
	------	44		
	------	**57**		
	------	**57**		
	------	48		
	------	**73**		
354	------	**54**	Vicilin-like embryo storage protein	66122/ 6.23
	------	27		
	------	**59**		
	------	46		
356	------	**58**	Unknown	74625/ 5.08
	------	43		
	------	46		
	------	43		
	------	**55**		
	------	23		
	------	49		
	------	**90**		
	Oxidation (M)	36		
	------	32		
	------	**56**		
	------	**55**		
	Deamidated (NQ)	**68**		
	------	**70**		
	------	**72**		

Spot	Modification	Peptide score*	Protein	M_r (kDa) / pI Theor
	Gln->pyro-Glu (N-term Q)	**70**		
	------	**66**		
	------	**70**		
	------	25		
	------	20		
	------	50		
**357**	------	**75**	Adenosine kinase	36009/ 5.23
	------	**97**		
	------	40		
	------	**55**		
	------	29		
	------	43		
	------	**54**		
	Oxidation (M)	41		
	------	48		
	------	**64**		
	------	**61**		
	------	42		
	------	48		
	------	43		
	------	**59**		
	------	**79**		
	------	**71**		
**358**	Oxidation (M)	33	Protein disulfide isomerase	56838/ 5.01
	------	**58**		
	------	47		
	------	**95**		
	------	**69**		
	------	**62**		
	------	33		
	------	**53**		
	------	**51**		
	------	33		
	------	**75**		
	3 Deamidated (NQ)	20		
	2 Deamidated (NQ); Oxidation (M)	3		
	------	**55**		
	------	**53**		
	Oxidation (M)	16		

Spot	Modification	Peptide score*	Protein	M_r (kDa) / pI Theor
	------	20		
	------	**55**		
	Oxidation (M)	34		
**360**	------	**53**	Translational initiation factor eIF-4A	46952/ 5.37
	------	**70**		
	------	27		
	2 Oxidation (M)	29		
	------	**80**		
	Deamidated (NQ); 2 Oxidation (M)	19		
	------	**59**	Unknown	
**362**	Oxidation (M)	42		38593/ 5.64
	------	39		
	------	32	Unknown	
	------	**57**		
	Deamidated (NQ); Oxidation (M)	43		
	------	47		
**363**	------	**66**		27368/ 5.65
	------	**80**		
	------	49		
	------	43		
	------	42		
	------	23		
	------	33		
	------	19		
	Carbamidomethyl (C)	44		
**368**	------	**73**	Enolase2	48132/ 5.70
	------	48		
	------	**62**		
	------	**55**		
	------	**84**		
	Oxidation (M)	23		
	------	39		
	------	24		
	Oxidation (M)	33		
	------	44		
	Oxidation (M)	**57**		
	Gln->pyro-Glu (N-term Q)	**90**		
	------	46		

Spot	Modification	Peptide score*	Protein	M_r (kDa) / pI Theor
376	------	**65**	Unknown	72704/ 5.62
	------	31		
	Deamidated (NQ)	**85**		
	------	**73**		
	------	**73**		
	------	**85**		
	Gln->pyro-Glu (N-term Q)	**53**		
	2 Deamidated (NQ)	**126**		
	------	**55**		
	------	47		
	Oxidation (M)	42		
	------	31		
	------	**80**		
	------	**75**		
380	------	42	Fructokinase 2	35459/ 5.34
	------	36		
	------	18		
	------	**82**		
	------	**70**		
	Oxidation (M)	50		
	Deamidated (NQ)	12		
	------	**52**		
	------	**68**		
	------	**52**		
	------	**89**		
	Oxidation (M)	**61**		
381	------	**69**	Acetolactate synthase 1	68887/ 6.69
	------	23		
	------	**84**		
	------	24		
	------	26		
	Deamidated (NQ); Oxidation (M)	30		
382	------	32	Unknown	36227/ 4.50
	------	**69**		
	------	**53**		
	------	**57**		
	------	**71**		
	Oxidation (M)	49		
	------	**76**		
385	------	16	Unknown	68376/ 5.30

Spot	Modification	Peptide score*	Protein	M_r (kDa) / pI Theor
	------	23		
	------	**54**		
	Oxidation (M)	22		
	Deamidated (NQ); Oxidation (M)	11		
	------	42		
	------	36		
	------	**74**		
	------	**57**		
	Oxidation (M)	**82**		
**387**	------	**67**	Unknown	27368/ 5.65
	------	23		
	------	**60**		
	------	**65**		
	------	**53**		
	------	34	IAA-glu synthetase	
**388**	------	**108**		49679/ 5.75
	------	**63**		
**389**			ND	
	------	**57**		
	------	**64**		
	------	**60**		
	------	**67**		
	------	**61**		
**392**	------	**81**	Unknown	52056/ 5.30
	------	**69**		
	------	**59**		
	------	**78**		
	------	21		
	Oxidation (M)	**52**		
	-------	51	Succinate dehydrogenase flavoprotein subunit	
	-------	47		
**394**	-------	**87**		67941/ 6.08
	--------	**90**		
	Oxidation (M)	21		
	------	49	Hypothetical protein LOC100191638	
	------	41		
	Oxidation (M)	34		
**395**	------	50		35252/ 4.92
	------	**63**		

Spot	Modification	Peptide score*	Protein	M_r (kDa) / pI Theor
	Oxidation (M)	**53**		
	------	45		
	------	33		
	------	39	Hypothetical protein LOC100193683	
	------	21		
	------	**74**		
**398**	------	**55**		25848/ 5.53
	Oxidation (M)	**58**		
	------	37		
	------	22		
	------	49	Non-photosynthetic NADP-malic enzyme	
**399**	Oxidation (M)	36		70622/ 6.46
	------	19		
	------	43	Unknown	
	------	**64**		
	------	29		
**403**	------	**74**		27211/ 5.28
	------	48		
	Oxidation (M)	**65**		
	------	**58**		

Spot	AC number (gi NCBI) and reference organism
1	gil195605580 *Zea mays*
4	gil195644560 *Zea mays*
5	gil195607426 *Zea mays*
6	
9	gil194695412 *Zea mays*
11	
12	gil194695608 *Zea mays*
13	gil162459222 *Zea mays*
15	gil22284 *Zea mays*
17	gil22460 *Zea mays*

Spot	AC number (gi NCBI) and reference organism
21	gil162458484 *Zea mays*
24	gil75225211 *Oryza sativa* Japonica group
26	gil22284 *Zea mays*
29	gil162459533 *Zea mays*
36	gil162462359 *Zea mays*
38	gil168586 *Zea mays*

Spot	AC number (gi NCBI) and reference organism
40	
	gi|31322754 *Miscanthus x giganteus*
43	
	gi|22284 *Zea mays*
44	

Spot	AC number (gi NCBI) and reference organism
45	gi|162462211 *Zea mays*
49	gi|195651993 *Zea mays*
50	gi|194702870 *Zea mays*
51	gi|22284 *Zea mays*
	gi|62738111 *Zea mays*

Spot	AC number (gi NCBI) and reference organism
**52**	gi|168586 *Zea mays*
**53**	gi|162463106 *Zea mays*
**54**	

Spot	AC number (gi NCBI) and reference organism
55	gil195649437 *Zea mays*
59	gil194707628 *Zea mays*
61	gil195658529 *Zea mays*

Spot	AC number (gi NCBI) and reference organism
64	gi|157780962 *Zea mays*
66	gi|194688414 *Zea mays*
67	gi|162459678 *Zea mays*
69	gi|194699516 *Zea mays*

Spot	AC number (gi NCBI) and reference organism
74	gil6274486 *Saccharum officinarum*
78	gil194701990 *Zea mays*
83	gil194707628 *Zea mays*

Spot	AC number (gi NCBI) and reference organism
86	gil38347158 *Oryza sativa* (japonica cultivar-group)
87	gil37718900 *Oryza sativa*
96	gil6274486 *Saccharum officinarum*
104	gil194708240 *Zea mays*
	gil6274486 *Saccharum officinarum*

Spot	AC number (gi NCBI) and reference organism
107	
108	gil115446385 *Oryza sativa* (japonica cultivar-group)
111	gil50096951 *Oryza sativa*
112	gil194690156 *Zea mays*

Spot	AC number (gi NCBI) and reference organism
114	gil194701624 *Zea mays*
134	gil92429669 *Sorghum bicolor*
136	gil551288 *Zea mays*

Spot	AC number (gi NCBI) and reference organism
139	gil162458009 *Zea mays*
145	gil194707626 *Zea mays*
147	gil115679 *Zea mays*
148	gil115487910 *Oryza sativa*
	gil162458166 *Zea mays*

Spot	AC number (gi NCBI) and reference organism
**149**	
**154**	gil194688928 *Zea mays*
**163**	gil293887 *Zea mays*
**164**	gil162459678 *Zea mays*

Spot	AC number (gi NCBI) and reference organism
170	gi|162459533 *Zea mays*
172	gi|195639070 *Zea mays*
174	gi|194704818 *Zea mays*
175	
178	gi|115446385 *Oryza sativa* (japonica cultivar-group)

Spot	AC number (gi NCBI) and reference organism
179	gil162460908<br>*Zea mays*
185	gil75225211<br>*Oryza sativa*
186	gil75225211<br>*Oryza sativa*
188	gil22284<br>*Zea mays*
189	gil194701816<br>*Zea mays*
192	gil18483235<br>*Sorghum bicolor*
	gil162458671<br>Zea mays

Spot	AC number (gi NCBI) and reference organism
**196**	
	gil194703432<br>*Zea mays*
**197**	
**200**	
**203**	
	gil162461501<br>*Zea mays*
**205**	

Spot	AC number (gi NCBI) and reference organism
206	gil157830250<br>*Zea mays*
207	gil162458844<br>*Zea mays*
210	gil33321047<br>*Zea mays*
213	gil48374986<br>*Zea mays*
214	gil162461576<br>*Zea mays*
219	gil149798689<br>*Eriosorus cheilanthoides*

Spot	AC number (gi NCBI) and reference organism
242	gil22284 *Zea mays*
245	gil162459678 *Zea mays*
249	gil551288 *Zea mays*
251	gil162458671 *Zea mays*
252	gil53759189 *Saccharum officinarum*
257	
261	gil168664 *Zea mays*
262	

Spot	AC number (gi NCBI) and reference organism
265	gil115443643 *Oryza sativa*
266	gil20257707 *Zea mays*
267	
268	gil162464321 *Zea mays*
272	gil212276212 *Zea mays*
273	gil194689068 *Zea mays*
274	gil77378040 *Zea mays*

Spot	AC number (gi NCBI) and reference organism
276	gil168586 *Zea mays*
278	gil162458207 *Zea mays*
282	gil194701098 *Zea mays*
284	gil195636596 *Zea mays*
286	gil195635461 *Zea mays*

Spot	AC number (gi NCBI) and reference organism
287	gi|115466004 *Oryza sativa* (japonica cultivar-group)
292	gi|194693902 *Zea mays*
305	gi|293887 *Zea mays*

Spot	AC number (gi NCBI) and reference organism
309	gil194700662 *Zea mays*
311	gil194707626 *Zea mays*
312	gil194703432 *Zea mays*
318	gil118104 *Zea mays*

Spot	AC number (gi NCBI) and reference organism
**319**	gil50096951 *Oryza sativa*
**321**	gil162458207 *Zea mays*
**326**	gil162458207 *Zea mays*
**328**	gil195604800 *Zea mays*
**329**	gil194690236 *Zea mays*
**334**	gil195650645 *Zea mays*
**335**	gil194698866 *Zea mays*
**336**	gil195605060 *Zea mays*

Spot	AC number (gi NCBI) and reference organism
[illegible]	
337	gil195625602 *Zea mays*
338	gil195605254 *Zea mays*
339	gil195604800 *Zea mays*
340	gil212274479 *Zea mays*
	gil33321047

Spot	AC number (gi NCBI) and reference organism
	Zea mays
**342**	
**343**	gil195626982<br>*Zea mays*
**352**	gil195634861<br>*Zea mays*
**354**	gil22284<br>*Zea mays*
**356**	gil195657157<br>*Zea mays*

Spot	AC number (gi NCBI) and reference organism
357	gil4582787 *Zea mays*
358	gil162461063 *Zea mays*
	gil162458395 *Zea mays*

Spot	AC number (gi NCBI) and reference organism
**360**	
**362**	gil195638470 *Zea mays*
**363**	gil195654277 *Zea mays*
**368**	gil162460735 *Zea mays*
	gil195649437 *Zea mays*

Spot	AC number (gi NCBI) and reference organism
**376**	
	gil162460525<br>*Zea mays*
**380**	
**381**	gil75102649
**382**	gil194707992<br>*Zea mays*
	gil195658441<br>*Zea mays*
**385**	

Spot	AC number (gi NCBI) and reference organism
387	gil195654277<br>*Zea mays*
388	gil162460991<br>*Zea mays*
389	
392	gil194688950<br>*Zea mays*
394	gil195647178<br>*Zea mays*
395	gil212274681<br>*Zea mays*

Spot	AC number (gi NCBI) and reference organism
**398**	gil212720956 *Zea mays*
**399**	gil37147841 *Zea mays*
**403**	gil194707280 *Zea mays*

Table S3: BLAST results table

Spot n.	Protein	Blast results
**339**	Unknown	2-isopropylmalate synthase B
**328**	Unknown	2-isopropylmalate synthase B
**336**	Unknown	40S ribosomal protein S7
**340**	Hypothetical protein LOC100191561	Actin
**114**	Unknown	Adenine phosphoribosyl transferase
**112**	Unknown	Aldolase 1
**382**	Unknown	Ankyrin repeat domain-containing protein 2
**363**	Unknown	APx1 - Cytosolic Ascorbate Peroxidase
**387**	Unknown	APx1 - Cytosolic Ascorbate Peroxidase
**403**	Unknown	APx2 - Cytosolic Ascorbate Peroxidase
**148**	Os12g0230100	ATP dependent Clp protease
**86**	OSJNBa0039C07.4	ATP-dependent Clp protease ATP-binding subunit
**78**	Unknown	Carbonyl reductase 1
**50**	Unknown	Chitinase
**287**	Os06g0114000	Chaperonin 60 Beta
**104**	Unknown	Dessication-related protein
**329**	Unknown	DREPP4 protein
**108**	Os02g0519900	Elongation factor 2
**178**	Os02g0519900	Elongation factor 2
**174**	Unknown	Translation initiation factor 3 subunit 7
**69**	Unknown	Glucose and ribitol dehydrogenase homolog
**376**	Unknown	Heat shock 70 kDa protein
**272**	Hypothetical protein LOC100191552	Heat shock protein 17.9
**399**	Unknown	Heat shock protein STI
**342**	Unknown	Ketol-acid reductoisomerase
**292**	Unknown	Ketol-acid reductoisomerase
**213**	Hypothetical protein Z477F24.14	Lactoylglutathione lyase
**362**	Unknown	Malonyl CoA-acyl carrier protein transacylase
**189**	Unknown	Mitochondrial F0 ATP synthase D chain
**338**	Unknown	NADH ubiquinone oxidoreductase B22-like subunit
**154**	Unknown	NADH-ubiquinone oxidoreductase 75 kDa subunit
**335**	Unknown	Peroxiredoxin
**311**	Unknown	Phosphoglycerate kinase
**282**	Unknown	Plasminogen activator inhibitor 1 RNA-binding protein
**398**	Hypothetical protein LOC100193683	Proteasome subunit alpha type 2

Spot n.	Protein	Blast results
**286**	Unknown	Proteasome subunit alpha type 5
**66**	Unknown	Putative chaperonin 21 precursor
**309**	Unknown	Pyrophosphate-dependent phosphofructokinase beta subunit
**265**	Os02g0102900	RuBisCO large subunit-binding protein
**395**	Hypothetical protein LOC100191638	Salt tolerance protein
**343**	Unknown	Seed protein
**9**	Unknown	Splicing factor
**197**	Unknown	Stress responsive protein
**59**	Unknown	Stress responsive protein
**312**	Unknown	Stress responsive protein
**83**	Unknown	Stress responsive protein
**356**	Unknown	stromal 70 kDa heat shock-related protein
**273**	Unknown	superoxide dismutase3
**284**	Unknown	T-complex protein 1 subunit alpha
**352**	Unknown	Putative aminotransferase
**12**	Unknown	Transcription factor homolog
**392**	Unknown	UDP-glucose pyrophosphorylase
**385**	Unknown	Vacuolar ATP synthase catalytic subunit A
**262**	Unnamed protein product	Vacuolar protease A precursor

Table S3: BLAST results table

Spot n.	AC number (gi NCBI) and reference organism	% Identity	% Homology
**339**	gil195604800/ *Zea mays*	100%	100%
**328**	gil195604800/ *Zea mays*	100%	100%
**336**	gil195605060/ *Zea mays*	93%	96%
**340**	gil212274479/ *Zea mays*	98%	100%
**114**	gil194701624/ *Zea mays*	88%	94%
**112**	gil194690156/ *Zea mays*	99%	100%
**382**	gil194707992/ *Zea mays*	99%	99%
**363**	gil195654277/ *Zea mays*	100%	100%
**387**	gil195654277/ *Zea mays*	100%	100%
**403**	gil194707280/ *Zea mays*	99%	99%
**148**	gil115487910/ *Oryza sativa*	85%	92%
**86**	gil38347158/ *Oryza sativa*	90%	94%
**78**	gil194701990/ *Zea mays*	97%	97%
**50**	gil194702870/ *Zea mays*	99%	100%
**287**	gil115466004/ *Oryza sativa*	85%	91%
**104**	gil194708240/ *Zea mays*	60%	73%
**329**	gil194690236/ *Zea mays*	99%	99%
**108**	gil115446385/ *Oryza sativa*	96%	98%
**178**	gil115446385/ *Oryza sativa*	96%	98%
**174**	gil194704818/ Zea mays	99%	99%
**69**	gil194699516/ *Zea mays*	77%	88%
**376**	gil195649437/ *Zea mays*	100%	100%
**272**	gil212276212/ *Zea mays*	85%	90%
**399**	gil195635669/ *Zea mays*	90%	93%
**342**	gil195649547/ *Zea mays*	84%	90%
**292**	gil194693902/ *Zea mays*	99%	99%
**213**	gil48374986/ *Zea mays*	96%	98%
**362**	gil195638470/ *Zea mays*	100%	100%
**189**	gil194701816/ *Zea mays*	78%	92%
**338**	gil195605254/ *Zea mays*	100%	100%
**154**	gil194688928/ *Zea mays*	99%	100%
**335**	gil194698866/ *Zea mays*	79%	90%
**311**	gil194707626/ *Zea mays*	93%	97%
**282**	gil194701098/ *Zea mays*	98%	99%
**398**	gil212720956/ *Zea mays*	99%	100%

**Spot n.**	**AC number (gi NCBI) and reference organism**	**% Identity**	**% Homology**
**286**	gil195635461/ *Zea mays*	100%	100%
**66**	gil194688414/ *Zea mays*	80%	87%
**309**	gil194700662/ *Zea mays*	81%	89%
**265**	gil115443643/ *Oryza sativa*	91%	93%
**395**	gil212274681/ *Zea mays*	98%	100%
**343**	gil195626982/ *Zea mays*	72%	81%
**9**	gil194695412/ *Zea mays*	65%	75%
**197**	gil194703432/ *Zea mays*	76%	88%
**59**	gil194707628/ *Zea mays*	78%	89%
**312**	gil194703432/ *Zea mays*	76%	88%
**83**	gil194707628/ *Zea mays*	78%	89%
**356**	gil195657157/ *Zea mays*	100%	100%
**273**	gil194689068/ *Zea mays*	98%	99%
**284**	gil195636596/ *Zea mays*	100%	100%
**352**	gil195634861/ *Zea mays*	100%	100%
**12**	gil194695608/ *Zea mays*	93%	95%
**392**	gil194688950/ *Zea mays*	98%	99%
**385**	gil195658441/ *Zea mays*	100%	100%
**262**	gil171679543/ *Podospora anserina*	84%	92%

Pathway	Seqs in Pathway	Enzyme	Ezyme Id	Nr Seqs	Seqs	CTRL		MIC		Pathway Id
						spot ID new	trend	spot ID new	trend	
Carbon fixation in photosynthetic organisms	10	phosphoglycerate kinase	ec:2.7.2.3	1	gi|194707626	0080	↓	0080	↓	path:map00710
						0752	↓	0752	↓	
		pyruvate, phosphate dikinase	ec:2.7.9.1	4	gi|168586	0092	↓	0092	↓	
						0235	↓	0235	↓	
						0258	↓	0258	↓	
					gi|6274486	0249	↓	0249	↓	
						0137	↓	0137	↓	
						0215	↓	0215	↓	
					gi|31322754	0201	↓	0201	↓	
					gi|62738111	0252	↓	0252	↓	
		malate dehydrogenase (oxaloacetate-decarboxylating) (NADP+)	ec:1.1.1.40	1	gi|37147841	0390	↓	/	/	
		alanine transaminase	ec:2.6.1.2	1	gi|195625602	0578	↑	/	/	
		aspartate transaminase	ec:2.6.1.1	1	gi|195634861	0705	↓	0705	↓	
		malate dehydrogenase	ec:1.1.1.37	1	gi|162464321	0743	↓	0743	↓	
		fructose-bisphosphate aldolase	ec:4.1.2.13	1	gi|194690156	0708	↓	0708	↓	
Glycolysis / Gluconeogenesis	10	phosphoglycerate kinase	ec:2.7.2.3	1	gi|194707626	0080	↓	0080	↓	path:map00010
						0752	↓	0752	↓	
		phosphopyruvate hydratase	ec:4.2.1.11	2	gi|162458207	0148	↓	0148	↓	
						0584	↓	0584	↓	
						0664	↑	0664	↑	
					gi|162460735	0551	↓	0551	↓	
		6-phosphofructokinase	ec:2.7.1.11	1	gi|194700662	0070	↑	/	/	
		alcohol dehydrogenase (NADP+)	ec:1.1.1.2	1	gi|194699516	0832	↑	0832	↑	
		glyceraldehyde-3-phosphate dehydrogenase (phosphorylating)	ec:1.2.1.12	2	gi|293887	0160	↓	0160	↓	
						0019	↓	0019	↓	
					gi|162458671	0771	↓	0771	↓	
						0766	↓	0766	↓	
		phosphoglucomutase	ec:5.4.2.2	2	gi|162459678	0176	↓	0176	↓	
						0174	↓	0174	↓	
						0141	↓	/	/	
					gi|162463106	0472	↓	0472	↓	
		fructose-bisphosphate aldolase	ec:4.1.2.13	1	gi|194690156	0708	↓	0708	↓	
Citrate cycle (TCA cycle)	4	aconitate hydratase	ec:4.2.1.3	2	gi|75225211	0006	↓	0006	↓	path:map00020
						0175	↓	0175	↓	
						0231	↓	0231	↓	
					gi|92429669	0232	↓	0232	↓	
		malate dehydrogenase	ec:1.1.1.37	1	gi|162464321	0743	↓	0743	↓	
		succinate dehydrogenase (ubiquinone)	ec:1.3.5.1	1	gi|195647178	0101	↓	/	/	
Pyruvate metabolism	9	2-isopropylmalate synthase	ec:2.3.3.13	1	gi|195604800	0445	↓	0399	↓	path:map00620
		pyruvate, phosphate dikinase	ec:2.7.9.1	4	gi|168586	0092	↓	0092	↓	
						0235	↓	0235	↓	
						0258	↓	0258	↓	
					gi|6274486	0249	↓	0249	↓	
						0137	↓	0137	↓	
						0215	↓	0215	↓	
					gi|31322754	0201	↓	0201	↓	
					gi|62738111	0252	↓	0252	↓	
		malate dehydrogenase (oxaloacetate-decarboxylating) (NADP+)	ec:1.1.1.40	1	gi|37147841	0390	↓	/	/	
		malate dehydrogenase	ec:1.1.1.37	1	gi|162464321	0743	↓	0743	↓	
		lactoylglutathione lyase	ec:4.4.1.5	2	gi|162461576	0869	↑	0869	↑	
					gi|195639070	0820	↑	0820	↑	
Pentose phosphate pathway	5	ribokinase	ec:2.7.1.15	1	gi|162460525	0784	↓	0784	↓	path:map00030
		6-phosphofructokinase	ec:2.7.1.11	1	gi|194700662	0070	↑	/	/	
		phosphoglucomutase	ec:5.4.2.2	2	gi|162459678	0176	↓	0176	↓	
						0174	↓	0174	↓	
						0141	↓	/	/	
					gi|162463106	0472	↓	0472	↓	
		fructose-bisphosphate aldolase	ec:4.1.2.13	1	gi|194690156	0708	↓	0708	↓	
Glyoxylate and dicarboxylate metabolism	5	glycine transaminase	ec:2.6.1.4	1	gi|195625602	0578	↑	/	/	path:map00630
		aconitate hydratase	ec:4.2.1.3	2	gi|75225211	0006	↓	0006	↓	
						0175	↓	0175	↓	
						0231	↓	0231	↓	
					gi|92429669	0232	↓	0232	↓	
		malate dehydrogenase	ec:1.1.1.37	1	gi|162464321	0743	↓	0743	↓	
		catalase	ec:1.11.1.6	1	gi|115679	0145	↑	0145	↑	
Starch and sucrose metabolism	6	fructokinase	ec:2.7.1.4	1	gi|162460525	0784	↓	0784	↓	path:map00500
		UTP---glucose-1-phosphate uridylyltransferase	ec:2.7.7.9	1	gi|194688950	0570	↓	0570	↓	
		starch synthase (glycosyl-transferring)	ec:2.4.1.21	1	gi|33321047	0556	↓	/	/	
						0550	↓	0550	↓	
		phosphoglucomutase	ec:5.4.2.2	2	gi|162459678	0176	↓	0176	↓	
						0174	↓	0174	↓	
						0141	↓	/	/	
					gi|162463106	0472	↓	0472	↓	
		glycogen(starch) synthase	ec:2.4.1.11	1	gi|33321047	0556	↓	/	/	
						0550	↓	0550	↓	
		polygalacturonase	ec:3.2.1.15	1	gi|162458844	0991	↑	/	/	
Alanine, aspartate and glutamate metabolism	2	alanine transaminase	ec:2.6.1.2	1	gi|195625602	0578	↑	/	/	path:map00250
		aspartate transaminase	ec:2.6.1.1	1	gi|195634861	0705	↓	0705	↓	
		alanine---glyoxylate transaminase	ec:2.6.1.44	1	gi|195625602	0578	↑	/	/	
Cysteine and methionine metabolism	5	1-aminocyclopropane-1-carboxylate synthase	ec:4.4.1.14	1	gi|195625602	0578	↑	/	/	path:map00270
		5-methyltetrahydropteroyltriglutamate---homocysteine S-methyltransferase	ec:2.1.1.14	1	gi|18483235	0139	↓	/	/	
		methionine synthase	ec:2.1.1.13	1	gi|18483235	0139	↓	/	/	
		aspartate transaminase	ec:2.6.1.1	1	gi|195634861	0705	↓	0705	↓	
		aminocyclopropanecarboxylate oxidase	ec:1.14.17.4	2	gi|75225211	0006	↓	0006	↓	
						0175	↓	0175	↓	
						0231	↓	0231	↓	
					gi|92429669	0232	↓	0232	↓	
Purine metabolism	7	adenosine kinase	ec:2.7.1.20	1	gi|4582787	0739	↓	/	/	path:map00230
		adenosinetriphosphatase	ec:3.6.1.3	2	gi|37718900	0172	↓	/	/	
					gi|162458166	0193	↓	0193	↓	
		nucleoside-diphosphate kinase	ec:2.7.4.6	1	gi|50096951	1283	↑	1283	↓	
					gi|50096951	/	/	0065	↓	
		adenylate kinase	ec:2.7.4.3	1	gi|4582787	0739	↓	/	/	
		adenine phosphoribosyltransferase	ec:2.4.2.7	1	gi|194701624	0046	↑	0046	↑	
		phosphoglucomutase	ec:5.4.2.2	2	gi|162459678	0176	↓	0176	↓	
						0174	↓	0174	↓	
						0141	↓	/	/	
					gi|162463106	0472	↓	0472	↓	
Valine, leucine and isoleucine biosynthesis	3	2-isopropylmalate synthase	ec:2.3.3.13	1	gi|195604800	0445	↓	0399	↓	path:map00290
		acetolactate synthase	ec:2.2.1.6	1	gi|75102649	0449	↓	/	/	
		ketol-acid reductoisomerase	ec:1.1.1.86	1	gi|194693902	0559	↓	/	/	

Table S4: Kegg table
